# Systematic Analysis of the Gene Expression in the Livers of Nonalcoholic Steatohepatitis: Implications on Potential Biomarkers and Molecular Pathological Mechanism

**DOI:** 10.1371/journal.pone.0051131

**Published:** 2012-12-26

**Authors:** Yida Zhang, Susan S. Baker, Robert D. Baker, Ruixin Zhu, Lixin Zhu

**Affiliations:** 1 Department of Bioinformatics, Tongji University, Shanghai, P.R. China; 2 Digestive Diseases and Nutrition Center, Department of Pediatrics, the State University of New York at Buffalo, Buffalo, New York, United States of America; Bambino Gesu' Children Hospital, Italy

## Abstract

Non-alcoholic steatohepatitis (NASH) is a severe form of non-alcoholic fatty liver disease (NAFLD). The molecular pathological mechanism of NASH is poorly understood. Recently, high throughput data such as microarray data together with bioinformatics methods have become a powerful way to identify biomarkers and to investigate pathogenesis of diseases. Taking advantage of well characterized microarray datasets of NASH livers, we performed a systematic analysis of potential biomarkers and possible pathological mechanism of NASH from a bioinformatics perspective.

CodeLink Human Whole Genome Bioarrays were analyzed to find differentially expressed genes (DEGs) between controls and NASH patients. Four methods were used to identify DEGs and the intersection of DEGs identified by these methods was subsequently used for both biomarker prediction and molecular pathological mechanism analysis. For biomarker prediction, rank aggregation was used to rank DEGs identified by all these methods according to their significance of different expression. *Alcohol dehydrogenase 4 (ADH4)* exhibited the highest rank suggesting the most significant differential expression between normal and disease condition. Together with the previous report demonstrating the association between *ADH4* and the pathogenesis of NASH, our data suggest that *ADH4* could be a potential biomarker for NASH. For molecular pathological mechanism analysis, two clusters of highly correlated annotation terms and genes in these terms were identified based on the intersection of DEGs. Then, pathways enriched with these genes were identified to construct the network. Using this network, both for the first time, amino acid catabolism is implicated to play a pivotal role and urea cycle is implicated to be involved in the development of NASH.

The results of our study identified potential biomarkers and suggested possible molecular pathological mechanism of NASH. These findings provide a comprehensive and systematic understanding of the pathogenesis of NASH and may facilitate the diagnosis, prevention and treatment of NASH.

## Introduction

Non-alcoholic fatty liver disease consists of a spectrum of disease ranging from simple steatosis (SS) which generally follows a benign non-progressive clinical course, to NASH which may progress to cirrhosis and hepatocellular carcinoma [1], [2], [3]. The term NASH was first introduced by Ludwig *et al*. in 1980 [4]. It described subjects who did not consume alcohol but had progressive liver disease similar to those with alcoholic hepatitis. In addition, there is a correlation between NASH and obesity, type 2 diabetes, hyperlipidemia and other lifestyle-related diseases [5]. In accordance with the dramatic rise of population levels of obesity and diabetes, NASH now becomes one of the most common causes of liver disease in the Western world [6], [7], [8].

In recent years, a growing body of evidence has suggested that “two-hits” are the prerequisites of the development of NASH [7], [9], [10]. The first hit corresponds to the fat accumulation in liver and the second hit consists of an oxidative stress leading to liver injury and inflammation. What is more, a number of studies showed that apoptosis [11], [12], mitochondrial dysfunction [13], [14], [15], [16], [17], insulin resistance [18], [19], [20], [21], immune response [21], [22], alcohol metabolism [23], lipid peroxidation [5], [24], lipid metabolism [25], and many other factors like endoplasmic reticulum's response to stress [9] are all involved in or related to the development of NASH. However, the complex interplay among these observations and the molecular pathological mechanism of NASH remains unknown [26].

The diagnosis of NASH relies on a number of clinical and laboratory tests. NASH, with few exceptions, occurs in the context of obesity [24]. Because hepatic steatosis is a hallmark of NASH [21], tests that show fat within the liver strongly support the diagnosis of NASH. For instance, ultrasound can show hyperechoic pattern consistent with fat within the liver, but processes other than fatty infiltration can produce a similar picture. Liver biopsy is considered the best way of identifying fat within the liver, although it is invasive and can miss inhomogeneous fat distribution which may lead to sampling errors [5], [27]. Liver biopsy can demonstrate liver inflammation and fibrosis, two other characteristic findings in NASH [8]. Because hepatitis is another hallmark of NASH, elevated transaminases support NASH as a diagnosis. Because no single parameter can establish the diagnosis of NASH and because the tests used for NASH are invasive, inaccurate and/or expensive, a biomarker would facilitate establishing a diagnosis.

A great deal of effort, unsuccessful to date, has been expended to identify the pathogenesis of NASH and to find biomarkers for NASH. Although some studies [25] have investigated more than one pathway at a time, there is no systematic study of the pathogenesis of NASH. In order to study the molecular pathological mechanism and to find possible biomarker(s) of NASH from a more systematic perspective, two sets of whole genome microarrays were used and analysis from a bioinformatics perspective was performed. First, four representative methods (Significance Analysis of Microarrays, Weighted Average Difference method, *t*-test and Wilcoxon rank sum test) were used to find DEGs in the two sets of microarrays. Among these methods, Significance Analysis of Microarrays and *t*-test are parametric tests based on *t* statistic. Weighted Average Difference method is a parametric test based on fold change. Wilcoxon rank sum test is a non-parametric test. Since these methods are based on different theories, the intersection of DEGs identified by these different methods can ensure that the different expression of these genes were “true” different expression instead of errors. However, the same gene in the intersection may have different ranks in the original DEG lists generated by different methods, so to rank genes in the intersection based on their ranks in the original DEG lists, rank aggregation was performed. The iterative procedure of rank aggregation can guarantee a better aggregation performance than simply using the average of ranks of a gene to rank that gene. After rank aggregation, the result was used to find the gene with the most significant difference of expression. Together with previous studies and knowledge of biochemistry and molecular biology, the biomarker can be predicted with more confidence. Parallel to the rank aggregation, functional analysis was carried out based on the intersection of DEGs to illustrate the underlying pathological mechanism and elucidate the complex interplay between different pathways. Among the intersection of DEGs, genes enriched in highly correlated annotation terms were identified. After this, different from network construction in previous studies, these genes were not used directly to construct the network. Instead, pathways in which these genes were enriched were used for the network construction. The pathways in the network not only cross-validated each other but also agreed with results from previous studies. As a result, the final interaction network gives us a systematic view of not only the possible molecular pathological mechanism of NASH, but also the interplay among different pathways involved in NASH livers. Taken together,these results provide a possible biomarker and add to our understanding of the pathogenesis of NASH.

## Materials and Methods

### Workflow

**Figure 1:**
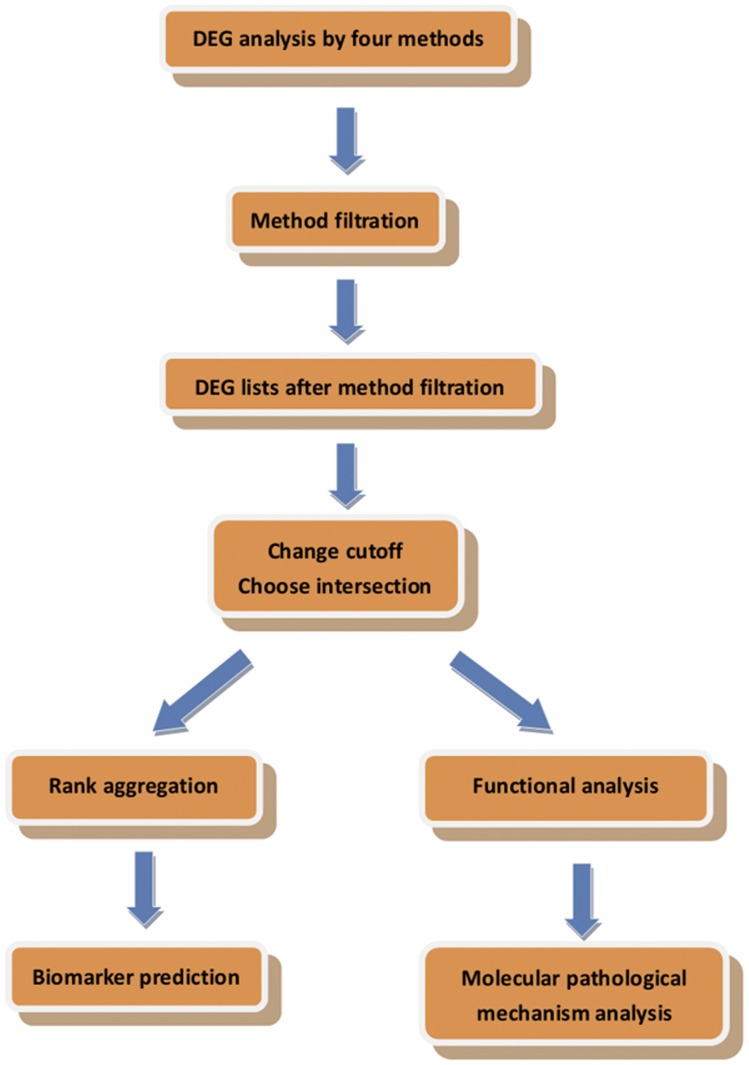
Workflow. The workflow of this article.

1. Use four representative methods to find DEGs in the two sets of microarrays respectively.
2. Use DEGs reported in [23], [25], [28] as a reference to filter out methods with poor performance.
3. For methods which are not filtered out in step 2, choose more stringent cutoffs to focus on more significantly changed genes and use the intersection of DEGs to do functional analysis and rank aggregation.
4. The first step of functional analysis, functional annotation clustering in DAVID, is carried out to find highly correlated annotation terms which were also significantly enriched with the DEGs identified by methods not filtered out in step 2. Results of the two microarrays are analyzed respectively in DAVID. In the second step, information in KEGG pathway is used to find pathways in which genes in the annotation terms are involved. After this, enrichment scores are calculated for all these pathways. Only pathways which are significantly enriched with genes in the annotation terms are used to construct the final interaction network which ensures that all the pathways in the network are highly correlated with NASH. The enriched pathways identified in both the two microarrays are combined together in this process.
5. Parallel to functional analysis, rank aggregation is used to rank DEGs identified by all methods not filtered out in step 2 to find the gene with the most significant different expression. This is done in the two microarrays respectively.
6. Finally, the ranking results in the two microarrays are used to predict potential biomarkers of NASH and the interaction network is used to analyze the molecular pathological mechanism of NASH. **Figure 1** summarizes the workflow.

### Dataset

#### Microarray one

The first microarray was downloaded from Gene Expression Omnibus (GEO) website: http://www.ncbi.nlm.nih.gov/geo/ [29]. This dataset includes 11 individual microarray experiments. The accession numbers for 7 NASH liver datasets are GSM435821 to GSM435827 which corresponds to 7 NASH patients (P53, P55, P59, P35, P37, P40, P41). For 4 normal control datasets, the accession numbers are GSM435828, GSM435833 to GSM435835. The corresponding samples are A486, A643, A138 and A249. Microarray one was used in the study of lipid and alcohol metabolism. The age range of the patients and controls is 2 months to 19 years old.

#### Microarray two

The second microarray was also downloaded from GEO. This dataset includes 17 experiments: 12 NASH and 5 normal controls. 7 of these NASH patients and 2 controls (A486 and A643) are the same patients from microarray one. The additional 5 NASH patients are P34, P51, P62, P64 and P66. The additional 3 controls are A99, A107 and A154. The GEO accession number for this dataset is GSE24807. It was used in the study of hemoglobin. Infants were excluded from this experiment. The age range of the patients and controls is 5 to 19 years old.

### Four methods for the identification of DEGs

#### Significance Analysis of Microarrays

Significance Analysis of Microarrays (SAM) [30] is a widely used statistical method for identifying DEGs between experimental groups [31]. It identifies DEGs by assimilating a set of gene-specific *t*-test. SAM will calculate the “relative difference” 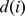 for each gene based on the change in gene expression relative to the standard deviation of repeated measurements. The “relative difference” 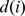 is:
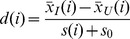
(1)where 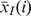 and 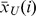 are defined as the average levels of expression for gene 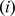 in states I and U, respectively. The “gene-specific scatter” 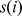 is the standard deviation of repeated expression measurements:
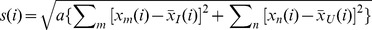
(2)where 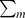
and 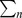
are summations of the expression measurements in states I and U, respectively. 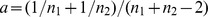, and 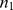
and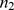 are the numbers of measurements in states I and U.

Genes that have relative differences greater than the threshold are thought to be potentially significant and permutations will be used for the repeated measurements to estimate the false discovery rate (FDR) [30]. SAM modifies the *t*-test by adding a small positive constant 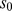 to the denominator of the *t* statistic [32] to ensure that the distribution of 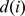 is independent of the gene expression level so that we can compare values of 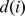 across all genes.

In order to increase the statistical confidence, a large number of controls are generated by computing relative differences from permutations of the hybridizations for state I and state U.

#### Weighted Average Difference method

The weighted average difference method (WAD) [32] is a fold-change based method for ranking DEGs. The basic assumption of WAD is that “strong signals are better signals” which is in accordance with the observation that known or potential marker genes or proteins tend to have high expression levels. The WAD performs the best in the comparison with other statistical methods for ranking DEGs conducted by [33] and [32] on different microarray platforms and under different preprocessing algorithms.

The weighted average difference statistic for the 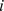
th gene, 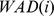, is calculated as:
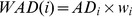
(3)where 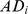 is the average difference for the 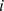
th gene and 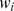 is a relative average log signal intensity to weight the average difference in 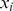 so that genes exhibiting lower expression levels will not have a high rank [30].


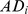
 can be calculated as:
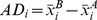
(4)where 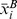 is the average log signal for all class B replicates and 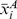 is the average log signal for all class A replicates. This is an obvious indicator for estimating the differential expression of the 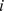
th gene, 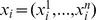.


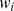
 can be calculated as:
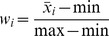
(5)where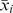 is calculated as (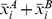)/2, and the max (or min) indicates the maximum (or minimum) value in an average expression vector 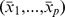 on a log scale.

In our study, we calculated the absolute value of the average difference for each gene. The cutoff was set to 1.5 in method filtration. Genes with absolute average difference higher than the cutoff were considered significant. Since the cutoff is fairly stringent, we kept the cutoff unchanged when doing rank aggregation and functional analysis.

#### t-test

Since *t*-test has a good performance in our previous studies [23], [25], [28], it is incorporated in this article. *t*-test is a classical statistical hypothesis test in which the test statistic follows a Student's *t* distribution under null hypothesis. Two sample *t*-test is used in our study to find genes with different expression between control and NASH samples.

In method filtration, genes with p-value less than 0.05 were considered significant which was in accordance with the criterion used in the three reference papers [23], [25], [28]. But when using the result for rank aggregation and functional analysis, we lowered the p-value cutoff to 0.01 to reduce the amount of data and focus on a more significant part of the result.

#### Wilcoxon rank sum test

The Wilcoxon rank sum test is a nonparametric alternative to the two sample *t*-test which is based on the order in which the observations from the two samples fall. Because it operates on rank-transformed data, it is a robust choice for microarray data, which are often non-normal and contain outliers [34]. What is more, previous studies suggest that using rank-transformed data in microarray analysis is advantageous [35], [36]. It is also a conservative algorithm which is good when the computationally identified genes need to be tested biologically [34].

Assuming we have two groups of data, 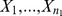 in group A and 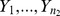 in group B.

The whole procedure is as following:

1. Combine the two samples into one sample and order the data in the combined sample.
2. Assign rank 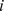 to the 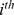 smallest observation. If there are some observations tied with the same value, we assign the average rank to each observation.
3. Calculate the sum of ranks attached to observation 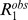 in sample A
4. Calculate
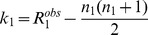
(6)
5. Calculate
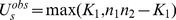
(7)

Find the distribution of 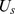 under 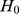
(probability distributions for 2 sampled populations are identical). Reject if
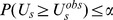
(8)


In method filtration, genes with p-value less than 0.05 were considered to be significantly differentially expressed which was the same with the criterion in the three reference papers. Similarly for the *t*-test, we lowered the p-value cutoff to 0.01 when doing rank aggregation and functional analysis.

### Rank aggregation

Rank aggregation is a method for combining several ordered lists in a proper and efficient manner. Rank-based aggregation can combine lists regardless of the sources or platforms from which they are generated. The ultimate goal of it is to find a “super list” which is as “close” as possible to all individual ordered lists simultaneously [37]. To measure the “closeness”, an object function is defined:
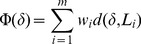
(9)where 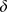 is an ordered list of length 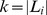, 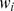 is the importance weight associated with list 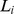, 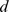 is a distance function which we will discuss later, and 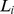 is the 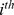 ordered list. The idea of rank aggregation is to find 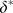 which can minimize the total distance between 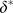
and 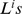:
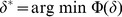
(10)


There are two different philosophies on rank aggregation. The first one is based on the majoritarian principles which put more weight on the majority of individual preferences than on infrequent ones so that the final rank is usually based on the number of pairwise wins between items within individual lists. For example, if item “A” more often has a higher rank than item “B”, then item “A” should have a higher rank than item “B” in the final list. The other philosophy attempts to seek the consensus among individual lists and is usually based on some form of rank averaging [37].

Under the two philosophies, there are many rank aggregation methods like Cross-Entropy Monte Carlo algorithm (CE) and Genetic algorithm (GA). Also, there are many ways to calculate the distances between different ordered lists. The most popular distance functions are Spearman footrule distance, Kendall's tau distance and the weighted version of these two methods. Since the weighted Spearman footrule distance is quite simple and can incorporate quantitative information, and CE has a good performance in many studies [37], [38], [39], we used CE together with weighted Spearman footrule distance to do the rank aggregation.

Before introducing weighted Spearman footrule distance, there are some necessary notations. 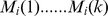 are scores associated with the ordered list 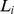. For example, 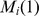 is the best score. 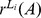 is the rank of A in the list 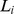 if A is within the top 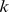, and be equal to 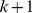, otherwise; 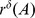 is defined the same way. Weighted Spearman's footrule distance between 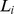 and any ordered list 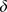 can be defined as
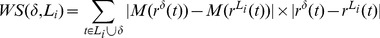
(11)


As to CE, it is a 2-step “simulate-update” iterative procedure:

1. Generate a random sample from the probability mass function of a random matrix.
2. Update parameters based on the drawn sample to produce a “better” sample.

It includes four main steps. Details can be seen in [39]. Below is a brief description of the four main steps:

1. **Initialization:** generate the uniform multinomial cell probabilities.
2. **Sampling:** during each round, with the current cell probabilities generated in the first step, generate a random sample via multinomial sampling.
3. **Updating:** update the multinomial cell probabilities based on the current sample and the value of the objective function so that the objective function in the next round will be smaller.
4. **Convergence:** when the smallest values of the objective function do not change during a number of iterations, stop the search.

In our study, the intersection of DEG lists generated by DEG identifying methods not filtered out in method filtration was used as the input of rank aggregation. This was done in the two sets of microarrays respectively. The weighted Spearman footrule distance and the iterative procedure of CE used in rank aggregation can make good use of the statistics (p-value and weighted average difference) of each gene and ensure a better aggregation result compared with simply using the average of ranks to rank a gene.

### Functional analysis

DAVID is an integrated biological knowledge-base and analytic tool which can be used to extract biological information from gene lists [40], [41]. In our study, we used the functional annotation clustering module to help us find out which cluster contains annotation terms not only significantly enriched with DEGs identified by DEG identifying methods not filtered out in method filtration, but also highly correlated with each other. This was done in the two sets of microarrays respectively. After identifying the significantly enriched and highly correlated annotation terms together with genes in these terms, information in KEGG [42], [43], [44] was used to find the pathways in which these genes were involved. Compared with using all genes in the intersection of DEGs to identify pathways, using genes in the annotation terms is powerful because it can detect the most significantly changed and highly correlated pathways between normal controls and NASH patients. Among these pathways, we used fisher's exact test to identify pathways in which genes in the annotation terms were enriched. P-value was calculated for each pathway. The smaller the p-value, the less likely an observed proportion of genes mapping to a pathway is a result of chance. Finally, enriched pathways identified in both the two microarrays were combined together to construct the network.

Of note, to make the network more informative and accurate, we modified the network from three perspectives. First, information in KEGG only gave a brief summary of pathways and this can be further divided into more specific pathways. For example, the term “peroxisome” was found by KEGG. However, the exact pathways in “peroxisome” are fatty acid oxidation (including alpha and beta oxidation), amino acid metabolism and hydrogen peroxide metabolism. This division was done manually for all the enriched pathways before using them to construct the network. Second, some pathways are isolated with other pathways. Incorporating them into the final network requires too much additional information unrelated to the result found in our study. These isolated pathways were excluded in the network construction. Third, some pathways enriched with no genes in the two clusters were incorporated into the final network since the result of our study infers a strong association between them and pathways enriched with genes in the two clusters. This process makes the network more informative and comprehensive and these pathways can be used to guide further analysis.

## Results

### Method filtration

Initially four methods were employed to identify DEGs. These methods were then evaluated and compared, using DEGs validated in our previous studies [23], [25], [28] as the criterion. These DEGs are the only reported NASH related experimental data used in the statistical analyses performed in this study. The cutoff for *t*-test and Wilcoxon rank sum test was set to 0.05 which was the same cutoff in three reference papers. However, WAD and SAM do not use p-value as the cutoff so that we cannot define a cutoff equivalent to 0.05. In addition, a method covering only a small portion of the DEGs reported in the three reference papers may cover more DEGs reported in other papers or even DEGs unreported. Therefore, we did not use the number of DEGs each method covers to compare these methods. Instead, we used the difference of the three pathways between the two microarrays as the standard. For microarray one which was used to study alcohol and lipid metabolism, NASH patients exhibiting insulin resistance were selected. But in the second microarray which was used to study hemoglobin, the selection method was changed. Since infants have significant expression of hemoglobin during early development, liver biopsies from older children and a more stringent standard for age-matching were used. Consequently, since the expression of hemoglobin is associated with age but the included age range has no influence on alcohol and lipid metabolism, the expression profile of genes related to hemoglobin should be different between the two sets of microarrays and the expression profile of genes related to lipid and alcohol metabolism respectively are expected to be similar. This difference is used as the criterion to filter DEG identifying methods. In this way, the cutoff will have no influence on the filtration result. However, we still choose 0.05 as the cutoff for *t*-test and Wilcoxon rank sum test since this cutoff was applied in our previous study of lipid metabolism, alcohol metabolism and hemoglobin.

**Table 1** and **Table 2** show that when considering DEGs related to alcohol metabolism (15 major genes involved in alcohol metabolism in total) and lipid metabolism (19 major genes involved in lipid metabolisms in total), all four methods performed similarly between the two microarrays. **Table 3** show that when considering DEGs related to hemoglobin (2 major genes in total), the expression profile calculated by WAD, Wilcoxon rank sum test and *t*-test are different between the two microarrays. However, SAM failed to show this difference. These results indicate that SAM cannot detect the difference between microarray one and microarray two, and therefore was not used in the downstream analyses. The DEGs identified by other three methods were used to perform the functional analysis. The DEG lists of four DEG identifying methods are presented in **Tables S1, S2, S3, S4, S5, S6, S7, S8, S9, and S10**. The detailed information of DEGs related to lipid metabolism, alcohol metabolism and hemoglobin found by these four methods is presented in **Tables S11, S12, S13, S14, S15, S16, S17, and S18**.

**Table 1:** Number of DEGs related to alcohol metabolism found by four methods.

method	Number of DEGs related to alcohol metabolism	
	Microarray one	Microarray two
**WAD**	6	4
**Wilcoxon rank sum test**	13	14
***t*** **-test**	13	12
**SAM**	3	2

**Table 2:** Number of DEGs related to lipid metabolism found by four methods.

method	Number of DEGs related to lipid metabolism	
	Microarray one	Microarray two
**WAD**	5	3
**Wilcoxon rank sum test**	15	15
***t*** **-test**	13	13
**SAM**	0	0

**Table 3:** Number of DEGs related to hemoglobin found by four methods.

method	Number of DEGs related to hemoglobin	
	Microarray one	Microarray two
**WAD**	1	2
**Wilcoxon rank sum test**	0	2
***t*** **-test**	0	2
**SAM**	0	0

### Functional analysis

After the method filtration, DEGs identified by WAD, Wilcoxon rank sum test and *t*-test were used for the functional analysis. To reduce the amount of genes and focus on a more significant part of the result, we lowered the cutoff for Wilcoxon rank sum test and *t*-test from 0.05 to 0.01. The cutoff for WAD was unchanged as it was already stringent. DEG lists of Wilcoxon rank sum test and *t*-test under the new cutoff are presented in **Tables S19, S20, S21, and S22**. To ensure that all the DEGs are not identified by accident, the intersection of DEGs identified by all three methods were used as the input for the functional analysis [34], [45]. The intersection of DEGs was uploaded onto DAVID and the functional annotation clustering module was used to find clusters of significantly enriched and highly correlated annotation terms given the uploaded gene list. Since the intersection of DEGs of microarray one and two were uploaded and analyzed respectively, we obtained two lists of clusters containing significantly enriched and highly correlated annotation terms. The clusters in the two lists were ranked in descending order according to the degree of enrichment and correlation of annotation terms. **Table 4** shows information of the cluster (cluster 1) ranked the 1^st^ in microarray one and **Table 5** shows information of the cluster (cluster 2) ranked the 2^nd^ in microarray two. Detailed information about genes involved in cluster 1 and cluster 2 are listed in **Table 6** and **Table 7**. Since the annotation terms in cluster 1 and cluster 2 were almost identical indicating that these terms were significantly enriched and highly correlated in both of the two microarrays, we chose proteins encoded by these two clusters of genes and pathways enriched with these proteins to construct the network using information in KEGG pathway database. The cluster ranked the 1^st^ in microarray two was not used in functional analysis since the terms it contained cannot be found in the clustering result of microarray one. Because the expression of genes in cluster 1 and cluster 2 were all elevated in NASH patients, pathways containing these genes were considered up-regulated. The complete results of functional annotation clustering analysis are presented in **Tables S23 and S24**. Besides, DEGs lists of three methods not filtered out in method filtration in both microarrays were also uploaded onto DAVID for the functional annotation clustering analysis. Results of these DEG lists are presented in **Tables S25, S26, S27, S28, S29, and S30**. Since the DEG list of Wilcoxon rank sum test of microarray two contained too many genes to do functional annotation clustering analysis, we used the functional annotation chart module to analyze it instead.

**Table 4:** The information of cluster 1.

Annotation Cluster 1		Enrichment Score: 5.3158	
Category	Term	P-value	Genes^1^
UP_SEQ_FEATURE	short sequence motif:Microbody targeting signal	9.62E-07	NM_153756,NM_001917,NM_006117, NM_016518,NM_018441,NM_001966
SP_PIR_KEYWORDS	peroxisome	2.69E-06	NM_153756,NM_001917,NM_006117, NM_016518,NM_018441,NM_001752, NM_001966
GOTERM_CC_FAT	GO:0005777∼peroxisome	1.45E-05	NM_153756,NM_001917,NM_006117, NM_016518,NM_018441,NM_001752, NM_001966
GOTERM_CC_FAT	GO:0042579∼microbody	1.45E-05	NM_153756,NM_001917,NM_006117, NM_016518,NM_018441,NM_001752, NM_001966

1 :All the accession numbers in this article are from GenBank database.

**Table 5:** The information of cluster 2.

Annotation Cluster 2		Enrichment Score: 4.3758	
Category	Term	P-value	Genes
SP_PIR_KEYWORDS	peroxisome	3.64E-06	NM_006821,NM_153756,NM_001917, NM_006117,NM_018663,NM_016518, NM_018441,NM_006214
UP_SEQ_FEATURE	short sequence motif:Microbody targeting signal	1.07E-05	NM_006821,NM_153756,NM_001917, NM_006117,NM_016518, NM_018441
GOTERM_CC_FAT	GO:0042579∼microbody	2.84E-04	NM_153756,NM_001917,NM_006117, NM_018663,NM_016518,NM_018441, NM_006214
GOTERM_CC_FAT	GO:0005777∼peroxisome	2.84E-04	NM_153756,NM_001917,NM_006117, NM_018663,NM_016518,NM_018441, NM_006214

**Table 6:** Detailed information of genes in cluster 1.

Accession number	Gene name	Rank in rank aggregation	Gene symbol	Gene ID
NM_153756	fibronectin type III domain containing 5	3	FNDC5	252995
NM_001917	D-amino-acid oxidase	53	DAO	1610
NM_006117	peroxisomal D3,D2-enoyl-CoA isomerase	77	ECI2	10455
NM_016518	pipecolic acid oxidase	125	PIPOX	51268
NM_018441	peroxisomal trans-2-enoyl-CoA reductase	117	PECR	55825
NM_001752	catalase	88	CAT	847
NM_001966	enoyl-Coenzyme A, hydratase/3-hydroxyacyl Coenzyme A dehydrogenase	99	EHHADH	1962

**Table 7:** Detailed information of genes in cluster 2.

Accession number	Gene name	Rank in rank aggregation	Gene symbol	Gene ID
NM_006821	acyl-CoA thioesterase 2	182	ACOT2	10965
NM_153756	fibronectin type III domain containing 5	10	FNDC5	252995
NM_001917	D-amino-acid oxidase	116	DAO	1610
NM_006117	peroxisomal D3,D2-enoyl-CoA isomerase	171	ECI2	10455
NM_018663	hypothetical LOC100129532; peroxisomal membrane protein 2, 22 kDa	102	PXMP2	5827
NM_016518	pipecolic acid oxidase	176	PIPOX	51268
NM_018441	peroxisomal trans-2-enoyl-CoA reductase	172	PECR	55825
NM_006214	phytanoyl-CoA 2-hydroxylase	21	PHYH	5264

**Figure 2:**
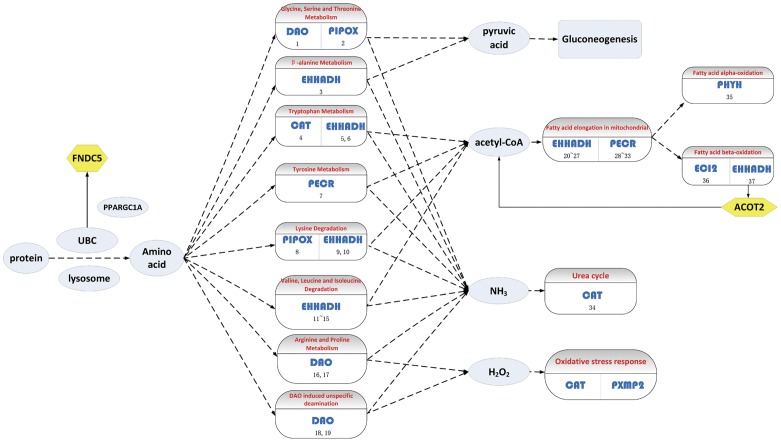
Interaction network of pathways. After functional annotation clustering analysis in DAVID, genes in the two clusters of annotation terms along with KEGG pathway information of these genes were used to construct the interaction network. Genes together with proteins encoded by these genes were classified into several main pathways before constructing this network. Reactions in which these genes were involved were also incorporated in the network. Square frames represent pathways which contain proteins encoded by genes in the two clusters. These proteins involved in a particular pathway are written in the square frame of that pathway. The number corresponding to each protein represents a reaction in which that protein is involved. The reaction equation can be referred to in Table S31 by the number of that reaction. These proteins serve as catalysts. Ovals represent other genes, proteins or molecules involved in this network. The rectangle represents a pathway with no genes or proteins in the two clusters in it. Yellow hexagons represent proteins encoded by genes in the two clusters which cannot be classified into a particular pathway. Solid lines indicate direct connections and dashed lines indicate indirect connections.

**Figure 2** is the interaction network of pathways together with reactions involved in these pathways. Each reaction equation is represented by a number. The detailed information of each reaction equation is provided in **Table S31**. The original network with reactions on it is presented in **Figure S1**. The interaction network of genes in the two clusters and proteins encoded by these genes before being classified into pathways is presented in **Figure S2**, in which the interplay of genes and proteins instead of pathways was shown.

### Potential biomarker

A biomarker is defined as a characteristic that is objectively measured and evaluated as an indicator of normal biologic processes, pathogenic processes or pharmacologic responses to a therapeutic intervention. It can be specific cells, molecules, or genes, gene products, enzymes or hormones. Since microarrays were used as the main approach to find biomarkers in our study, we identified a biomarker at mRNA level.

A biomarker must easily detect differential expression between normal and abnormal conditions. This means that the greater the difference of expression values between two conditions, the more likely the measure qualifies as a biomarker. To meet this demand, rank aggregation was performed to rank genes identified as differentially expressed by Wilcoxon rank sum test, WAD and *t*-test. This was done separately in microarray one and microarray two. The weighted Spearman footrule distance and the iterative procedure of CE used in rank aggregation guarantees a reliable result. The ranking results are presented in **Tables S32 and S33**. The gene with the highest rank is considered as the gene with the greatest expression difference. **Table 8** shows the top 5 genes in microarray one and microarray two respectively. In both microarray one and microarray two, *ADH4* which encodes alcohol dehydrogenase 4 (class II), pi polypeptide showed very high rank indicating that *ADH4* is the most significantly differentially expressed gene. More specifically, the expression of *ADH4* was significantly up-regulated in NASH patients compared with normal controls.

**Table 8:** Top 5 genes after rank aggregation in microarray one and two.

Microarray one		Microarray two	
Accession number	Gene name	Accession number	Gene name
NM_003122	serine peptidase inhibitor, Kazal type 1	**NM_000670**	**alcohol dehydrogenase 4 (class II), pi polypeptide**
**NM_000670**	**alcohol dehydrogenase 4 (class II), pi polypeptide**	AK023341	Nicotinamide phosphoribosyltransferase
NM_153756	fibronectin type III domain containing 5	NM_000394	crystallin, alpha A
NM_003986	butyrobetaine (gamma), 2-oxoglutarate dioxygenase (gamma-butyrobetaine hydroxylase) 1	NM_004887	chemokine (C-X-C motif) ligand 14
NM_003251	thyroid hormone responsive	NM_016246	hydroxysteroid (17-beta) dehydrogenase 14

Apart from being highly differentially expressed, a biomarker should also be able to detect the presence of the disease [26]. In other words, the change of the biomarker should be highly correlated with the pathogenesis of the disease. We previously demonstrated that alcohol metabolism plays a major role in the pathogenesis of NASH [23]. Additionally, ADH4 is the major hepatic alcohol dehydrogenase. All these facts indicate a high correlation between *ADH4* and NASH. Along with the highly differential expression between normal and disease conditions, we hypothesize that *ADH4* is a potential biomarker for NASH.

### Top five ESTs after rank aggregation

Since the microarrays used in our study are whole genome microarrays, expressed sequence tags (ESTs) are included. We did not exclude ESTs when doing the rank aggregation since further study of these ESTs may provide us more useful information. UniGene database and BLAST in NCBI were used to analyze these ESTs. UniGene database is a gene-oriented view of sequence entries developed at NCBI. Information on protein similarities, gene expression and genomic location is included within each entry. Most importantly, information of uncharacterized ESTs is also included in this database. These uncharacterized ESTs are clustered based on Megablast. Apart from UniGene, BLAST was also used to find sequences with known functions similar to uncharacterized ESTs. The information provided by UniGene and BLAST can facilitate the study of functions of these ESTs and may also provide new clues for the pathogenesis of NASH. However, since function prediction of ESTs is beyond the scope of this article, we present information on the top five ESTs in the two microarrays respectively in **Tables S34 and S35**.

## Discussion

### The difference between microarray one and microarray two

Chuaqui *et al*. [46] raised two important questions concerning microarray data analysis. The first question is whether the result is valid or accurate. In our study, we chose four representative methods and filtered out one due to unsatisfactory performance in the identification of DEGs and we used the intersection of DEGs generated by the other three methods to lend more credibility to the conclusion that these genes are differentially expressed regardless of the method used. Moreover, the whole genome microarrays used in our study can guarantee that there is no bias underlying the data. However, there is another fundamental question: can the data reflect the disease accurately? In our study, this question is equivalent to the question that although different procedures were used to produce the two sets of microarrays, can this difference influence the final result at the mRNA level? It is possible that noise or even contaminations could have been introduced into the data between the time the biopsy samples were taken to produce microarrays and the final result we obtained from analyzing microarrays. As a consequence, it is possible that different procedures may lead to the same result and if this happens, it will undermine the results we obtained from different microarrays. However, from **Table 1** **,** **2** **and** **3** we can see that the expression profiles of DEGs related to alcohol metabolism, lipid metabolism and hemoglobin between the two microarrays can reflect the different patient selection methods used to produce these microarrays. In microarray two, to mainly focus on hemoglobin, the age of patients was strictly controlled. On the contrary, age was not controlled in microarray one. Due to this difference, expression profile of genes related to age like hemoglobin genes (*hemoglobin alpha* and *hemoglobin beta*) will be influenced but expression profile of genes which are not associated with age like genes involved in alcohol and lipid metabolism will be unchanged between the two microarrays. The results in **Table 1** **,** **2** **and** **3** reflect this difference. The expression profiles of major genes in alcohol and lipid metabolism between two microarrays were nearly identical but the expression profiles of *hemoglobin alpha* and *hemoglobin beta* were different. In conclusion, the two microarrays used in our study can describe different aspects of NASH accurately and using these two microarrays to investigate the pathogenesis of NASH can give us a more comprehensive understanding of the disease.

### *Alcohol dehydrogenase 4* is a potential biomarker for NASH

Although NASH is a condition of hepatitis irrelevant to alcohol consumption, it shares many histological features with alcoholic liver disease (ALD) such as pericellular fibrosis and macrovesicular and microvesicular fat in hepatocytes [21]. The histology cannot distinguish non-alcoholic patients from alcoholic patients. This sheds light on the assumption that a shared condition may be responsible for both alcoholic and non-alcoholic liver disease (NALD) [47]. Previous studies have confirmed this assumption by proving that alcohol produced by intestinal bacteria [48], [49], [50] and alcohol from diet [51] are involved in NALD like NASH so that alcohol metabolism is not only involved in ALD but also related to NALD. The role of alcohol metabolism in NASH has been investigated in our previous study [23] and genes responsible for alcohol metabolism, especially genes encoding enzymes in alcohol dehydrogenase (ADH) family, showed a high expression in NASH patients. The significant up-regulation of genes related to alcohol metabolism found in this study (see **Tables S11, S12, S13, and S14**) is consistent with the results in these previous studies [23], [48], [49], [50], [51] which validates the significant up-regulation of alcohol metabolism and indicates its importance in the pathogenesis of NASH.

**Figure 3:**
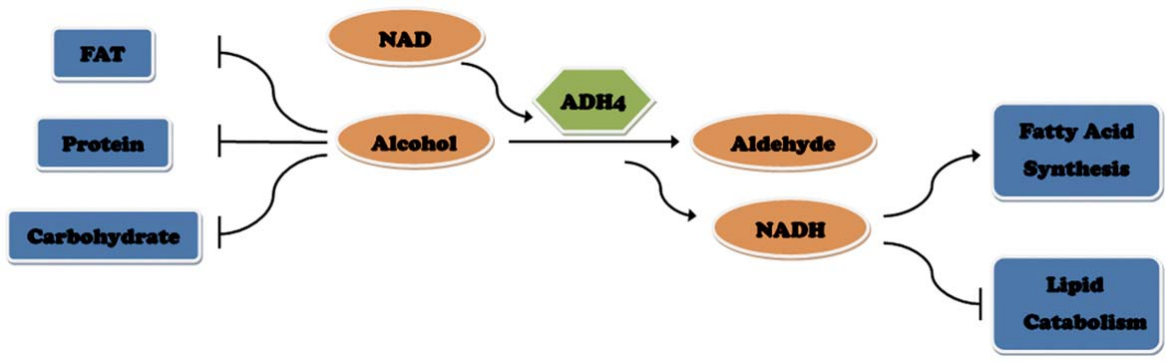
Main reaction of ADH4 and the influence on other pathways. ADH4 is a member of alcohol dehydrogenase enzymes which catalyzes the oxidation of primary and secondary alcohols to aldehydes and ketones, respectively, and reduces NAD to NADH. NADH is the product of this reaction and excess NADH will promote fatty acid synthesis and act against lipid catabolism. Alcohol can injure the liver by blocking the normal metabolism of protein, fats, and carbohydrates. Arrows with a vertical line at the end indicate inhibition. Fat, protein and carbohydrate stand for fat metabolism, protein metabolism and carbohydrate metabolism respectively. Squares in blue represent pathways and ovals represent compounds involved in the reaction catalyzed by ADH4. ADH4 is highlighted in a green hexagon.

In alcohol metabolism, ADH plays an important role. ADH is a group of alcohol dehydrogenase enzymes that catalyze the oxidation of primary and secondary alcohols to aldehydes and ketones, respectively [52], and reduce nicotinamide adenine dinucleotide (NAD) to NADH. One of the evolutionary purposes of ADH is to breakdown alcohols contained in food and produced by intestinal bacteria [53]. There are four major classes of ADH. Most members of ADH family are present in liver; ADH4 is the major hepatic ADH. ADH4 has the same function as other alcohol dehydrogenases: to oxidize ethanol to aldehydes and ketones and to reduce NAD to NADH. It has been reported that the increased level of NADH promotes fatty acid synthesis and acts against lipid catabolism, contributing to fat accumulation in liver [54], [55]. Alcohol may also injure the liver by blocking the normal metabolism of protein, fats, and carbohydrates. **Figure 3** summarizes the main reaction of ADH4 and shows the influence on other pathways. Most importantly, besides the association with several pathways related to the pathogenesis of NASH, the elevated transcription activity of *ADH4* has been validated not only in our study, but also by quantitative real-time polymerase chain reaction (qRT-PCR) at mRNA level and Western blot at protein level in our previous study of investigating the expression profile of alcohol metabolism related genes [23] although *ADH4* was not suggested to be a biomarker in that study. In conclusion, the validated up-regulation of *ADH4* in NASH patients compared with normal controls and the correlation with the pathogenesis of NASH indicate that *ADH4* is a potential biomarker for NASH.

However, since alcohol metabolism is also involved in ALD and some studies have shown that ADH is associated with ALD [56], [57], *ADH4* alone may not be capable of distinguishing ALD from NALD. As a consequence, *ADH4* can be used as a major indicator of NASH, but other features should be used to help distinguish NASH from ALD. For example, alcohol dependence (AD) which almost all patients with ALD have is a key difference between ALD and NALD [58], [59]. In the diagnosis of NASH, the history of alcohol consumption can be used to determine whether the patient has ALD or not. Moreover, nutritional status is another prominent difference. Body Mass Index (BMI) and serum levels of total cholesterol and cholinesterase are all higher in NASH than ALD patients suggesting nutritional status contributes to the assessment [60]. In summary, along with other features used to help distinguish NASH from ALD, *ADH4* is a suitable indicator and biomarker for NASH.

Besides *ADH4*, gene encoding fibronectin type III domain containing 5 (FNDC5) protein also had very high rank (3^rd^ in microarray one and 10^th^ in microarray two) after rank aggregation. Moreover, *FNDC5* was listed in both clusters of genes and had the highest rank compared with other genes in the two clusters. Therefore, the role of FNDC5 in NASH is worth further study.

The *FNDC5* gene encodes a type I membrane protein. Bostrom *et al*. [61] reported that FNDC5 contributed to the improvement of obesity and glucose homeostasis through irisin, a cleaved and secreted fragment of FNDC5. Irisin is responsible for the induction of the browning of subcutaneous fat. The brown fat is then burned as heat. The increased formation of brown fat has been shown to have anti-obesity and anti-diabetic effects. It was also proved that only moderate increase of circulating levels of irisin can potently increase energy expenditure; reduce body weight and diet-induced insulin resistance. Since NASH is strongly associated with obesity and insulin resistance, increasing the amount of circulating irisin may be a good strategy for NASH patients to lose weight and reduce insulin resistance.

### Genes and proteins responsible for amino acid catabolism and downstream metabolisms

In functional analysis, half of the genes in the two clusters were found enriched in pathways related to amino acid catabolism indicating the importance of amino acid catabolism in NASH. Other NASH-associated pathways were also found related to amino acid catabolism, which will be discussed later. This result is the first evidence suggesting that amino acid catabolism plays an important role in the pathogenesis of NASH. Besides, the downstream metabolism of amino acid catabolism, the urea cycle, was also found for the first time to be associated with NASH.

Protein degradation pathways provide substrates for amino acid catabolism. Protein degradation is a very important process in our body. First, protein degradation can wipe out the abnormal proteins to protect cells from being harmed. Second, degradation of excessive enzymes and regulatory proteins can help keep the coordination of metabolism in cells. In eukaryotes, the degradation of proteins requires two mechanisms: the lysosomal mechanism and the ATP-dependent ubiquitin-mediated mechanism.

Besides being the basic unit of proteins, amino acids have many other functions. For example, they are involved in the energy production process and are precursors of important nitrogen-containing compounds. Moreover, excessive amino acids can be transformed into many intermediates like pyruvic acid, oxaloacetic acid and alpha-keto acid. Therefore, the catabolism of amino acids has a wide ranging influence on many pathways.

The first step of amino acid catabolism is deamination and this can be achieved by transamination, oxidative deamination, transdeamination and other deamination reactions. Transdeamination is an important way since transamination alone cannot guarantee a thorough deamination. There are two reactions called transdeamination. In one of them, aspartate is produced by the reaction between glutamate and oxaloacetate under the catalysis of aspartate aminotransferase (AST). Although *AST* is not in the two clusters of DEGs, its expression was significantly up-regulated, which indicated the up-regulation of transdeamination. Apart from transdeamination, there are other deamination reactions. DAO is involved in non selective deamination. It is a non specific amino acid oxidase which is a flavoprotein and uses flavin adenine dinucleotide (FAD) as its prosthetic group. It catalyzes the transformation of amino acid into alpha-keto acid. The up-regulation of *DAO* in our study indicates that the DAO-induced non selective deamination was also up-regulated.

Interestingly, catabolism of several particular amino acids was up-regulated. PECR is involved in tyrosine metabolism. PIPOX and DAO are involved in glycine, serine, threonine metabolism. PIPOX and EHHADH play a major role in lysine degradation. EHHADH is also involved in valine, leucine, isoleucine degradation and beta-alanine metabolism. In tryptophan metabolism, CAT and EHHADH are involved. The products of amino acid catabolism are free ammonia and carbon skeletons of these amino acids. The carbon skeletons can be transformed into other metabolites like acetyl-CoA and pyruvic acid which will influence other metabolisms like fatty acid metabolism and carbohydrate metabolism. Free ammonia is harmful to our body especially the brain. Just 1% ammonia in our blood can lead to the intoxication of the central nervous system. As a result, the secretion of ammonia is important. In most cases, free ammonia enters the urea cycle and is removed from the body as urea. CAT is involved in this process. Under the catalysis of CAT, glutamic acid interacts with N2-acetyl-L-ornithine and generates ornithine and N-aceyl-L-glutamate.

In conclusion, the genes in the two clusters are highly enriched in amino acid catabolism. These genes are all up-regulated, indicating the up-regulation of amino acid catabolism in NASH livers. In addition, the products of amino acid catabolism such like acetyl-CoA and pyruvic acid are precursors of many other NASH related pathways. The connection between amino acid catabolism and oxidative stress found in our study provides direct corroboration of previous studies [62], [63]. The relationship between amino acid catabolism and lipid metabolism is validated by [64], [65], and the interplay between amino acid catabolism and gluconeogenesis have already been reported [65], [66], [67], [68]. All these facts lend credibility to the conclusion that the elevated amino acid catabolism plays a pivotal role in the pathogenesis of NASH.

### Genes and proteins responsible for lipid metabolism

According to the “two-hits” theory, the accumulation of fat in liver is the prerequisite for NASH. The result of this study is consistent with our previous work [25] which examined the molecular etiology of the liver fat accumulation in NASH. Moreover, the current study supports the previous result from another perspective by showing the up-regulation of *PECR*, *EHHADH*, *ECI2* and *PHYH*. PECR and EHHADH are involved in fatty acid elongation in mitochondria and the production of acetyl-CoA from amino acid catabolism, which provides precursors for fatty acid synthesis. Additionally, fatty acid synthase (FASN) and CD36 which are two very important proteins in de novo synthesis and fatty acid uptake are regulated by EHHADH. The elevated expression of *EHHADH* and *PECR* indicated that the lipogenesis in hepatocyte was up-regulated. However, EHHADH is also one of the four enzymes of the peroxisomal beta-oxidation pathway. And our study shows that both beta-oxidation and alpha-oxidation were up-regulated. ECI2 is a key mitochondrial enzyme involved in beta-oxidation of unsaturated fatty acids. It catalyzes the transformation of 3-cis and 3-trans-enoyl-CoA esters arising during the stepwise degradation of cis-, mono-, and polyunsaturated fatty acids to the 2-trans-enoyl-CoA intermediates. *PHYH* encodes a peroxisomal protein that is involved in the alpha-oxidation of 3-methyl branched fatty acids. Specifically, this protein converts phytanoyl-CoA to 2-hydroxyphytanoyl-CoA. Therefore, the oxidation of fatty acid was increased instead of being decreased.

### Genes and proteins responsible for other metabolisms

#### Gluconeogenesis

Gluconeogenesis is a major part of carbohydrate metabolism that maintains a constant supply of glucose for the brain, kidney, testes and red blood cells. We found that gluconeogenesis is associated with NASH. As shown in **Figure 2** **,** metabolism of glycine, serine, threonine and beta-alanine generating pyruvic acid, a non sugar precursor for gluconeogenesis, are up-regulated. Consequently, it is likely that the up-regulated amino acid catabolism we describe above influences gluconeogenesis by providing more precursors so gluconeogenesis is up-regulated in NASH patients. In addition, Sunny *et al*. [69] found that people with excessive fat accumulation exhibit mitochondrial anaplerosis which provides substrates for gluconeogenesis and the induction of lipid oxidation is required for gluconeogenesis. Since we have shown that lipid oxidation is up-regulated, it is very likely that gluconeogenesis is also up-regulated and associated with fat accumulation in NASH patients.

Together with the fact that acetyl-CoA generated by oxygenolysis of carbohydrate also leads to lipogenesis, our data suggested that abnormal carbohydrate metabolism, especially gluconeogenesis, is strongly associated with NASH.

#### Oxidative stress response

Oxidative stress is thought to be important for the progression from steatosis alone to NASH and finally to cirrhosis [19], [24], [70], [71]. It is caused by an imbalance between the production of reactive oxygen and the detoxification of reactive intermediates. Reactive intermediates such as peroxides and free radicals can be harmful to many parts of cells such as proteins, lipids and DNA. Severe oxidative stress can lead to apoptosis and necrosis. The result of our study is in accordance with the relationship between oxidative stress and NASH.

When reactive oxygen species are produced, the oxidative stress response is triggered. The proteins encoded by genes in the two clusters and involved in oxidative stress response are CAT and PXMP2. PXMP2 is a peroxisomal membrane protein. Peroxisomes play a pivotal role in detoxification, fatty acid oxidation and regulation of oxygen. For CAT, it is a key antioxidant enzyme in the body's defense against oxidative stress. CAT is a heme enzyme that is present in peroxisomes. CAT converts the reactive oxygen species hydrogen peroxide to water and oxygen and thereby mitigates the toxic effects of hydrogen peroxide so that it helps reduce the oxidative stress. CAT is also involved in NRF2-mediated oxidative stress response. The elevated expression of genes encoding PXMP2 and CAT indicate the mounting need to deal with reactive oxygen species like hydrogen peroxide.

Although DAO is not involved in the oxidative stress response, it is responsible for the production of reactive intermediates. DAO catalyzes nonspecific deamination, arginine and proline metabolism. During these reactions, hydrogen peroxide is generated. Since the gene encoding DAO is up-regulated, the production of hydrogen peroxide is increased in NASH patients. This over-production of hydrogen peroxide along with the up-regulation of genes responsible for oxidative stress response are consistent with previous studies and support the important role of oxidative stress in the pathogenesis of NASH.

### The molecular pathological mechanism and the interplay between different pathways

The network in **Figure 2** summarizes the main pathways in the pathogenesis of NASH that this approach identifies. In this network, proteins are degraded into amino acids through a lysosomal mechanism and an ATP-dependent mechanism. The amino acids are then decomposed through different amino acid catabolic pathways. The elevated activity of amino acid catabolism is an important difference between normal controls and NASH patients. It also connects many other important pathways related to the pathogenesis of NASH. The main products of amino acid catabolism are pyruvic acid, free ammonia, hydrogen peroxide and acetyl-CoA. The over production of these key metabolites caused by the elevated activity of amino acid catabolism influences downstream pathways and this is consistent with the up-regulation of these downstream pathways found independently in our study.

First, amino acid catabolism influences fatty acid synthesis through acetyl-CoA. Since acetyl-CoA is the precursor for fatty acid synthesis, its production accelerates this process. In addition, ACOT2 is regulated by EHHADH in fatty acid beta-oxidation and it can then regulate acetyl-CoA which in turn, influences the fatty acid synthesis. The interaction among ACOT2, EHHADH and acetyl-CoA forms a cycle connecting fatty acid synthesis and oxidation. Together with our previous work, we found that the up-regulation of fatty acid synthesis overcomes the elevated fatty acid oxidation and very low-density lipoprotein (VLDL) secretion and contributes to the accumulation of fat in liver and the development of NASH.

Second, excessive hydrogen peroxides produced through amino acid catabolism stimulates the oxidative stress response. The up-regulated oxidative stress response found in our study shows that the liver failed to effectively reduce increased amounts of reactive oxygen species like hydrogen peroxides. This leads to oxidative stress and then triggers the progression from steatosis alone to NASH.

Third, excessive free ammonia produced through amino acid catabolism enters the urea cycle where urea is produced for excretion. The elevated activity of the urea cycle found in our study proved this link and in turn, validated the up-regulation of amino acid catabolism.

Fourth, the increased amount of pyruvic acid provides precursors for gluconeogenesis. Although no direct evidence showing the up-regulation of gluconeogenesis was found in our study, the increased amount of pyruvic acid may be a hint that this process is accelerated. In addition, the possible relationship between elevated gluconeogenesis and fat accumulation indicated in a previous study [69] lends credibility to the conclusion that the up-regulation of gluconeogenesis is very likely to be involved in the pathogenesis of NASH. However, the exact role of gluconeogenesis in the development of NASH requires further study.

All pathways in the network not only agree with each other but also agree with previous studies,which lend credibility to the validity of the molecular pathological mechanism of NASH.

## Conclusion

Our study analyzed the whole genome expression profile between NASH patients and normal controls and constructed the network of NASH related pathways. Results reported in [23], [25], [28] which were used in the filtration of four DEG identifying methods were the only reported NASH related experimental data used during the statistical analyses of our study. Results reported in other previous studies were used as cross validation after we obtained the results based on statistical analyses. Our findings not only agree with previous studies but also provide a new possible mechanism to the pathogenesis of NASH. While these new findings in the molecular pathology of NASH warrants further experimental validation, the information we obtained from this study can help us understand the interplay between different pathways and the molecular pathological mechanism of NASH from a more systematic perspective. Our data suggested that *ADH4* is a potential biomarker for NASH. Functional analysis performed with the intersection of DEGs provided the first evidence suggesting that elevated amino acid catabolism plays a central role in the pathogenesis of NASH. Gluconeogenesis, urea cycle, lipid metabolism and oxidative stress response were also found associated with NASH. Our study provides a more comprehensive understanding of the biomarker and molecular pathological mechanisms underlying the development of NASH and this may facilitate the diagnosis, prevention and treatment of NASH.

## Supporting Information

Figure S1 **The original network of** **figure 2** **with reactions on it.**
(PDF)

Figure S2 **The interaction network of genes in the two clusters and proteins encoded by these genes before being classified into pathways.** This is the interaction network of genes in two clusters and proteins encoded by these genes. Since there are some indirect connections, we add the intermediate genes, proteins or microRNAs into the network. Genes in the two clusters and proteins they encode are represented by dark hexagons. Intermediate genes and proteins are represented by white ovals. Intermediate microRNAs are represented by white squares. Solid lines indicate direct connections and dashed lines indicate indirect connections.
(TIF)

Table S1 **Down-regulated DEGs identified by SAM in microarray one.**
(XLS)

Table S2 **Up-regulated DEGs identified by SAM in microarray one.**
(XLS)

Table S3 **DEGs identified by** ***t*** **-test in microarray one under the cutoff of p-value = 0.05.**
(XLS)

Table S4 **DEGs identified by WAD in microarray one under the cutoff of WAD = 1.5.**
(XLS)

Table S5 **DEGs identified by Wilcoxon rank sum test in microarray one under the cutoff of p-value = 0.05.**
(XLS)

Table S6 **Down-regulated DEGs identified by SAM in microarray two.**
(XLS)

Table S7 **Up-regulated DEGs identified by SAM in microarray two.**
(XLS)

Table S8 **DEGs identified by** ***t*** **-test in microarray two under the cutoff of p-value = 0.05.**
(XLS)

Table S9 **DEGs identified by WAD in microarray two under the cutoff of WAD = 1.5.**
(XLS)

Table S10 **DEGs identified by Wilcoxon rank sum test in microarray two under the cutoff of p-value = 0.05.**
(XLS)

Table S11 **Detailed information about DEGs related to alcohol metabolism found by SAM.**
(DOC)

Table S12 **Detailed information about DEGs related to alcohol metabolism found by** ***t*** **-test.**
(DOC)

Table S13 **Detailed information about DEGs related to alcohol metabolism found by WAD.**
(DOC)

Table S14 **Detailed information about DEGs related to alcohol metabolism found by Wilcoxon rank sum test.**
(DOC)

Table S15 **Detailed information about genes related to hemoglobin found by four methods.**
(DOC)

Table S16 **Detailed information about DEGs related to lipid metabolism found by** ***t*** **-test.**
(DOC)

Table S17 **Detailed information about DEGs related to lipid metabolism found by WAD.**
(DOC)

Table S18 **Detailed information about DEGs related to lipid metabolism found by Wilcoxon rank sum test.**
(DOC)

Table S19 **DEGs identified by Wilcoxon rank sum test in microarray one under the cutoff of p-value = 0.01.**
(XLS)

Table S20 **DEGs identified by** ***t*** **-test in microarray one under the cutoff of p-value = 0.01.**
(XLS)

Table S21 **DEGs identified by Wilcoxon rank sum test in microarray two under the cutoff of p-value = 0.01.**
(XLS)

Table S22 **DEGs identified by** ***t*** **-test in microarray two under the cutoff of p-value = 0.01.**
(XLS)

Table S23 **DAVID functional annotation clustering result of the intersection of DEGs indentified by three methods in microarray one.**
(XLS)

Table S24 **DAVID functional annotation clustering result of the intersection of DEGs indentified by three methods in microarray two.**
(XLS)

Table S25 **DAVID functional annotation clustering result of DEGs identified by** ***t*** **-test in microarray one.**
(XLS)

Table S26 **DAVID functional annotation clustering result of DEGs identified by WAD in microarray one.**
(XLS)

Table S27 **DAVID functional annotation clustering result of DEGs identified by Wilcoxon rank sum test in microarray one.**
(XLS)

Table S28 **DAVID functional annotation clustering result of DEGs identified by** ***t*** **-test in microarray two.**
(XLS)

Table S29 **DAVID functional annotation clustering result of DEGs identified by WAD in microarray two.**
(XLS)

Table S30 **DAVID functional annotation chart result of DEGs identified by Wilcoxon rank sum test in microarray two.**
(XLS)

Table S31 **The cross reference list of numbers in** **figure 2** **and their corresponding reaction equations.**
(DOC)

Table S32 **Rank aggregation result of microarray one.**
(XLS)

Table S33 **Rank aggregation result of microarray two.**
(XLS)

Table S34 **Information of top five ESTs after rank aggregation in microarray one.**
(DOC)

Table S35 **Information of top five ESTs after rank aggregation in microarray two.**
(DOC)
