# Supplementary figures and images for: Systematic Analysis of the Gene Expression in the Livers of Nonalcoholic Steatohepatitis: Implications on Potential Biomarkers and Molecular Pathological Mechanism
Source: PLoS One. 2012 Dec 26;7(12):e51131. doi: 10.1371/journal.pone.0051131 (PMC3530598; doi:10.1371/journal.pone.0051131)

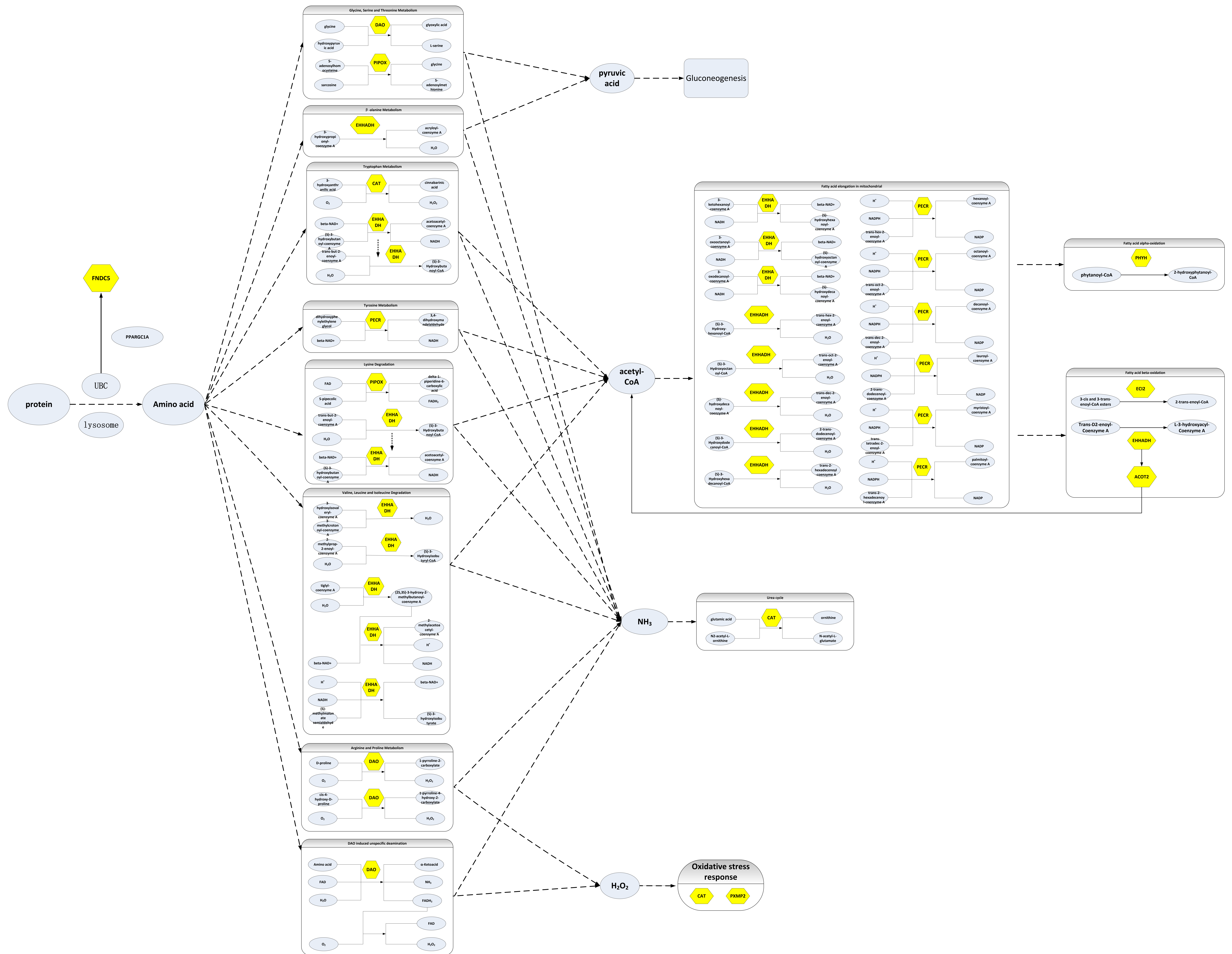

Supplement: Figure S1 — The original network of figure 2 with reactions on it. (PDF) [file pone.0051131.s001.pdf]

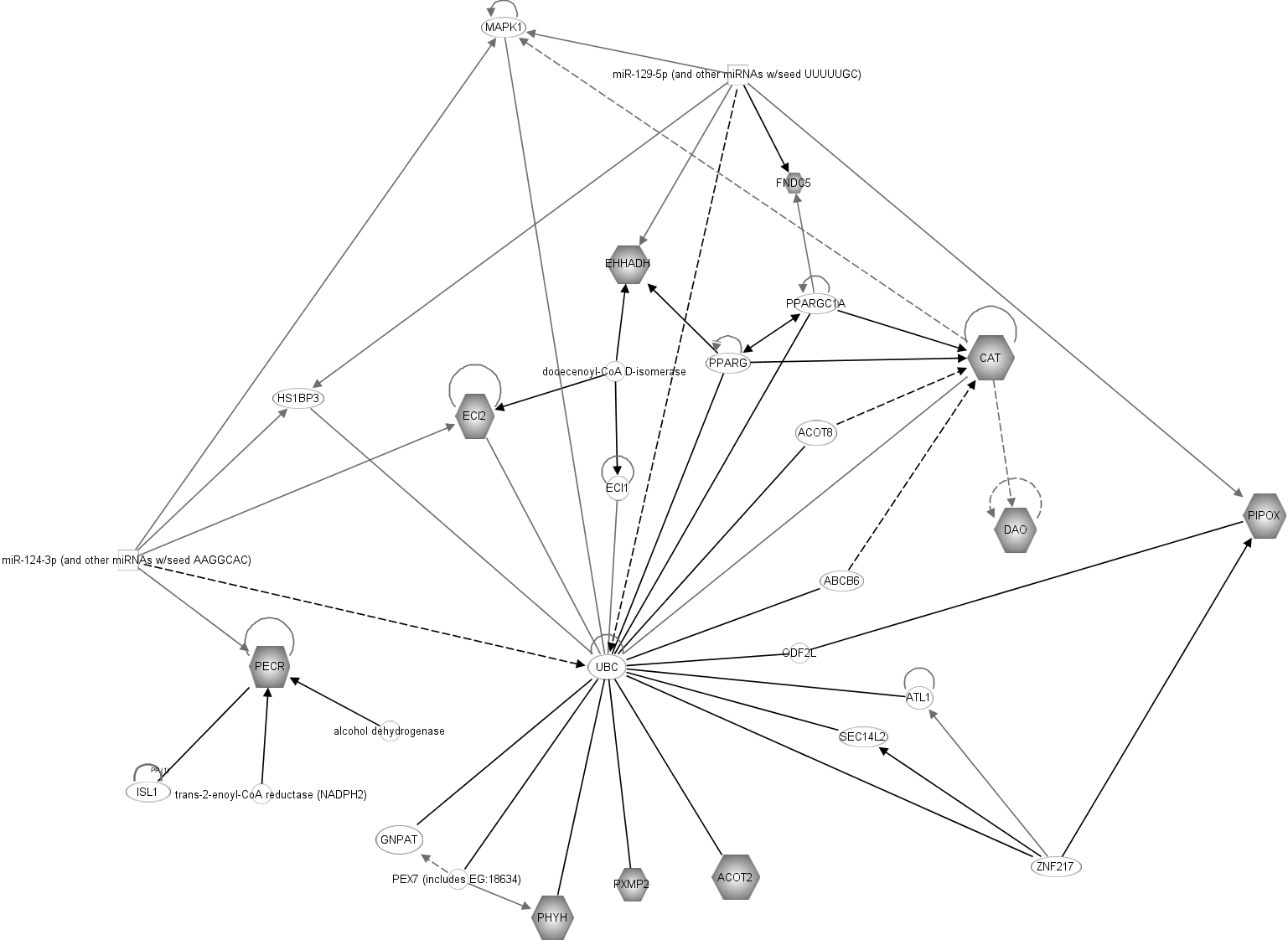

Supplement: Figure S2 — The interaction network of genes in the two clusters and proteins encoded by these genes before being classified into pathways. This is the interaction network of genes in two clusters and proteins encoded by these genes. Since there are some indirect connections, we add the intermediate genes, proteins or microRNAs into the network. Genes in the two clusters and proteins they encode are represented by dark hexagons. Intermediate genes and proteins are represented by white ovals. Intermediate microRNAs are represented by white squares. Solid lines indicate direct connections and dashed lines indicate indirect connections. (TIF) [file pone.0051131.s002.tif]
