# Supplementary material for: Systematic Analysis of the Gene Expression in the Livers of Nonalcoholic Steatohepatitis: Implications on Potential Biomarkers and Molecular Pathological Mechanism
Source: PLoS One. 2012 Dec 26;7(12):e51131. doi: 10.1371/journal.pone.0051131 (PMC3530598; doi:10.1371/journal.pone.0051131)
Supplement: Table S11 — Detailed information about DEGs related to alcohol metabolism found by SAM. (DOC) [file pone.0051131.s013.doc]

**SAM^1^:**

| Microarray one | | |  | Microarray two | | |
| --- | --- | --- | --- | --- | --- | --- |
| GenBank  Accession | Gene  Name | Score(d) |  | GenBank  Accession | Gene  Name | Score(d) |
| NM_000670.2 | alcohol dehydrogenase 4 (class II),pi polypeptide (ADH4) | 6.4798 |  | NM_000670.2 | alcohol dehydrogenase 4 (class II),pi polypeptide (ADH4) | 4.4818 |
| NM_000672.2 | alcohol dehydrogenase 6 (class V) (ADH6) | 4.6447 |  | NM_000672.2 | alcohol dehydrogenase 6 (class V) (ADH6) | 3.2267 |
| NM_022568.2 | aldehyde dehydrogenase 8 family,member A1 (ALDH8A1) | 4.5335 |  |  |  |  |

^1^: SAM stands for Significance Analysis of Microarrays
