# Supplementary material for: Systematic Analysis of the Gene Expression in the Livers of Nonalcoholic Steatohepatitis: Implications on Potential Biomarkers and Molecular Pathological Mechanism
Source: PLoS One. 2012 Dec 26;7(12):e51131. doi: 10.1371/journal.pone.0051131 (PMC3530598; doi:10.1371/journal.pone.0051131)
Supplement: Table S12 — Detailed information about DEGs related to alcohol metabolism found by t -test. (DOC) [file pone.0051131.s014.doc]

***t*-test：**

| Microarray one | | |  | Microarray two | | |
| --- | --- | --- | --- | --- | --- | --- |
| GenBank  Accession | Gene  Name | P-value |  | GenBank  Accession | Gene  Name | P-value |
| NM_000670.2 | alcohol dehydrogenase 4 (class II),pi polypeptide (ADH4) | 0.0001 |  | NM_000670.2 | alcohol dehydrogenase 4 (class II),pi polypeptide (ADH4) | 0.0003 |
| NM_000667.2 | alcohol dehydrogenase 1A (class I),alpha polypeptide (ADH1A) | 0.0198 |  | NM_000667.2 | alcohol dehydrogenase 1A (class I),alpha polypeptide (ADH1A) | 0.0047 |
| NM_000668.3 | alcohol dehydrogenase IB (class I),beta polypeptide (ADH1B) | 0.0249 |  | NM_000668.3 | alcohol dehydrogenase IB (class I),beta polypeptide (ADH1B) | 0.0065 |
| NM_000669.2 | alcohol dehydrogenase 1C (class I),gamma polypeptide (ADH1C) | 0.0076 |  | NM_000669.2 | alcohol dehydrogenase 1C (class I),gamma polypeptide (ADH1C) | 0.0332 |
| NM_000671.2 | alcohol dehydrogenase 5 (class III),chi polypeptide (ADH5) | 0.0167 |  | NM_000671.2 | alcohol dehydrogenase 5 (class III),chi polypeptide (ADH5) | 0.0013 |
| NM_000672.2 | alcohol dehydrogenase 6 (class V) (ADH6) | 0.0011 |  | NM_000672.2 | alcohol dehydrogenase 6 (class V) (ADH6) | 0.0000 |
| NM_001752.1 | catalase (CAT) | 0.0008 |  | NM_001752.1 | catalase (CAT) | 0.0002 |
| NM_000689.3 | aldehyde dehydrogenase 1 family,member A1 (ALDH1A1) | 0.0053 |  | NM_000689.3 | aldehyde dehydrogenase 1 family,member A1 (ALDH1A1) | 0.0010 |
| NM_170697.1 | aldehyde dehydrogenase 1 family,member A2 (ALDH1A2) | 0.0458 |  | NM_000690.2 | aldehyde dehydrogenase 2 family(mitochondrial) (ALDH2) | 0.0012 |
| NM_000690.2 | aldehyde dehydrogenase 2 family(mitochondrial) (ALDH2) | 0.0022 |  | NM_001080.3 | aldehyde dehydrogenase 5 family,member A1 (ALDH5A1) | 0.0134 |
| NM_001080.3 | aldehyde dehydrogenase 5 family,member A1 (ALDH5A1) | 0.0132 |  | NM_001182.1 | aldehyde dehydrogenase ALDH7 | 0.0001 |
| NM_001182.1 | aldehyde dehydrogenase ALDH7 | 0.0003 |  | NM_022568.2 | aldehyde dehydrogenase 8 family,member A1 (ALDH8A1) | 0.0024 |
| NM_022568.2 | aldehyde dehydrogenase 8 family,member A1 (ALDH8A1) | 0.0000 |  |  |  |  |
