# Supplementary material for: Systematic Analysis of the Gene Expression in the Livers of Nonalcoholic Steatohepatitis: Implications on Potential Biomarkers and Molecular Pathological Mechanism
Source: PLoS One. 2012 Dec 26;7(12):e51131. doi: 10.1371/journal.pone.0051131 (PMC3530598; doi:10.1371/journal.pone.0051131)
Supplement: Table S13 — Detailed information about DEGs related to alcohol metabolism found by WAD. (DOC) [file pone.0051131.s015.doc]

**WAD^1^：**

| Microarray one | | |  | Microarray two | | |
| --- | --- | --- | --- | --- | --- | --- |
| GenBank  Accession | Gene  Name | WAD |  | GenBank  Accession | Gene  Name | WAD |
| NM_000670.2 | alcohol dehydrogenase 4 (class II),pi polypeptide(ADH4) | 4.0183 |  | NM_000670.2 | alcohol dehydrogenase 4 (class II),pi polypeptide (ADH4) | 3.6679 |
| NM_000667.2 | alcohol dehydrogenase 1A (class I),alpha polypeptide(ADH1A) | 2.5933 |  | NM_000667.2 | alcohol dehydrogenase 1A (class I),alpha polypeptide (ADH1A) | 2.5771 |
| NM_000672.2 | alcohol dehydrogenase 6 (class V)( ADH6) | 2.0851 |  | NM_000672.2 | alcohol dehydrogenase 6 (class V) (ADH6) | 1.9539 |
| NM_001752.1 | catalase (CAT) | 1.6634 |  | NM_000689.3 | aldehyde dehydrogenase 1 family,member A1 (ALDH1A1) | 1.8724 |
| NM_000689.3 | aldehyde dehydrogenase 1 family,member A1(ALDH1A1) | 2.0696 |  |  |  |  |
| NM_000669.2 | alcohol dehydrogenase 1C (class I),gamma polypeptide(ADH1C) | 1.6080 |  |  |  |  |

^1^:WAD stands for weighted average difference
