# Supplementary material for: Systematic Analysis of the Gene Expression in the Livers of Nonalcoholic Steatohepatitis: Implications on Potential Biomarkers and Molecular Pathological Mechanism
Source: PLoS One. 2012 Dec 26;7(12):e51131. doi: 10.1371/journal.pone.0051131 (PMC3530598; doi:10.1371/journal.pone.0051131)
Supplement: Table S15 — Detailed information about genes related to hemoglobin found by four methods. (DOC) [file pone.0051131.s017.doc]

| Method | GenBank Accession | Gene  Name | Value^1^ in microarray one | Value in microarray two |
| --- | --- | --- | --- | --- |
| WAD^2^ | NM_000518.4 | hemoglobin, beta (HBB)^3^ | 2.0977^4^ | 2.9663 |
|  | NM_000558.3 | hemoglobin, alpha 1 (HBA1) | 1.3870 | 2.2547 |
| Wilcoxon rank-sum test | NM_000518.4 | hemoglobin, beta (HBB) | 0.1152 | 0.0047 |
|  | NM_000558.3 | hemoglobin, alpha 1 (HBA1) | 0.1152 | 0.0031 |
| t-test | NM_000518.4 | hemoglobin, beta (HBB) | 0.0955 | 0.0092 |
|  | NM_000558.3 | hemoglobin, alpha 1 (HBA1) | 0.1335 | 0.0078 |
| SAM^5^ | NM_000518.4 | hemoglobin, beta (HBB) | 1.7646 | 2.8853 |
|  | NM_000558.3 | hemoglobin, alpha 1 (HBA1) | 1.2695 | 2.3829 |

^1^:For WAD, the value is the weighted average difference, for Wilcoxon rank-sum test and *t*-test, the value is the p-value, for SAM, the value is the score(d).

^2^:WAD is short for weighted average difference method

^3^:Hemoglobin alpha and beta are two genes related to hemoglobin. Since there are only two genes, we list all the values calculated by four methods in two microarrays instead of only listing values of genes which are significantly differentially expressed.

^4^: value in red means the corresponding gene is significantly differentially expressed

^5^: SAM stands for Significance Analysis of Microarrays
