# Supplementary material for: Systematic Analysis of the Gene Expression in the Livers of Nonalcoholic Steatohepatitis: Implications on Potential Biomarkers and Molecular Pathological Mechanism
Source: PLoS One. 2012 Dec 26;7(12):e51131. doi: 10.1371/journal.pone.0051131 (PMC3530598; doi:10.1371/journal.pone.0051131)
Supplement: Table S16 — Detailed information about DEGs related to lipid metabolism found by t -test. (DOC) [file pone.0051131.s018.doc]

***t*-test：**

| Microarray one | | |  | Microarray two | | |
| --- | --- | --- | --- | --- | --- | --- |
| GenBank  Accession | Gene  Name | P-value |  | GenBank  Accession | Gene  Name | P-value |
| NM_002080. 1 | FABPpm; mitochondrial aspartate aminotransferase | 0.0065 |  | NM_002080. 1 | FABPpm; mitochondrial aspartate aminotransferase | 0.0057 |
| NM_003645. 2 | Solute carrier family 27 (fatty acid transporter),member 2 | 0.0211 |  | NM_001001547.1 | CD36 antigen | 0.0059 |
| NM_005063. 3 | Stearoyl-CoA desaturase 1 (SCD1) | 0.0017 |  | NM_005063. 3 | Stearoyl-CoA desaturase 1 (SCD1) | 0.0016 |
| NM_004104.4 | Fatty acid synthase (FASN) | 0.0109 |  | NM_004104.4 | Fatty acid synthase (FASN) | 0.0499 |
| NM_178176. 2 | Acyl-CoA: monoacylglycerol acyltransferase 3 (MGAT3) | 0.0002 |  | NM_178176. 2 | Acyl-CoA: monoacylglycerol acyltransferase 3 (MGAT3) | 0.0004 |
| NM_025098. 2 | Acyl-CoA: monoacylglycerol acyltransferase 2 (MGAT2) | 0.0014 |  | NM_025098. 2 | Acyl-CoA: monoacylglycerol acyltransferase 2 (MGAT2) | 0.0124 |
| NM_001752. 1 | Catalase (CAT), mRNA | 0.0008 |  | NM_001752. 1 | Catalase (CAT), mRNA | 0.0002 |
| NM_006117.2 | Peroxisomal D3,D2-enoyl-CoA isomerase (PECI) | 0.0003 |  | NM_006117.2 | Peroxisomal D3,D2-enoyl-CoA isomerase (PECI) | 0.0001 |
| NM_001966.1 | Enoyl-CoA: hydratase 3-hydroxyacyl–CoA dehydrogenase (EHHADH) | 0.0009 |  | NM_001966. 1 | Enoyl-CoA: hydratase 3-hydroxyacyl–CoA dehydrogenase (EHHADH) | 0.0006 |
| NM_001608.2 | Long chain acyl-CoA dehydrogenase (ACADL) | 0.0001 |  | NM_001608.2 | Long chain acyl-CoA dehydrogenase (ACADL) | 0.0000 |
| NM_000387.3 | Carnitine/acylcarnitine translocase | 0.0022 |  | NM_000483. 3 | Apolipoprotein C-II (APOC2) | 0.0062 |
| NM_001646. 1 | Apolipoprotein C-IV (APOC4) | 0.0131 |  | NM_001646. 1 | Apolipoprotein C-IV (APOC4) | 0.0014 |
| NM_000253. 1 | Microsomal triglyceride transfer protein (MTP) | 0.0123 |  | NM_000253. 1 | Microsomal triglyceride transfer protein (MTP) | 0.0120 |
