# Supplementary material for: Systematic Analysis of the Gene Expression in the Livers of Nonalcoholic Steatohepatitis: Implications on Potential Biomarkers and Molecular Pathological Mechanism
Source: PLoS One. 2012 Dec 26;7(12):e51131. doi: 10.1371/journal.pone.0051131 (PMC3530598; doi:10.1371/journal.pone.0051131)
Supplement: Table S17 — Detailed information about DEGs related to lipid metabolism found by WAD. (DOC) [file pone.0051131.s019.doc]

**WAD^1^：**

| Microarray one | | |  | Microarray two | | |
| --- | --- | --- | --- | --- | --- | --- |
| GenBank  Accession | Gene  Name | WAD |  | GenBank  Accession | Gene  Name | WAD |
| NM_005063. 3 | Stearoyl-CoA desaturase 1 (SCD1) | 1.7079 |  | NM_001966. 1 | Enoyl-CoA: hydratase 3-hydroxyacyl–CoA dehydrogenase (EHHADH) | 1.5792 |
| NM_001752. 1 | Catalase (CAT), mRNA | 1.6634 |  | NM_006117.2 | Peroxisomal D3,D2-enoyl-CoA isomerase (PECI) | 1.6682 |
| NM_006117.2 | Peroxisomal D3,D2-enoyl-CoA isomerase (PECI) | 1.8435 |  | NM_001646. 1 | Apolipoprotein C-IV (APOC4) | 1.5070 |
| NM_001966. 1 | Enoyl-CoA: hydratase 3-hydroxyacyl–CoA dehydrogenase (EHHADH) | 1.8503 |  |  |  |  |
| NM_001646. 1 | Apolipoprotein C-IV (APOC4) | 1.6864 |  |  |  |  |

^1^:WAD stands for weighted average difference
