# Supplementary material for: Systematic Analysis of the Gene Expression in the Livers of Nonalcoholic Steatohepatitis: Implications on Potential Biomarkers and Molecular Pathological Mechanism
Source: PLoS One. 2012 Dec 26;7(12):e51131. doi: 10.1371/journal.pone.0051131 (PMC3530598; doi:10.1371/journal.pone.0051131)
Supplement: Table S31 — The cross reference list of numbers in figure 2 and their corresponding reaction equations. (DOC) [file pone.0051131.s033.doc]

**Glycine, Serine and Threonine Metabolism:**

**DAO:**

| **1** | **Glycine + hydroxypyruvic acid glyoxylic acid + L-serine** |
| --- | --- |

**PIPOX:**

| **2** | **S-adenosylhomocysteine + sarcosine glycine + S-adenosylmethionine** |
| --- | --- |

**β-alanine Metabolism:**

**EHHADH:**

| **3** | **3-hydroxypropionyl-coenzyme A acryloyl-coenzyme A + H_2_O** |
| --- | --- |

**Tryptophan metabolism:**

**CAT:**

| **4** | **3-hydroxyanthranilic acid + O_2_ cinnabarinic acid + H_2_O_2_** |
| --- | --- |

**EHHADH:**

| **5** | **beta-NAD^+^ + (S)-3-hydroxybutanoyl-coenzyme A acetoacetyl-coenzyme A** **+ NADH** |
| --- | --- |
| **6** | **trans-but-2-enoyl-coenzyme A + H_2_O (S)-3-Hydroxybutanoyl-CoA** |

**Tyrosine Metabolism:**

**PECR:**

| **7** | **dihydroxyphenylethylene glycol + beta-NAD^+^ 3,4-dihydroxymandelaldehyde + NADH** |
| --- | --- |

**Lysine Degradation:**

**PIPOX:**

| **8** | **FAD + S-pipecolic acid delta-1-piperidine-6-carboxylic acid + FADH_2_** |
| --- | --- |

**EHHADH:**

| **9** | **trans-but-2-enoyl-coenzyme A + H_2_O (S)-3-Hydroxybutanoyl-CoA** |
| --- | --- |
| **10** | **beta-NAD^+^ + (S)-3-hydroxybutanoyl-coenzyme A acetoacetyl-coenzyme A + NADH** |

**Valine, Leucine and Isoleucine Degradation:**

**EHHADH:**

| 11 | **3-hydroxyisovaleryl-coenzyme A 3-methylcrotonoyl-coenzyme A + H_2_O** |
| --- | --- |
| 12 | **2-methylprop-2-enoyl-coenzyme A** + **H_2_O (S)-3-Hydroxyisobutyryl-CoA** |
| 13 | **tiglyl-coenzyme A + H_2_O (2S,3S)-3-hydroxy-2-methylbutanoyl-coenzyme A** |
| 14 | **(2S,3S)-3-hydroxy-2-methylbutanoyl-coenzyme A + beta-NAD^+^ 2-methylacetoacetyl-coenzyme A** + **H^+^ + NADH** |
| 15 | **H^+^ + NADH + (S)-methylmalonate semialdehyde beta-NAD^+^ + (S)-3-hydroxyisobutyrate** |

**Arginine and Proline Metabolism:**

**DAO:**

| **16** | **D-proline + O_2_ 1-pyrroline-2-carboxylate + H_2_O_2_** |
| --- | --- |
| **17** | **cis-4-hydroxy-D-proline + O_2_ 1-pyrroline-4-hydroxy-2-carboxylate + H_2_O_2_** |

**DAO induced unspecific deamination:**

**DAO:**

| **18** | **Amino acid + FAD + H_2_O α-Ketoacid + NH_3_ + FADH_2_** |
| --- | --- |
| **19** | **FADH_2_ + O_2_ FAD + H_2_O_2_** |

**Fatty acid elongation in mitochondrial:**

**EHHADH:**

| **20** | **3-ketohexanoyl-coenzyme A + NADH beta-NAD^+^ + (S)-hydroxyhexanoyl-coenzyme A** |
| --- | --- |
| **21** | **3-oxooctanoyl-coenzyme A + NADH beta-NAD^+^ + (S)-hydroxyoctanoyl-coenzyme A** |
| **22** | **3-oxodecanoyl-coenzyme A + NADH beta-NAD^+^ + (S)-hydroxydecanoyl-coenzyme A** |
| **23** | **(S)-3-Hydroxy-hexanoyl-CoA trans-hex-2-enoyl-coenzyme A + H_2_O** |
| **24** | **(S)-3-Hydroxyoctanoyl-CoA trans-oct-2-enoyl-coenzyme A + H_2_O** |
| **25** | **(S)-hydroxydecanoyl-coenzyme A trans-dec-2-enoyl-coenzyme A + H_2_O** |
| **26** | **(S)-3-Hydroxydodecanoyl-CoA 2-trans-dodecenoyl-coenzyme A + H_2_O** |
| **27** | **(S)-3-Hydroxyhexadecanoyl-CoA trans-2-hexadecenoyl-coenzyme A + H_2_O** |

**PECR:**

| **28** | **H^+^ + NADPH + trans-hex-2-enoyl-coenzyme A hexanoyl-coenzyme A + NADP** |
| --- | --- |
| **29** | **H^+^ + NADPH + trans-oct-2-enoyl-coenzyme A octanoyl-coenzyme A + NADP** |
| **30** | **H^+^ + NADPH + trans-dec-2-enoyl-coenzyme A decanoyl-coenzyme A + NADP** |
| **31** | **H^+^ + NADPH + 2-trans-dodecenoyl-coenzyme A lauroyl-coenzyme A + NADP** |
| **32** | **H^+^ + NADPH + trans-tetradec-2-enoyl-coenzyme A myristoyl-coenzyme A + NADP** |
| **33** | **H^+^ + NADPH + trans-2-hexadecenoyl-coenzyme A palmitoyl-coenzyme A + NADP** |

**Urea cycle:**

**CAT:**

| **34** | **glutamic acid + N2-acetyl-L-ornithine ornithine + N-acetyl-L-glutamate** |
| --- | --- |

**Fatty acid alpha-oxidation:**

**PHYH:**

| **35** | **phytanoyl-CoA 2-hydroxyphytanoyl-CoA** |
| --- | --- |

**Fatty acid beta-oxidation:**

**ECI2:**

| **36** | **3-cis and 3-trans-enoyl-CoA esters 2-trans-enoyl-CoA** |
| --- | --- |

**EHHADH:**

| **37** | **Trans-D2-enoyl-Coenzyme A L-3-hydroxyacyl-Coenzyme A** |
| --- | --- |
