# Supplementary material for: Systematic Analysis of the Gene Expression in the Livers of Nonalcoholic Steatohepatitis: Implications on Potential Biomarkers and Molecular Pathological Mechanism
Source: PLoS One. 2012 Dec 26;7(12):e51131. doi: 10.1371/journal.pone.0051131 (PMC3530598; doi:10.1371/journal.pone.0051131)
Supplement: Table S34 — Information of top five ESTs after rank aggregation in microarray one. (DOC) [file pone.0051131.s036.doc]

1. **AA551150:**

[Transcribed locus](http://www.ncbi.nlm.nih.gov/UniGene/clust.cgi?UGID=216210&TAXID=9606&SEARCH=AA551150)

Homo sapiens

Hs.401062: 7 sequences.

Transcribed locus

| **GENE EXPRESSION**  Tissues and development stages from this gene's sequences survey gene expression. Links to other NCBI expression resources.   |  | [EST Profile](http://www.ncbi.nlm.nih.gov/UniGene/ESTProfileViewer.cgi?uglist=Hs.401062): | Approximate expression patterns inferred from EST sources. | | --- | --- | --- | |  | [GEO Profiles](http://www.ncbi.nlm.nih.gov/sites/entrez?DB=geoprofiles&DbFrom=unigene&IdsFromResult=216210&cmd=Link&LinkName=unigene_geoprofiles&tool=UniGene.clust): | Experimental gene expression data (Gene Expression Omnibus). | |  | cDNA Sources: | mixed; brain; liver; nerve | |  |  |
| --- | --- | --- | --- | --- | --- | --- | --- | --- | --- | --- | --- |
|  |  |  |

**MAPPING POSITION**

Genomic location specified by transcript mapping, radiation hybrid mapping, genetic mapping or cytogenetic mapping.

|  | Chromosome: | 3 |  | |
| --- | --- | --- | --- | --- |
|  | UniSTS entry: | Chr 3 | [RH35958](http://www.ncbi.nlm.nih.gov/genome/sts/sts.cgi?uid=85618) |  |
|  | UniSTS entry: | Chr 3 | [SHGC-33570](http://www.ncbi.nlm.nih.gov/genome/sts/sts.cgi?uid=82704) | [[Map Viewer](http://www.ncbi.nlm.nih.gov/mapview/maps.cgi?taxid=9606&chr=3&MAPS=ncbirh-r,shgcg3-r,tng-r,g3-r&sts=82704)] |

**SEQUENCES**

*Sequences representing this gene; mRNAs, ESTs, and gene predictions supported by transcribed sequences.*

**EST sequences (7)**

|  | [BX097051.1](http://www.ncbi.nlm.nih.gov/UniGene/seq.cgi?ORG=Hs&SID=11125325) | Clone IMAGp998B05134_;_IMAGE:128932 | mixed |  | **A^1^** |
| --- | --- | --- | --- | --- | --- |
|  | [AW128889.1](http://www.ncbi.nlm.nih.gov/UniGene/seq.cgi?ORG=Hs&SID=1638636) | Clone IMAGE:2615068 | brain | 3' read | **A** |
|  | [DW437728.1](http://www.ncbi.nlm.nih.gov/UniGene/seq.cgi?ORG=Hs&SID=30172735) |  | liver |  |  |
|  | [T84085.1](http://www.ncbi.nlm.nih.gov/UniGene/seq.cgi?ORG=Hs&SID=72198) | Clone IMAGE:111266 | mixed | 3' read |  |
|  | [AA551150.1](http://www.ncbi.nlm.nih.gov/UniGene/seq.cgi?ORG=Hs&SID=805992) | Clone IMAGE:1019405 | nerve | 3' read |  |
|  | [R02497.1](http://www.ncbi.nlm.nih.gov/UniGene/seq.cgi?ORG=Hs&SID=90677) | Clone IMAGE:124640 | mixed | 5' read |  |
|  | [R10691.1](http://www.ncbi.nlm.nih.gov/UniGene/seq.cgi?ORG=Hs&SID=96452) | Clone IMAGE:128932 | mixed | 3' read |  |

^1^: **A** Contains a poly-**A**denylation signal

**P** Has similarity to known **P**roteins (after translation)
**S** Sequence is a **S**uboptimal member of this cluster
**M** Clone is putatively CDS-complete by **M**GC criteria

**EST Profile^2^：**

[Hs.401062](http://www.ncbi.nlm.nih.gov/UniGene/clust.cgi?ORG=Hs&CID=401062) - Transcribed locus

Breakdown by Body Sites

| liver | 4 | 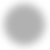 | 1 | / | 205232 |
| --- | --- | --- | --- | --- | --- |
| nerve | 64 | 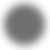 | 1 | / | 15526 |

Breakdown by Health State

| normal | 1 | 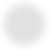 | 5 | / | 3328058 |
| --- | --- | --- | --- | --- | --- |

Breakdown by Developmental Stage

| fetus | 7 | 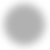 | 4 | / | 556801 |
| --- | --- | --- | --- | --- | --- |

**^2^:The EST profile was derived from UniGene EST profile and was filtered so that items with zero transcripts per million(TPM) and zero Gene EST were discarded. The following EST profiles are done in the same way.**

**BLAST result^3^：**

| Accession | Description | [Max score](http://blast.ncbi.nlm.nih.gov/Blast.cgi?CMD=Get&ALIGNMENTS=100&ALIGNMENT_VIEW=Pairwise&BLAST_SPEC=OGP__9606__9558&DATABASE_SORT=0&DESCRIPTIONS=100&FIRST_QUERY_NUM=0&FORMAT_OBJECT=Alignment&FORMAT_PAGE_TARGET=&FORMAT_TYPE=HTML&GET_SEQUENCE=yes&I_THRESH=&MASK_CHAR=2&MASK_COLOR=1&NEW_VIEW=yes&NUM_OVERVIEW=100&OLD_BLAST=false&PAGE=Nucleotides&QUERY_INDEX=0&QUERY_NUMBER=0&RESULTS_PAGE_TARGET=&RID=T5JCPGBY01S&SHOW_LINKOUT=yes&SHOW_OVERVIEW=yes&STEP_NUMBER=&WORD_SIZE=11&DISPLAY_SORT=1&HSP_SORT=1#sort_mark) | [Total score](http://blast.ncbi.nlm.nih.gov/Blast.cgi?CMD=Get&ALIGNMENTS=100&ALIGNMENT_VIEW=Pairwise&BLAST_SPEC=OGP__9606__9558&DATABASE_SORT=0&DESCRIPTIONS=100&FIRST_QUERY_NUM=0&FORMAT_OBJECT=Alignment&FORMAT_PAGE_TARGET=&FORMAT_TYPE=HTML&GET_SEQUENCE=yes&I_THRESH=&MASK_CHAR=2&MASK_COLOR=1&NEW_VIEW=yes&NUM_OVERVIEW=100&OLD_BLAST=false&PAGE=Nucleotides&QUERY_INDEX=0&QUERY_NUMBER=0&RESULTS_PAGE_TARGET=&RID=T5JCPGBY01S&SHOW_LINKOUT=yes&SHOW_OVERVIEW=yes&STEP_NUMBER=&WORD_SIZE=11&DISPLAY_SORT=2&HSP_SORT=1#sort_mark) | [Query coverage](http://blast.ncbi.nlm.nih.gov/Blast.cgi?CMD=Get&ALIGNMENTS=100&ALIGNMENT_VIEW=Pairwise&BLAST_SPEC=OGP__9606__9558&DATABASE_SORT=0&DESCRIPTIONS=100&FIRST_QUERY_NUM=0&FORMAT_OBJECT=Alignment&FORMAT_PAGE_TARGET=&FORMAT_TYPE=HTML&GET_SEQUENCE=yes&I_THRESH=&MASK_CHAR=2&MASK_COLOR=1&NEW_VIEW=yes&NUM_OVERVIEW=100&OLD_BLAST=false&PAGE=Nucleotides&QUERY_INDEX=0&QUERY_NUMBER=0&RESULTS_PAGE_TARGET=&RID=T5JCPGBY01S&SHOW_LINKOUT=yes&SHOW_OVERVIEW=yes&STEP_NUMBER=&WORD_SIZE=11&DISPLAY_SORT=4&HSP_SORT=0#sort_mark) | [E value](http://blast.ncbi.nlm.nih.gov/Blast.cgi?CMD=Get&ALIGNMENTS=100&ALIGNMENT_VIEW=Pairwise&BLAST_SPEC=OGP__9606__9558&DATABASE_SORT=0&DESCRIPTIONS=100&FIRST_QUERY_NUM=0&FORMAT_OBJECT=Alignment&FORMAT_PAGE_TARGET=&FORMAT_TYPE=HTML&GET_SEQUENCE=yes&I_THRESH=&MASK_CHAR=2&MASK_COLOR=1&NEW_VIEW=yes&NUM_OVERVIEW=100&OLD_BLAST=false&PAGE=Nucleotides&QUERY_INDEX=0&QUERY_NUMBER=0&RESULTS_PAGE_TARGET=&RID=T5JCPGBY01S&SHOW_LINKOUT=yes&SHOW_OVERVIEW=yes&STEP_NUMBER=&WORD_SIZE=11&DISPLAY_SORT=0&HSP_SORT=0#sort_mark) | [Max ident](http://blast.ncbi.nlm.nih.gov/Blast.cgi?CMD=Get&ALIGNMENTS=100&ALIGNMENT_VIEW=Pairwise&BLAST_SPEC=OGP__9606__9558&DATABASE_SORT=0&DESCRIPTIONS=100&FIRST_QUERY_NUM=0&FORMAT_OBJECT=Alignment&FORMAT_PAGE_TARGET=&FORMAT_TYPE=HTML&GET_SEQUENCE=yes&I_THRESH=&MASK_CHAR=2&MASK_COLOR=1&NEW_VIEW=yes&NUM_OVERVIEW=100&OLD_BLAST=false&PAGE=Nucleotides&QUERY_INDEX=0&QUERY_NUMBER=0&RESULTS_PAGE_TARGET=&RID=T5JCPGBY01S&SHOW_LINKOUT=yes&SHOW_OVERVIEW=yes&STEP_NUMBER=&WORD_SIZE=11&DISPLAY_SORT=3&HSP_SORT=3#sort_mark) |
| --- | --- | --- | --- | --- | --- | --- |
| [NG_011649.1](http://www.ncbi.nlm.nih.gov/nucleotide/225543301?report=genbank&log$=nucltop&blast_rank=15&RID=T5FXUVS201N) | Homo sapiens ficolin (collagen/fibrinogen domain containing lectin) 2 (hucolin) (FCN2), RefSeqGene on chromosome 9 | [41.0](http://blast.ncbi.nlm.nih.gov/Blast.cgi#225543301) | 41.0 | 4% | 3.2 | 93% |

**^3^：the blast was done in NCBI by blastn, and the species genome is Human. For ‘Search set’ we chose ‘RefSeq Genomic’ and for ‘Program Selection’ we chose ‘Somewhat similar sequences (blastn)’. Other parameters were set default. The following blast results are done in the same way.**

1. **BM832957:**

[Transcribed locus](http://www.ncbi.nlm.nih.gov/UniGene/clust.cgi?UGID=133751&TAXID=9606&SEARCH=BM832957)

Homo sapiens

Hs.17910: 18 sequences.

**Transcribed locus**

**GENE EXPRESSION**

Tissues and development stages from this gene's sequences survey gene expression. Links to other NCBI expression resources.

|  | Restricted Expression: | glioma [[show more like this](http://www.ncbi.nlm.nih.gov/sites/entrez?db=unigene&cmd=search&term=9606%5Btaxid%5D AND  glioma%5Brestricted%5D)] |
| --- | --- | --- |
|  | [EST Profile](http://www.ncbi.nlm.nih.gov/UniGene/ESTProfileViewer.cgi?uglist=Hs.17910): | Approximate expression patterns inferred from EST sources. [[Show more entries with profiles like this](http://www.ncbi.nlm.nih.gov/sites/entrez?DB=unigene&DbFrom=unigene&IdsFromResult=133751&cmd=Link&LinkName=unigene_unigene_expression&tool=UniGene.clust)] |
|  | [GEO Profiles](http://www.ncbi.nlm.nih.gov/sites/entrez?DB=geoprofiles&DbFrom=unigene&IdsFromResult=133751&cmd=Link&LinkName=unigene_geoprofiles&tool=UniGene.clust): | Experimental gene expression data (Gene Expression Omnibus). |
|  | cDNA Sources: | mixed; placenta; brain; skin; eye; uncharacterized tissue; stomach; ovary; prostate |

**MAPPING POSITION**

Genomic location specified by transcript mapping, radiation hybrid mapping, genetic mapping or cytogenetic mapping.

|  | Chromosome: | 2 |  | |
| --- | --- | --- | --- | --- |
|  | UniSTS entry: | Chr 2 | [SHGC-33819](http://www.ncbi.nlm.nih.gov/genome/sts/sts.cgi?uid=15979) | [[Map Viewer](http://www.ncbi.nlm.nih.gov/mapview/maps.cgi?taxid=9606&chr=2&MAPS=wirh-r,ncbirh-r,shgcg3-r,tng-r,g3-r,gb4-r&sts=15979)] |
|  | UniSTS entry: | Chr 2 | [RH70496](http://www.ncbi.nlm.nih.gov/genome/sts/sts.cgi?uid=72467) |  |

**SEQUENCES**

*Sequences representing this gene; mRNAs, ESTs, and gene predictions supported by transcribed sequences.*

**EST sequences (18)**

|  | [BX102746.1](http://www.ncbi.nlm.nih.gov/UniGene/seq.cgi?ORG=Hs&SID=11128660) | Clone IMAGp998E22113_;_IMAGE:121341 | mixed |  |  |
| --- | --- | --- | --- | --- | --- |
|  | [AI360054.1](http://www.ncbi.nlm.nih.gov/UniGene/seq.cgi?ORG=Hs&SID=1241499) | Clone IMAGE:2018565 | brain | 3' read |  |
|  | [AI936609.1](http://www.ncbi.nlm.nih.gov/UniGene/seq.cgi?ORG=Hs&SID=1536708) | Clone IMAGE:2329620 | mixed | 3' read |  |
|  | [AI648552.1](http://www.ncbi.nlm.nih.gov/UniGene/seq.cgi?ORG=Hs&SID=1544341) | Clone IMAGE:2292504 | ovary | 3' read |  |
|  | [AI871280.1](http://www.ncbi.nlm.nih.gov/UniGene/seq.cgi?ORG=Hs&SID=1553072) | Clone IMAGE:2431258 | brain | 3' read |  |
|  | [BX365990.2](http://www.ncbi.nlm.nih.gov/UniGene/seq.cgi?ORG=Hs&SID=15540006) | Clone CS0DI066YC14 | placenta | 5' read |  |
|  | [BX360143.2](http://www.ncbi.nlm.nih.gov/UniGene/seq.cgi?ORG=Hs&SID=15542681) | Clone CS0DI066YC14 | placenta | 3' read |  |
|  | [BX360144.2](http://www.ncbi.nlm.nih.gov/UniGene/seq.cgi?ORG=Hs&SID=15543628) | Clone CS0DI066YC14 | placenta | 5' read |  |
|  | [AW975290.1](http://www.ncbi.nlm.nih.gov/UniGene/seq.cgi?ORG=Hs&SID=2009985) |  | uncharacterized tissue |  |  |
|  | [H40146.1](http://www.ncbi.nlm.nih.gov/UniGene/seq.cgi?ORG=Hs&SID=236396) | Clone IMAGE:191742 | mixed | 3' read |  |
|  | [ES310521.1](http://www.ncbi.nlm.nih.gov/UniGene/seq.cgi?ORG=Hs&SID=37953149) |  | skin |  | **A** |
|  | [BM684333.1](http://www.ncbi.nlm.nih.gov/UniGene/seq.cgi?ORG=Hs&SID=4100030) | Clone UI-E-EJ1-aji-d-10-0-UI | eye | 3' read | **A** |
|  | [BM829694.1](http://www.ncbi.nlm.nih.gov/UniGene/seq.cgi?ORG=Hs&SID=4225046) | Clone S5SNU484s1-9-D12 | stomach | 5' read | **A** |
|  | [BM832957.1](http://www.ncbi.nlm.nih.gov/UniGene/seq.cgi?ORG=Hs&SID=4228309) | Clone S5SNU484s1-10-G04 | stomach | 5' read | **A** |
|  | [BM929902.1](http://www.ncbi.nlm.nih.gov/UniGene/seq.cgi?ORG=Hs&SID=4669714) | Clone UI-E-EJ1-aji-d-10-0-UI | eye | 5' read |  |
|  | [T96871.1](http://www.ncbi.nlm.nih.gov/UniGene/seq.cgi?ORG=Hs&SID=84923) | Clone IMAGE:121341 | mixed | 3' read |  |
|  | [T98675.1](http://www.ncbi.nlm.nih.gov/UniGene/seq.cgi?ORG=Hs&SID=86857) | Clone IMAGE:122261 | mixed | 3' read |  |
|  | [AA659718.1](http://www.ncbi.nlm.nih.gov/UniGene/seq.cgi?ORG=Hs&SID=870489) | Clone IMAGE:1218923 | prostate | 3' read |  |

# EST Profile：

[Hs.17910](http://www.ncbi.nlm.nih.gov/UniGene/clust.cgi?ORG=Hs&CID=17910) - Transcribed locus

Breakdown by Body Sites

| eye | 9 | 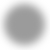 | 2 | / | 208810 |
| --- | --- | --- | --- | --- | --- |
| ovary | 9 | 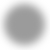 | 1 | / | 101482 |
| placenta | 10 | 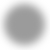 | 3 | / | 282968 |
| prostate | 5 | 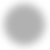 | 1 | / | 189585 |
| skin | 4 | 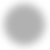 | 1 | / | 210718 |
| stomach | 20 | 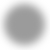 | 2 | / | 95775 |

Breakdown by Health State

| glioma | 18 | 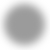 | 2 | / | 107167 |
| --- | --- | --- | --- | --- | --- |

| normal | 3 | 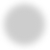 | 12 | / | 3328058 |
| --- | --- | --- | --- | --- | --- |
| fetus | 8 | 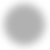 | 5 | / | 556801 |

**BLAST result：**

| Accession | Description | [Max score](http://blast.ncbi.nlm.nih.gov/Blast.cgi?CMD=Get&ALIGNMENTS=100&ALIGNMENT_VIEW=Pairwise&BLAST_SPEC=OGP__9606__9558&DATABASE_SORT=0&DESCRIPTIONS=100&FIRST_QUERY_NUM=0&FORMAT_OBJECT=Alignment&FORMAT_PAGE_TARGET=&FORMAT_TYPE=HTML&GET_SEQUENCE=yes&I_THRESH=&MASK_CHAR=2&MASK_COLOR=1&NEW_VIEW=yes&NUM_OVERVIEW=100&OLD_BLAST=false&PAGE=Nucleotides&QUERY_INDEX=0&QUERY_NUMBER=0&RESULTS_PAGE_TARGET=&RID=UKJX24N2012&SHOW_LINKOUT=yes&SHOW_OVERVIEW=yes&STEP_NUMBER=&WORD_SIZE=11&DISPLAY_SORT=1&HSP_SORT=1#sort_mark) | [Total score](http://blast.ncbi.nlm.nih.gov/Blast.cgi?CMD=Get&ALIGNMENTS=100&ALIGNMENT_VIEW=Pairwise&BLAST_SPEC=OGP__9606__9558&DATABASE_SORT=0&DESCRIPTIONS=100&FIRST_QUERY_NUM=0&FORMAT_OBJECT=Alignment&FORMAT_PAGE_TARGET=&FORMAT_TYPE=HTML&GET_SEQUENCE=yes&I_THRESH=&MASK_CHAR=2&MASK_COLOR=1&NEW_VIEW=yes&NUM_OVERVIEW=100&OLD_BLAST=false&PAGE=Nucleotides&QUERY_INDEX=0&QUERY_NUMBER=0&RESULTS_PAGE_TARGET=&RID=UKJX24N2012&SHOW_LINKOUT=yes&SHOW_OVERVIEW=yes&STEP_NUMBER=&WORD_SIZE=11&DISPLAY_SORT=2&HSP_SORT=1#sort_mark) | [Query coverage](http://blast.ncbi.nlm.nih.gov/Blast.cgi?CMD=Get&ALIGNMENTS=100&ALIGNMENT_VIEW=Pairwise&BLAST_SPEC=OGP__9606__9558&DATABASE_SORT=0&DESCRIPTIONS=100&FIRST_QUERY_NUM=0&FORMAT_OBJECT=Alignment&FORMAT_PAGE_TARGET=&FORMAT_TYPE=HTML&GET_SEQUENCE=yes&I_THRESH=&MASK_CHAR=2&MASK_COLOR=1&NEW_VIEW=yes&NUM_OVERVIEW=100&OLD_BLAST=false&PAGE=Nucleotides&QUERY_INDEX=0&QUERY_NUMBER=0&RESULTS_PAGE_TARGET=&RID=UKJX24N2012&SHOW_LINKOUT=yes&SHOW_OVERVIEW=yes&STEP_NUMBER=&WORD_SIZE=11&DISPLAY_SORT=4&HSP_SORT=0#sort_mark) | [E value](http://blast.ncbi.nlm.nih.gov/Blast.cgi?CMD=Get&ALIGNMENTS=100&ALIGNMENT_VIEW=Pairwise&BLAST_SPEC=OGP__9606__9558&DATABASE_SORT=0&DESCRIPTIONS=100&FIRST_QUERY_NUM=0&FORMAT_OBJECT=Alignment&FORMAT_PAGE_TARGET=&FORMAT_TYPE=HTML&GET_SEQUENCE=yes&I_THRESH=&MASK_CHAR=2&MASK_COLOR=1&NEW_VIEW=yes&NUM_OVERVIEW=100&OLD_BLAST=false&PAGE=Nucleotides&QUERY_INDEX=0&QUERY_NUMBER=0&RESULTS_PAGE_TARGET=&RID=UKJX24N2012&SHOW_LINKOUT=yes&SHOW_OVERVIEW=yes&STEP_NUMBER=&WORD_SIZE=11&DISPLAY_SORT=0&HSP_SORT=0#sort_mark) | [Max ident](http://blast.ncbi.nlm.nih.gov/Blast.cgi?CMD=Get&ALIGNMENTS=100&ALIGNMENT_VIEW=Pairwise&BLAST_SPEC=OGP__9606__9558&DATABASE_SORT=0&DESCRIPTIONS=100&FIRST_QUERY_NUM=0&FORMAT_OBJECT=Alignment&FORMAT_PAGE_TARGET=&FORMAT_TYPE=HTML&GET_SEQUENCE=yes&I_THRESH=&MASK_CHAR=2&MASK_COLOR=1&NEW_VIEW=yes&NUM_OVERVIEW=100&OLD_BLAST=false&PAGE=Nucleotides&QUERY_INDEX=0&QUERY_NUMBER=0&RESULTS_PAGE_TARGET=&RID=UKJX24N2012&SHOW_LINKOUT=yes&SHOW_OVERVIEW=yes&STEP_NUMBER=&WORD_SIZE=11&DISPLAY_SORT=3&HSP_SORT=3#sort_mark) |  |
| --- | --- | --- | --- | --- | --- | --- | --- |
| [NG_032003.1](http://www.ncbi.nlm.nih.gov/nucleotide/372266088?report=genbank&log$=nucltop&blast_rank=26&RID=UKJX24N2012) | Homo sapiens ankyrin repeat domain 11 (ANKRD11), RefSeqGene on chromosome 16 | [39.2](http://blast.ncbi.nlm.nih.gov/Blast.cgi#372266088) | 39.2 | 11% | 5.3 | 86% |  |
| [NG_030466.1](http://www.ncbi.nlm.nih.gov/nucleotide/355390230?report=genbank&log$=nucltop&blast_rank=27&RID=UKJX24N2012) | Homo sapiens cadherin, EGF LAG seven-pass G-type receptor 1 (flamingo homolog, Drosophila) (CELSR1), RefSeqGene on chromosome 22 | [39.2](http://blast.ncbi.nlm.nih.gov/Blast.cgi#355390230) | 39.2 | 14% | 5.3 | 80% |  |
| [NG_029856.1](http://www.ncbi.nlm.nih.gov/nucleotide/345197223?report=genbank&log$=nucltop&blast_rank=28&RID=UKJX24N2012) | Homo sapiens carbohydrate (N-acetylgalactosamine 4-0) sulfotransferase 9 (CHST9), RefSeqGene on chromosome 18 | [39.2](http://blast.ncbi.nlm.nih.gov/Blast.cgi#345197223) | 39.2 | 14% | 5.3 | 80% |  |
| [NG_029184.1](http://www.ncbi.nlm.nih.gov/nucleotide/338221685?report=genbank&log$=nucltop&blast_rank=29&RID=UKJX24N2012) | Homo sapiens mitochondrial methionyl-tRNA formyltransferase (MTFMT), RefSeqGene on chromosome 15 | [39.2](http://blast.ncbi.nlm.nih.gov/Blast.cgi#338221685) | 39.2 | 8% | 5.3 | 96% |  |
| [NG_017093.1](http://www.ncbi.nlm.nih.gov/nucleotide/294610648?report=genbank&log$=nucltop&blast_rank=30&RID=UKJX24N2012) | Homo sapiens mastermind-like domain containing 1 (MAMLD1), RefSeqGene on chromosome X | [39.2](http://blast.ncbi.nlm.nih.gov/Blast.cgi#294610648) | 39.2 | 9% | 5.3 | 90% |  |
| [NG_016243.1](http://www.ncbi.nlm.nih.gov/nucleotide/283837925?report=genbank&log$=nucltop&blast_rank=31&RID=UKJX24N2012) | Homo sapiens gap junction protein, beta 4, 30.3kDa (GJB4), RefSeqGene on chromosome 1 | [39.2](http://blast.ncbi.nlm.nih.gov/Blast.cgi#283837925) | 39.2 | 9% | 5.3 | 90% |  |

1. **AL117453:**

[MRNA; cDNA DKFZp586G1917 (from clone DKFZp586G1917)](http://www.ncbi.nlm.nih.gov/UniGene/clust.cgi?UGID=2742104&TAXID=9606&SEARCH=AL117453)

Homo sapiens

Hs.672786: 1 sequences

**MRNA; cDNA DKFZp586G1917 (from clone DKFZp586G1917)**

**GENE EXPRESSION**

Tissues and development stages from this gene's sequences survey gene expression. Links to other NCBI expression resources.

|  | [GEO Profiles](http://www.ncbi.nlm.nih.gov/sites/entrez?DB=geoprofiles&DbFrom=unigene&IdsFromResult=2742104&cmd=Link&LinkName=unigene_geoprofiles&tool=UniGene.clust): | Experimental gene expression data (Gene Expression Omnibus). |
| --- | --- | --- |

**SEQUENCES**

*Sequences representing this gene; mRNAs, ESTs, and gene predictions supported by transcribed sequences.*

**mRNA sequences (1)**

|  | [AL117453.1](http://www.ncbi.nlm.nih.gov/UniGene/seq.cgi?ORG=Hs&SID=1637899) | Homo sapiens mRNA; cDNA DKFZp586G1917 (from clone DKFZp586G1917) | **A** |
| --- | --- | --- | --- |

**BLAST result：**

| Accession | Description | [Max score](http://blast.ncbi.nlm.nih.gov/Blast.cgi?CMD=Get&ALIGNMENTS=100&ALIGNMENT_VIEW=Pairwise&BLAST_SPEC=OGP__9606__9558&DATABASE_SORT=0&DESCRIPTIONS=100&FIRST_QUERY_NUM=0&FORMAT_OBJECT=Alignment&FORMAT_PAGE_TARGET=&FORMAT_TYPE=HTML&GET_SEQUENCE=yes&I_THRESH=&MASK_CHAR=2&MASK_COLOR=1&NEW_VIEW=yes&NUM_OVERVIEW=100&OLD_BLAST=false&PAGE=Nucleotides&QUERY_INDEX=0&QUERY_NUMBER=0&RESULTS_PAGE_TARGET=&RID=T5JCPGBY01S&SHOW_LINKOUT=yes&SHOW_OVERVIEW=yes&STEP_NUMBER=&WORD_SIZE=11&DISPLAY_SORT=1&HSP_SORT=1#sort_mark) | | | [Total score](http://blast.ncbi.nlm.nih.gov/Blast.cgi?CMD=Get&ALIGNMENTS=100&ALIGNMENT_VIEW=Pairwise&BLAST_SPEC=OGP__9606__9558&DATABASE_SORT=0&DESCRIPTIONS=100&FIRST_QUERY_NUM=0&FORMAT_OBJECT=Alignment&FORMAT_PAGE_TARGET=&FORMAT_TYPE=HTML&GET_SEQUENCE=yes&I_THRESH=&MASK_CHAR=2&MASK_COLOR=1&NEW_VIEW=yes&NUM_OVERVIEW=100&OLD_BLAST=false&PAGE=Nucleotides&QUERY_INDEX=0&QUERY_NUMBER=0&RESULTS_PAGE_TARGET=&RID=T5JCPGBY01S&SHOW_LINKOUT=yes&SHOW_OVERVIEW=yes&STEP_NUMBER=&WORD_SIZE=11&DISPLAY_SORT=2&HSP_SORT=1#sort_mark) | [Query coverage](http://blast.ncbi.nlm.nih.gov/Blast.cgi?CMD=Get&ALIGNMENTS=100&ALIGNMENT_VIEW=Pairwise&BLAST_SPEC=OGP__9606__9558&DATABASE_SORT=0&DESCRIPTIONS=100&FIRST_QUERY_NUM=0&FORMAT_OBJECT=Alignment&FORMAT_PAGE_TARGET=&FORMAT_TYPE=HTML&GET_SEQUENCE=yes&I_THRESH=&MASK_CHAR=2&MASK_COLOR=1&NEW_VIEW=yes&NUM_OVERVIEW=100&OLD_BLAST=false&PAGE=Nucleotides&QUERY_INDEX=0&QUERY_NUMBER=0&RESULTS_PAGE_TARGET=&RID=T5JCPGBY01S&SHOW_LINKOUT=yes&SHOW_OVERVIEW=yes&STEP_NUMBER=&WORD_SIZE=11&DISPLAY_SORT=4&HSP_SORT=0#sort_mark) | | [E value](http://blast.ncbi.nlm.nih.gov/Blast.cgi?CMD=Get&ALIGNMENTS=100&ALIGNMENT_VIEW=Pairwise&BLAST_SPEC=OGP__9606__9558&DATABASE_SORT=0&DESCRIPTIONS=100&FIRST_QUERY_NUM=0&FORMAT_OBJECT=Alignment&FORMAT_PAGE_TARGET=&FORMAT_TYPE=HTML&GET_SEQUENCE=yes&I_THRESH=&MASK_CHAR=2&MASK_COLOR=1&NEW_VIEW=yes&NUM_OVERVIEW=100&OLD_BLAST=false&PAGE=Nucleotides&QUERY_INDEX=0&QUERY_NUMBER=0&RESULTS_PAGE_TARGET=&RID=T5JCPGBY01S&SHOW_LINKOUT=yes&SHOW_OVERVIEW=yes&STEP_NUMBER=&WORD_SIZE=11&DISPLAY_SORT=0&HSP_SORT=0#sort_mark) | | [Max ident](http://blast.ncbi.nlm.nih.gov/Blast.cgi?CMD=Get&ALIGNMENTS=100&ALIGNMENT_VIEW=Pairwise&BLAST_SPEC=OGP__9606__9558&DATABASE_SORT=0&DESCRIPTIONS=100&FIRST_QUERY_NUM=0&FORMAT_OBJECT=Alignment&FORMAT_PAGE_TARGET=&FORMAT_TYPE=HTML&GET_SEQUENCE=yes&I_THRESH=&MASK_CHAR=2&MASK_COLOR=1&NEW_VIEW=yes&NUM_OVERVIEW=100&OLD_BLAST=false&PAGE=Nucleotides&QUERY_INDEX=0&QUERY_NUMBER=0&RESULTS_PAGE_TARGET=&RID=T5JCPGBY01S&SHOW_LINKOUT=yes&SHOW_OVERVIEW=yes&STEP_NUMBER=&WORD_SIZE=11&DISPLAY_SORT=3&HSP_SORT=3#sort_mark) |  |
| --- | --- | --- | --- | --- | --- | --- | --- | --- | --- | --- | --- |
| [NG_021287.1](http://www.ncbi.nlm.nih.gov/nucleotide/296531468?report=genbank&log$=nucltop&blast_rank=34&RID=T5GY3XK601S) | Homo sapiens component of oligomeric golgi complex 7 (COG7), RefSeqGene on chromosome 16 | [59.0](http://blast.ncbi.nlm.nih.gov/Blast.cgi#296531468) | | | 59.0 | 1% | | 8e-05 | | 82% |  |
| [NG_032166.1](http://www.ncbi.nlm.nih.gov/nucleotide/381140021?report=genbank&log$=nucltop&blast_rank=57&RID=T5GY3XK601S) | Homo sapiens SEC24 family, member B (S. cerevisiae) (SEC24B), RefSeqGene on chromosome 4 | [50.0](http://blast.ncbi.nlm.nih.gov/Blast.cgi#381140021) | | | 50.0 | 2% | | 0.039 | | 72% |  |
| [NG_029450.1](http://www.ncbi.nlm.nih.gov/nucleotide/340139193?report=genbank&log$=nucltop&blast_rank=78&RID=T5GY3XK601S) | Homo sapiens suppression of tumorigenicity 5 (ST5), RefSeqGene on chromosome 11 | [48.2](http://blast.ncbi.nlm.nih.gov/Blast.cgi#340139193) | | | 48.2 | 1% | | 0.14 | | 85% |  |
| [NG_012222.1](http://www.ncbi.nlm.nih.gov/nucleotide/238018129?report=genbank&log$=nucltop&blast_rank=79&RID=T5GY3XK601S) | Homo sapiens interleukin 21 receptor (IL21R), RefSeqGene on chromosome 16 | [48.2](http://blast.ncbi.nlm.nih.gov/Blast.cgi#238018129) | | | 48.2 | 0% | | 0.14 | | 97% |  |
| [NG_029742.1](http://www.ncbi.nlm.nih.gov/nucleotide/343488439?report=genbank&log$=nucltop&blast_rank=93&RID=T5GY3XK601S) | Homo sapiens endoplasmic reticulum lectin 1 (ERLEC1), RefSeqGene on chromosome 2 | | [46.4](http://blast.ncbi.nlm.nih.gov/Blast.cgi#343488439) | 46.4 | | | 1% | | 0.48 | 85% | |
| [NG_029635.1](http://www.ncbi.nlm.nih.gov/nucleotide/343098473?report=genbank&log$=nucltop&blast_rank=94&RID=T5GY3XK601S) | Homo sapiens protease, serine, 3 (PRSS3), RefSeqGene on chromosome 9 | | [46.4](http://blast.ncbi.nlm.nih.gov/Blast.cgi#343098473) | 46.4 | | | 0% | | 0.48 | 91% | |

1. **BM983734:**

[Transcribed locus](http://www.ncbi.nlm.nih.gov/UniGene/clust.cgi?UGID=2751402&TAXID=9606&SEARCH=BM983734)

Homo sapiens

Hs.682084: 1 sequences.

**Transcribed locus**

**GENE EXPRESSION**

Tissues and development stages from this gene's sequences survey gene expression. Links to other NCBI expression resources.

|  | [EST Profile](http://www.ncbi.nlm.nih.gov/UniGene/ESTProfileViewer.cgi?uglist=Hs.682084): | Approximate expression patterns inferred from EST sources. |
| --- | --- | --- |
|  | [GEO Profiles](http://www.ncbi.nlm.nih.gov/sites/entrez?DB=geoprofiles&DbFrom=unigene&IdsFromResult=2751402&cmd=Link&LinkName=unigene_geoprofiles&tool=UniGene.clust): | Experimental gene expression data (Gene Expression Omnibus). |
|  | cDNA Sources: | lung |

**MAPPING POSITION**

Genomic location specified by transcript mapping, radiation hybrid mapping, genetic mapping or cytogenetic mapping.

|  | UniSTS entry: | Chr 8 | [REN13617](http://www.ncbi.nlm.nih.gov/genome/sts/sts.cgi?uid=338417) |
| --- | --- | --- | --- |

**SEQUENCES**

*Sequences representing this gene; mRNAs, ESTs, and gene predictions supported by transcribed sequences.*

**EST sequences (1)**

|  | [BM983734.1](http://www.ncbi.nlm.nih.gov/UniGene/seq.cgi?ORG=Hs&SID=4301694) | Clone UI-CF-DU1-aay-c-11-0-UI | lung | 3' read | **A** |
| --- | --- | --- | --- | --- | --- |

# EST Profile：

[Hs.682084](http://www.ncbi.nlm.nih.gov/UniGene/clust.cgi?ORG=Hs&CID=682084) - Transcribed locus

Breakdown by Body Sites

| lung | 2 | 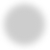 | 1 | / | 334751 |
| --- | --- | --- | --- | --- | --- |

**BLAST result：**

| Accession | Description | [Max score](http://blast.ncbi.nlm.nih.gov/Blast.cgi?CMD=Get&ALIGNMENTS=100&ALIGNMENT_VIEW=Pairwise&BLAST_SPEC=OGP__9606__9558&DATABASE_SORT=0&DESCRIPTIONS=100&FIRST_QUERY_NUM=0&FORMAT_OBJECT=Alignment&FORMAT_PAGE_TARGET=&FORMAT_TYPE=HTML&GET_SEQUENCE=yes&I_THRESH=&MASK_CHAR=2&MASK_COLOR=1&NEW_VIEW=yes&NUM_OVERVIEW=100&OLD_BLAST=false&PAGE=Nucleotides&QUERY_INDEX=0&QUERY_NUMBER=0&RESULTS_PAGE_TARGET=&RID=T5JCPGBY01S&SHOW_LINKOUT=yes&SHOW_OVERVIEW=yes&STEP_NUMBER=&WORD_SIZE=11&DISPLAY_SORT=1&HSP_SORT=1#sort_mark) | [Total score](http://blast.ncbi.nlm.nih.gov/Blast.cgi?CMD=Get&ALIGNMENTS=100&ALIGNMENT_VIEW=Pairwise&BLAST_SPEC=OGP__9606__9558&DATABASE_SORT=0&DESCRIPTIONS=100&FIRST_QUERY_NUM=0&FORMAT_OBJECT=Alignment&FORMAT_PAGE_TARGET=&FORMAT_TYPE=HTML&GET_SEQUENCE=yes&I_THRESH=&MASK_CHAR=2&MASK_COLOR=1&NEW_VIEW=yes&NUM_OVERVIEW=100&OLD_BLAST=false&PAGE=Nucleotides&QUERY_INDEX=0&QUERY_NUMBER=0&RESULTS_PAGE_TARGET=&RID=T5JCPGBY01S&SHOW_LINKOUT=yes&SHOW_OVERVIEW=yes&STEP_NUMBER=&WORD_SIZE=11&DISPLAY_SORT=2&HSP_SORT=1#sort_mark) | [Query coverage](http://blast.ncbi.nlm.nih.gov/Blast.cgi?CMD=Get&ALIGNMENTS=100&ALIGNMENT_VIEW=Pairwise&BLAST_SPEC=OGP__9606__9558&DATABASE_SORT=0&DESCRIPTIONS=100&FIRST_QUERY_NUM=0&FORMAT_OBJECT=Alignment&FORMAT_PAGE_TARGET=&FORMAT_TYPE=HTML&GET_SEQUENCE=yes&I_THRESH=&MASK_CHAR=2&MASK_COLOR=1&NEW_VIEW=yes&NUM_OVERVIEW=100&OLD_BLAST=false&PAGE=Nucleotides&QUERY_INDEX=0&QUERY_NUMBER=0&RESULTS_PAGE_TARGET=&RID=T5JCPGBY01S&SHOW_LINKOUT=yes&SHOW_OVERVIEW=yes&STEP_NUMBER=&WORD_SIZE=11&DISPLAY_SORT=4&HSP_SORT=0#sort_mark) | [E value](http://blast.ncbi.nlm.nih.gov/Blast.cgi?CMD=Get&ALIGNMENTS=100&ALIGNMENT_VIEW=Pairwise&BLAST_SPEC=OGP__9606__9558&DATABASE_SORT=0&DESCRIPTIONS=100&FIRST_QUERY_NUM=0&FORMAT_OBJECT=Alignment&FORMAT_PAGE_TARGET=&FORMAT_TYPE=HTML&GET_SEQUENCE=yes&I_THRESH=&MASK_CHAR=2&MASK_COLOR=1&NEW_VIEW=yes&NUM_OVERVIEW=100&OLD_BLAST=false&PAGE=Nucleotides&QUERY_INDEX=0&QUERY_NUMBER=0&RESULTS_PAGE_TARGET=&RID=T5JCPGBY01S&SHOW_LINKOUT=yes&SHOW_OVERVIEW=yes&STEP_NUMBER=&WORD_SIZE=11&DISPLAY_SORT=0&HSP_SORT=0#sort_mark) | [Max ident](http://blast.ncbi.nlm.nih.gov/Blast.cgi?CMD=Get&ALIGNMENTS=100&ALIGNMENT_VIEW=Pairwise&BLAST_SPEC=OGP__9606__9558&DATABASE_SORT=0&DESCRIPTIONS=100&FIRST_QUERY_NUM=0&FORMAT_OBJECT=Alignment&FORMAT_PAGE_TARGET=&FORMAT_TYPE=HTML&GET_SEQUENCE=yes&I_THRESH=&MASK_CHAR=2&MASK_COLOR=1&NEW_VIEW=yes&NUM_OVERVIEW=100&OLD_BLAST=false&PAGE=Nucleotides&QUERY_INDEX=0&QUERY_NUMBER=0&RESULTS_PAGE_TARGET=&RID=T5JCPGBY01S&SHOW_LINKOUT=yes&SHOW_OVERVIEW=yes&STEP_NUMBER=&WORD_SIZE=11&DISPLAY_SORT=3&HSP_SORT=3#sort_mark) |
| --- | --- | --- | --- | --- | --- | --- |
| [NG_007455.1](http://www.ncbi.nlm.nih.gov/nucleotide/170014701?report=genbank&log$=nucltop&blast_rank=2&RID=T5HSXCTW01S) | Homo sapiens exostosin 1 (EXT1), RefSeqGene on chromosome 8 | [948](http://blast.ncbi.nlm.nih.gov/Blast.cgi#170014701) | 948 | 90% | 0.0 | 99% |
| [NG_013069.1](http://www.ncbi.nlm.nih.gov/nucleotide/261245120?report=genbank&log$=nucltop&blast_rank=17&RID=T5HSXCTW01S) | Homo sapiens integral membrane protein 2B (ITM2B), RefSeqGene on chromosome 13 | [50.0](http://blast.ncbi.nlm.nih.gov/Blast.cgi#261245120) | 50.0 | 7% | 0.006 | 86% |
| [NG_011729.1](http://www.ncbi.nlm.nih.gov/nucleotide/225903388?report=genbank&log$=nucltop&blast_rank=20&RID=T5HSXCTW01S) | Homo sapiens roundabout, axon guidance receptor, homolog 1 (Drosophila) (ROBO1), RefSeqGene on chromosome 3 | [48.2](http://blast.ncbi.nlm.nih.gov/Blast.cgi#225903388) | 48.2 | 9% | 0.022 | 79% |
| [NG_011688.1](http://www.ncbi.nlm.nih.gov/nucleotide/225637493?report=genbank&log$=nucltop&blast_rank=61&RID=T5HSXCTW01S) | Homo sapiens growth hormone receptor (GHR), RefSeqGene on chromosome 5 | [44.6](http://blast.ncbi.nlm.nih.gov/Blast.cgi#225637493) | 44.6 | 7% | 0.26 | 83% |
| [NG_012092.1](http://www.ncbi.nlm.nih.gov/nucleotide/237858691?report=genbank&log$=nucltop&blast_rank=82&RID=T5HSXCTW01S) | Homo sapiens insulin-like 3 (Leydig cell) (INSL3), RefSeqGene on chromosome 19 | [42.8](http://blast.ncbi.nlm.nih.gov/Blast.cgi#237858691) | 42.8 | 6% | 0.92 | 86% |

1. **BF507979:**

[Ca++-dependent secretion activator 2](http://www.ncbi.nlm.nih.gov/UniGene/clust.cgi?UGID=5867034&TAXID=9606&SEARCH=BF507979)

CADPS2, Homo sapiens

Hs.740589: 155 sequences.

[Order cDNA clone](http://www.ncbi.nlm.nih.gov/genome/clone/orderclone.cgi?db=unigene&uid=5867034)

**Ca++-dependent secretion activator 2 (CADPS2)**

**SELECTED PROTEIN SIMILARITIES**

Comparison of cluster transcripts with RefSeq proteins. The alignments can suggest function of the cluster.

| **Best Hits and Hits from model organisms** | | **Species** | **Id(%)** | **Len(aa)** |
| --- | --- | --- | --- | --- |
| [NP_001161412.1](javascript:PopUpMenu2_Set(Menu_prot269308219);) | calcium-dependent secretion activator 2 isoform c | *H. sapiens* | 100.0 | 1299 |
| [XP_527869.3](javascript:PopUpMenu2_Set(Menu_prot332868393);) | PREDICTED: calcium-dependent secretion activator 2 isoform 12 | *P. troglodytes* | 99.9 | 1299 |
| [XP_002803487.1](javascript:PopUpMenu2_Set(Menu_prot297289197);) | PREDICTED: calcium-dependent secretion activator 2-like | *M. mulatta* | 99.6 | 1300 |
| [NP_694803.3](javascript:PopUpMenu2_Set(Menu_prot118200358);) | calcium-dependent secretion activator 2 isoform 1 | *M. musculus* | 97.8 | 1303 |
| [NP_001086256.1](javascript:PopUpMenu2_Set(Menu_prot148231001);) | calcium-dependent secretion activator 1 | *X. laevis* | 83.3 | 1296 |
| [XP_002663813.2](javascript:PopUpMenu2_Set(Menu_prot326671787);) | PREDICTED: calcium-dependent secretion activator 1 | *D. rerio* | 81.1 | 1304 |
| [NP_001027030.1](javascript:PopUpMenu2_Set(Menu_prot78706458);) | calcium activated protein for secretion, isoform B | *D. melanogaster* | 67.2 | 1270 |
| [NP_502437.3](javascript:PopUpMenu2_Set(Menu_prot133931077);) | UNCoordinated family member (unc-31) | *C. elegans* | 58.7 | 1322 |
| **Other hits (26) [**[**Show subset**](http://www.ncbi.nlm.nih.gov/UniGene/clust.cgi?ORG=Hs&CID=740589)**]** | | **Species** | **Id(%)** | **Len(aa)** |
| [XP_003134777.1](javascript:PopUpMenu2_Set(Menu_prot311275524);) | PREDICTED: calcium-dependent secretion activator 2 isoform 2 | *S. scrofa* | 98.3 | 1300 |
| [XP_003364894.1](javascript:PopUpMenu2_Set(Menu_prot338724210);) | PREDICTED: calcium-dependent secretion activator 2 | *E. caballus* | 98.3 | 1299 |
| [XP_002712123.1](javascript:PopUpMenu2_Set(Menu_prot291391201);) | PREDICTED: Ca++-dependent secretion activator-like | *O. cuniculus* | 98.2 | 1301 |
| [NP_001095525.1](javascript:PopUpMenu2_Set(Menu_prot156120757);) | calcium-dependent secretion activator 2 | *B. taurus* | 98.0 | 1256 |
| [XP_532534.4](javascript:PopUpMenu2_Set(Menu_prot359321172);) | PREDICTED: calcium-dependent secretion activator 2 | *C. lupus familiaris* | 97.8 | 1201 |
| [XP_002726374.1](javascript:PopUpMenu2_Set(Menu_prot293346675);) | PREDICTED: calcium-dependent secretion activator 1-like isoform 2 | *R. norvegicus* | 97.6 | 1303 |
| [XP_001370502.1](javascript:PopUpMenu2_Set(Menu_prot126340727);) | PREDICTED: calcium-dependent secretion activator 2-like | *M. domestica* | 95.8 | 1302 |
| [XP_001507532.1](javascript:PopUpMenu2_Set(Menu_prot149411667);) | PREDICTED: calcium-dependent secretion activator 2-like | *O. anatinus* | 94.6 | 1246 |
| [XP_003202017.1](javascript:PopUpMenu2_Set(Menu_prot326911338);) | PREDICTED: calcium-dependent secretion activator 2-like | *M. gallopavo* | 93.4 | 1205 |
| [XP_003221376.1](javascript:PopUpMenu2_Set(Menu_prot327273215);) | PREDICTED: calcium-dependent secretion activator 2-like | *A. carolinensis* | 92.5 | 1300 |
| [XP_002935009.1](javascript:PopUpMenu2_Set(Menu_prot301610961);) | PREDICTED: calcium-dependent secretion activator 2-like isoform 1 | *X. tropicalis* | 88.9 | 1246 |
| [XP_001233869.1](javascript:PopUpMenu2_Set(Menu_prot118096982);) | PREDICTED: calcium-dependent secretion activator 1 isoform 3 | *G. gallus* | 83.8 | 1312 |
| [XP_002190062.1](javascript:PopUpMenu2_Set(Menu_prot224066421);) | PREDICTED: Ca2+-dependent secretion activator | *T. guttata* | 83.2 | 1331 |
| [XP_003448391.1](javascript:PopUpMenu2_Set(Menu_prot348521754);) | PREDICTED: calcium-dependent secretion activator 1 isoform 3 | *O. niloticus* | 79.0 | 1308 |
| [XP_796451.2](javascript:PopUpMenu2_Set(Menu_prot115733004);) | PREDICTED: similar to Ca2+-dependent activator protein; calcium-dependent actin-binding protein, partial | *S. purpuratus* | 74.7 | 577 |
| [XP_002736752.1](javascript:PopUpMenu2_Set(Menu_prot291233623);) | PREDICTED: Ca++-dependent secretion activator-like | *S. kowalevskii* | 74.0 | 1241 |
| [XP_002124364.1](javascript:PopUpMenu2_Set(Menu_prot198436344);) | PREDICTED: similar to Ca2+-dependent secretion activator | *C. intestinalis* | 72.5 | 1238 |
| [XP_972169.2](javascript:PopUpMenu2_Set(Menu_prot189233752);) | PREDICTED: similar to Calcium activated protein for secretion CG33653-PB | *T. castaneum* | 70.5 | 1246 |
| [XP_002606817.1](javascript:PopUpMenu2_Set(Menu_prot260823721);) | hypothetical protein BRAFLDRAFT_82458 | *B. floridae* | 69.5 | 1090 |
| [XP_002410287.1](javascript:PopUpMenu2_Set(Menu_prot241631822);) | Ca2+-dependent activator protein for secretion 2 isoform A, putative | *I. scapularis* | 68.5 | 1291 |
| [XP_002105793.1](javascript:PopUpMenu2_Set(Menu_prot195564370);) | GD24425 | *D. simulans* | 66.9 | 1146 |
| [XP_001637484.1](javascript:PopUpMenu2_Set(Menu_prot156396606);) | predicted protein | *N. vectensis* | 66.6 | 1146 |
| [XP_001604604.2](javascript:PopUpMenu2_Set(Menu_prot345486342);) | PREDICTED: calcium-dependent secretion activator-like | *N. vitripennis* | 64.5 | 1294 |
| [XP_392192.2](javascript:PopUpMenu2_Set(Menu_prot66503818);) | PREDICTED: calcium-dependent secretion activator | *A. mellifera* | 64.2 | 1293 |
| [XP_002155611.1](javascript:PopUpMenu2_Set(Menu_prot221130439);) | PREDICTED: similar to Ca2+-dependent secretion activator | *H. magnipapillata* | 63.0 | 1177 |
| [XP_002580285.1](javascript:PopUpMenu2_Set(Menu_prot256088309);) | calcium-dependent activator protein for secretion | *S. mansoni* | 53.2 | 663 |

**GENE EXPRESSION**

Tissues and development stages from this gene's sequences survey gene expression. Links to other NCBI expression resources.

|  | [EST Profile](http://www.ncbi.nlm.nih.gov/UniGene/ESTProfileViewer.cgi?uglist=Hs.740589): | Approximate expression patterns inferred from EST sources. |
| --- | --- | --- |
|  | cDNA Sources: | brain; uterus; adipose tissue; kidney; pancreas; liver; lung; intestine; embryonic tissue; mixed; stomach; prostate; uncharacterized tissue; connective tissue; bladder; spleen; testis; muscle; trachea; vascular; skin; mammary gland; salivary gland; thymus; thyroid; nerve; bone |

**MAPPING POSITION**

Genomic location specified by transcript mapping, radiation hybrid mapping, genetic mapping or cytogenetic mapping.

|  | Map position: | 7q31.3 |  | |
| --- | --- | --- | --- | --- |
|  | UniSTS entry: | Chr 7 | [RH46936](http://www.ncbi.nlm.nih.gov/genome/sts/sts.cgi?uid=68968) | [[Map Viewer](http://www.ncbi.nlm.nih.gov/mapview/maps.cgi?taxid=9606&chr=7&MAPS=ncbirh-r,gb4-r&sts=68968)] |
|  | UniSTS entry: | Chr 7 | [SHGC-32546](http://www.ncbi.nlm.nih.gov/genome/sts/sts.cgi?uid=19257) | [[Map Viewer](http://www.ncbi.nlm.nih.gov/mapview/maps.cgi?taxid=9606&chr=7&MAPS=wirh-r,ncbirh-r,shgcg3-r,tng-r,g3-r&sts=19257)] |
|  | UniSTS entry: | Chr 7 | [RH137681](http://www.ncbi.nlm.nih.gov/genome/sts/sts.cgi?uid=219768) |  |
|  | UniSTS entry: | Chr 7 | [D7S3237](http://www.ncbi.nlm.nih.gov/genome/sts/sts.cgi?uid=229491) |  |
|  | UniSTS entry: | Chr 7 | [D7S3235](http://www.ncbi.nlm.nih.gov/genome/sts/sts.cgi?uid=230826) |  |

**SEQUENCES**

*Sequences representing this gene; mRNAs, ESTs, and gene predictions supported by transcribed sequences.*

**mRNA sequences (15)**

|  | [AY264289.1](http://www.ncbi.nlm.nih.gov/UniGene/seq.cgi?ORG=Hs&SID=15632004) | Homo sapiens Ca2+-dependent activator protein for secretion 2 mRNA, complete cds | **P** |
| --- | --- | --- | --- |
|  | [BC054339.1](http://www.ncbi.nlm.nih.gov/UniGene/seq.cgi?ORG=Hs&SID=16176138) | Homo sapiens Ca++-dependent secretion activator 2, mRNA (cDNA clone IMAGE:6150934), partial cds | **PA** |
|  | [AK126518.1](http://www.ncbi.nlm.nih.gov/UniGene/seq.cgi?ORG=Hs&SID=16886186) | Homo sapiens cDNA FLJ44554 fis, clone UTERU3007419, highly similar to Calcium-dependent secretion activator 2 | **P** |
|  | [AK000768.1](http://www.ncbi.nlm.nih.gov/UniGene/seq.cgi?ORG=Hs&SID=1970026) | Homo sapiens cDNA FLJ20761 fis, clone HEP00317 | **P** |
|  | [NM_017954.10](http://www.ncbi.nlm.nih.gov/UniGene/seq.cgi?ORG=Hs&SID=2293914) | Homo sapiens Ca++-dependent secretion activator 2 (CADPS2), transcript variant 1, mRNA | **PA** |
|  | [NM_001009571.3](http://www.ncbi.nlm.nih.gov/UniGene/seq.cgi?ORG=Hs&SID=22980697) | Homo sapiens Ca++-dependent secretion activator 2 (CADPS2), transcript variant 2, mRNA | **PA** |
|  | [AB046811.1](http://www.ncbi.nlm.nih.gov/UniGene/seq.cgi?ORG=Hs&SID=2649433) | Homo sapiens mRNA for KIAA1591 protein, partial cds | **P** |
|  | [AK025672.1](http://www.ncbi.nlm.nih.gov/UniGene/seq.cgi?ORG=Hs&SID=2653532) | Homo sapiens cDNA: FLJ22019 fis, clone HEP07982 | **PA** |
|  | [AK054853.1](http://www.ncbi.nlm.nih.gov/UniGene/seq.cgi?ORG=Hs&SID=3991341) | Homo sapiens cDNA FLJ30291 fis, clone BRACE2002896, highly similar to Calcium-dependent secretion activator 2 | **P** |
|  | [AL833058.1](http://www.ncbi.nlm.nih.gov/UniGene/seq.cgi?ORG=Hs&SID=4622334) | Homo sapiens mRNA; cDNA DKFZp666M069 (from clone DKFZp666M069) | **PA** |
|  | [BC144278.1](http://www.ncbi.nlm.nih.gov/UniGene/seq.cgi?ORG=Hs&SID=50138157) | Homo sapiens Ca++-dependent secretion activator 2, mRNA (cDNA clone MGC:177815 IMAGE:9052798), complete cds | **P** |
|  | [BC144279.1](http://www.ncbi.nlm.nih.gov/UniGene/seq.cgi?ORG=Hs&SID=50155401) | Homo sapiens Ca++-dependent secretion activator 2, mRNA (cDNA clone MGC:177816 IMAGE:9052799), complete cds | **P** |
|  | [BC136601.1](http://www.ncbi.nlm.nih.gov/UniGene/seq.cgi?ORG=Hs&SID=50675467) | Homo sapiens Ca++-dependent secretion activator 2, mRNA (cDNA clone MGC:168214 IMAGE:9020591), complete cds | **P** |
|  | [NM_001167940.1](http://www.ncbi.nlm.nih.gov/UniGene/seq.cgi?ORG=Hs&SID=53281105) | Homo sapiens Ca++-dependent secretion activator 2 (CADPS2), transcript variant 3, mRNA | **PA** |
|  | [AF401638.1](http://www.ncbi.nlm.nih.gov/UniGene/seq.cgi?ORG=Hs&SID=5930839) | Homo sapiens CADPS2 mRNA, complete cds | **PA** |

**EST sequences (140)**

|  | [BX110721.1](http://www.ncbi.nlm.nih.gov/UniGene/seq.cgi?ORG=Hs&SID=11133202) | Clone IMAGp998D245769_;_IMAGE:2325863 | intestine |  |  |
| --- | --- | --- | --- | --- | --- |
|  | [BX113217.1](http://www.ncbi.nlm.nih.gov/UniGene/seq.cgi?ORG=Hs&SID=11134828) | Clone IMAGp998O245081_;_IMAGE:2063087 | intestine |  | **PA** |
|  | [AI249959.1](http://www.ncbi.nlm.nih.gov/UniGene/seq.cgi?ORG=Hs&SID=1140595) | Clone IMAGE:2004486 | pancreas | 3' read | **A** |
|  | [AI267472.1](http://www.ncbi.nlm.nih.gov/UniGene/seq.cgi?ORG=Hs&SID=1151813) | Clone IMAGE:2035782 | brain |  | **P** |
|  | [CB134237.1](http://www.ncbi.nlm.nih.gov/UniGene/seq.cgi?ORG=Hs&SID=11565922) | Clone B2N807043-1-D10 | brain | 5' read | **P** |
|  | [AI347502.1](http://www.ncbi.nlm.nih.gov/UniGene/seq.cgi?ORG=Hs&SID=1232148) | Clone IMAGE:2063087 | intestine | 3' read | **P** |
|  | [AI393990.1](http://www.ncbi.nlm.nih.gov/UniGene/seq.cgi?ORG=Hs&SID=1265841) | Clone IMAGE:2107972 | uncharacterized tissue | 3' read | **P** |
|  | [AI479217.1](http://www.ncbi.nlm.nih.gov/UniGene/seq.cgi?ORG=Hs&SID=1301787) | Clone IMAGE:2162086 | kidney | 3' read | **A** |
|  | [AI498316.1](http://www.ncbi.nlm.nih.gov/UniGene/seq.cgi?ORG=Hs&SID=1303800) | Clone IMAGE:2160886 | kidney | 3' read | **P** |
|  | [AI521691.1](http://www.ncbi.nlm.nih.gov/UniGene/seq.cgi?ORG=Hs&SID=1310345) | Clone IMAGE:2138437 | kidney | 3' read | **PA** |
|  | [AI768590.1](http://www.ncbi.nlm.nih.gov/UniGene/seq.cgi?ORG=Hs&SID=1449996) | Clone IMAGE:2381615 | kidney | 3' read | **P** |
|  | [CB267162.1](http://www.ncbi.nlm.nih.gov/UniGene/seq.cgi?ORG=Hs&SID=14791659) |  | adipose tissue | 5' read | **P** |
|  | [BX279929.1](http://www.ncbi.nlm.nih.gov/UniGene/seq.cgi?ORG=Hs&SID=14795705) | Clone IMAGp998K074994_;_IMAGE:2029566 | kidney |  |  |
|  | [CD102185.1](http://www.ncbi.nlm.nih.gov/UniGene/seq.cgi?ORG=Hs&SID=15500989) | Clone IMAGE:30371793 | mixed | 5' read | **P** |
|  | [AI970580.1](http://www.ncbi.nlm.nih.gov/UniGene/seq.cgi?ORG=Hs&SID=1573265) | Clone IMAGE:2481319 | lung | 3' read | **P** |
|  | [AI985250.1](http://www.ncbi.nlm.nih.gov/UniGene/seq.cgi?ORG=Hs&SID=1579601) | Clone IMAGE:2496343 | kidney | 3' read | **P** |
|  | [CD356302.1](http://www.ncbi.nlm.nih.gov/UniGene/seq.cgi?ORG=Hs&SID=15830650) | Clone IMAGE:30402635 | vascular | 5' read | **P** |
|  | [AW008278.1](http://www.ncbi.nlm.nih.gov/UniGene/seq.cgi?ORG=Hs&SID=1590855) | Clone IMAGE:2533197 | stomach | 3' read | **A** |
|  | [AW025404.1](http://www.ncbi.nlm.nih.gov/UniGene/seq.cgi?ORG=Hs&SID=1601396) | Clone IMAGE:2527938 | kidney | 3' read |  |
|  | [CD656746.1](http://www.ncbi.nlm.nih.gov/UniGene/seq.cgi?ORG=Hs&SID=16021632) | Clone IMAGE:30419755 | embryonic tissue | 5' read | **P** |
|  | [CD657777.1](http://www.ncbi.nlm.nih.gov/UniGene/seq.cgi?ORG=Hs&SID=16022663) | Clone IMAGE:30422497 | embryonic tissue | 5' read | **P** |
|  | [AW062254.1](http://www.ncbi.nlm.nih.gov/UniGene/seq.cgi?ORG=Hs&SID=1611670) |  | mammary gland |  | **P** |
|  | [AW135873.1](http://www.ncbi.nlm.nih.gov/UniGene/seq.cgi?ORG=Hs&SID=1643513) | Clone IMAGE:2714639 | mixed | 3' read | **P** |
|  | [AW137549.1](http://www.ncbi.nlm.nih.gov/UniGene/seq.cgi?ORG=Hs&SID=1645189) | Clone IMAGE:2716339 | mixed | 3' read | **P** |
|  | [AW138864.1](http://www.ncbi.nlm.nih.gov/UniGene/seq.cgi?ORG=Hs&SID=1646504) | Clone IMAGE:2720186 | mixed | 3' read | **P** |
|  | [AW192329.1](http://www.ncbi.nlm.nih.gov/UniGene/seq.cgi?ORG=Hs&SID=1677443) | Clone IMAGE:2677524 | pancreas | 3' read |  |
|  | [AW513864.1](http://www.ncbi.nlm.nih.gov/UniGene/seq.cgi?ORG=Hs&SID=1818794) | Clone IMAGE:2707391 | uterus | 3' read | **A** |
|  | [CD628805.1](http://www.ncbi.nlm.nih.gov/UniGene/seq.cgi?ORG=Hs&SID=18192256) |  | uncharacterized tissue |  | **P** |
|  | [CD628806.1](http://www.ncbi.nlm.nih.gov/UniGene/seq.cgi?ORG=Hs&SID=18192257) |  | uncharacterized tissue |  | **P** |
|  | [AW780256.1](http://www.ncbi.nlm.nih.gov/UniGene/seq.cgi?ORG=Hs&SID=1869276) | Clone IMAGE:3036918 | intestine | 3' read | **A** |
|  | [CN359919.1](http://www.ncbi.nlm.nih.gov/UniGene/seq.cgi?ORG=Hs&SID=19963547) |  | embryonic tissue | 5' read | **P** |
|  | [BP203194.1](http://www.ncbi.nlm.nih.gov/UniGene/seq.cgi?ORG=Hs&SID=21762054) | Clone CAE03667 | vascular | 5' read | **P** |
|  | [BP237447.1](http://www.ncbi.nlm.nih.gov/UniGene/seq.cgi?ORG=Hs&SID=21776730) | Clone HEP00317 | liver | 5' read | **P** |
|  | [BP237756.1](http://www.ncbi.nlm.nih.gov/UniGene/seq.cgi?ORG=Hs&SID=21777039) | Clone HEP01070 | liver | 5' read | **P** |
|  | [BP239629.1](http://www.ncbi.nlm.nih.gov/UniGene/seq.cgi?ORG=Hs&SID=21778912) | Clone HEP07982 | liver | 5' read | **P** |
|  | [BP236055.1](http://www.ncbi.nlm.nih.gov/UniGene/seq.cgi?ORG=Hs&SID=21806974) | Clone HCR06748 | vascular | 5' read | **P** |
|  | [BP237128.1](http://www.ncbi.nlm.nih.gov/UniGene/seq.cgi?ORG=Hs&SID=21808047) | Clone HCR09285 | vascular | 5' read | **P** |
|  | [BP326322.1](http://www.ncbi.nlm.nih.gov/UniGene/seq.cgi?ORG=Hs&SID=21835627) | Clone PRS02328 | prostate | 5' read | **P** |
|  | [BP333778.1](http://www.ncbi.nlm.nih.gov/UniGene/seq.cgi?ORG=Hs&SID=21873575) | Clone SLV04177 | salivary gland | 5' read | **P** |
|  | [BP375529.1](http://www.ncbi.nlm.nih.gov/UniGene/seq.cgi?ORG=Hs&SID=21930593) | Clone WMC00914 | uterus | 5' read | **P** |
|  | [BP375597.1](http://www.ncbi.nlm.nih.gov/UniGene/seq.cgi?ORG=Hs&SID=21930661) | Clone WMC01106 | uterus | 5' read | **P** |
|  | [BP375757.1](http://www.ncbi.nlm.nih.gov/UniGene/seq.cgi?ORG=Hs&SID=21930821) | Clone WMC01627 | uterus | 5' read | **P** |
|  | [BP376175.1](http://www.ncbi.nlm.nih.gov/UniGene/seq.cgi?ORG=Hs&SID=21931239) | Clone WMC02979 | uterus | 5' read | **P** |
|  | [BP377000.1](http://www.ncbi.nlm.nih.gov/UniGene/seq.cgi?ORG=Hs&SID=21932064) | Clone WMC06041 | uterus | 5' read | **P** |
|  | [BP377472.1](http://www.ncbi.nlm.nih.gov/UniGene/seq.cgi?ORG=Hs&SID=21932536) | Clone WMC07663 | uterus | 5' read | **P** |
|  | [BP378689.1](http://www.ncbi.nlm.nih.gov/UniGene/seq.cgi?ORG=Hs&SID=21933753) | Clone WMD02901 | uterus | 5' read | **P** |
|  | [BP378871.1](http://www.ncbi.nlm.nih.gov/UniGene/seq.cgi?ORG=Hs&SID=21933935) | Clone WMD03716 | uterus | 5' read | **P** |
|  | [BE386106.1](http://www.ncbi.nlm.nih.gov/UniGene/seq.cgi?ORG=Hs&SID=2208182) | Clone IMAGE:3618243 | skin | 5' read | **P** |
|  | [D59605.1](http://www.ncbi.nlm.nih.gov/UniGene/seq.cgi?ORG=Hs&SID=243608) | Clone GEN-049E12 | brain | 3' read | **A** |
|  | [DN989870.1](http://www.ncbi.nlm.nih.gov/UniGene/seq.cgi?ORG=Hs&SID=24645157) | Clone TC113868 | brain | 5' read | **P** |
|  | [DR005297.1](http://www.ncbi.nlm.nih.gov/UniGene/seq.cgi?ORG=Hs&SID=24660200) | Clone TC105527 | prostate | 5' read | **P** |
|  | [BF359200.1](http://www.ncbi.nlm.nih.gov/UniGene/seq.cgi?ORG=Hs&SID=2745152) |  | lung |  | **P** |
|  | [BF370838.1](http://www.ncbi.nlm.nih.gov/UniGene/seq.cgi?ORG=Hs&SID=2756627) |  | prostate |  | **P** |
|  | [BF372600.1](http://www.ncbi.nlm.nih.gov/UniGene/seq.cgi?ORG=Hs&SID=2758389) |  | prostate |  |  |
|  | [BF445566.1](http://www.ncbi.nlm.nih.gov/UniGene/seq.cgi?ORG=Hs&SID=2769440) | Clone IMAGE:3253696 | prostate | 3' read | **A** |
|  | [DN913927.1](http://www.ncbi.nlm.nih.gov/UniGene/seq.cgi?ORG=Hs&SID=27884005) | Clone MCF7_RNA_L_17_N03 | mammary gland |  | **P** |
|  | [DA306005.1](http://www.ncbi.nlm.nih.gov/UniGene/seq.cgi?ORG=Hs&SID=27951897) | Clone BRHIP2022424 | brain | 5' read | **P** |
|  | [DA234030.1](http://www.ncbi.nlm.nih.gov/UniGene/seq.cgi?ORG=Hs&SID=27953904) | Clone BRAWH3030101 | brain | 5' read | **P** |
|  | [DA084332.1](http://www.ncbi.nlm.nih.gov/UniGene/seq.cgi?ORG=Hs&SID=27964058) | Clone BRACE2038407 | brain | 5' read | **P** |
|  | [DA117684.1](http://www.ncbi.nlm.nih.gov/UniGene/seq.cgi?ORG=Hs&SID=27970966) | Clone BRACE3037450 | brain | 5' read | **P** |
|  | [DA281147.1](http://www.ncbi.nlm.nih.gov/UniGene/seq.cgi?ORG=Hs&SID=27992757) | Clone BRCOC2009818 | brain | 5' read | **P** |
|  | [DA118516.1](http://www.ncbi.nlm.nih.gov/UniGene/seq.cgi?ORG=Hs&SID=27995847) | Clone BRACE3038613 | brain | 5' read |  |
|  | [DA118704.1](http://www.ncbi.nlm.nih.gov/UniGene/seq.cgi?ORG=Hs&SID=27997417) | Clone BRACE3038854 | brain | 5' read | **P** |
|  | [DA125412.1](http://www.ncbi.nlm.nih.gov/UniGene/seq.cgi?ORG=Hs&SID=28014869) | Clone BRACE3048122 | brain | 5' read | **P** |
|  | [DA210803.1](http://www.ncbi.nlm.nih.gov/UniGene/seq.cgi?ORG=Hs&SID=28031902) | Clone BRAWH2019028 | brain | 5' read | **P** |
|  | [DA210992.1](http://www.ncbi.nlm.nih.gov/UniGene/seq.cgi?ORG=Hs&SID=28032968) | Clone BRAWH3000084 | brain | 5' read | **P** |
|  | [DA060101.1](http://www.ncbi.nlm.nih.gov/UniGene/seq.cgi?ORG=Hs&SID=28033747) | Clone BRACE2008303 | brain | 5' read | **P** |
|  | [DA093704.1](http://www.ncbi.nlm.nih.gov/UniGene/seq.cgi?ORG=Hs&SID=28035138) | Clone BRACE3002672 | brain | 5' read |  |
|  | [DA054557.1](http://www.ncbi.nlm.nih.gov/UniGene/seq.cgi?ORG=Hs&SID=28056919) | Clone BRACE2000381 | brain | 5' read | **P** |
|  | [DA323097.1](http://www.ncbi.nlm.nih.gov/UniGene/seq.cgi?ORG=Hs&SID=28082221) | Clone BRHIP3017022 | brain | 5' read | **P** |
|  | [DA323271.1](http://www.ncbi.nlm.nih.gov/UniGene/seq.cgi?ORG=Hs&SID=28083595) | Clone BRHIP3017285 | brain | 5' read | **P** |
|  | [DA317328.1](http://www.ncbi.nlm.nih.gov/UniGene/seq.cgi?ORG=Hs&SID=28088407) | Clone BRHIP3008526 | brain | 5' read | **P** |
|  | [DA056588.1](http://www.ncbi.nlm.nih.gov/UniGene/seq.cgi?ORG=Hs&SID=28105654) | Clone BRACE2002896 | brain | 5' read | **P** |
|  | [DA247593.1](http://www.ncbi.nlm.nih.gov/UniGene/seq.cgi?ORG=Hs&SID=28141110) | Clone BRAWH3046832 | brain | 5' read | **P** |
|  | [DA097256.1](http://www.ncbi.nlm.nih.gov/UniGene/seq.cgi?ORG=Hs&SID=28145252) | Clone BRACE3007779 | brain | 5' read | **P** |
|  | [DA248537.1](http://www.ncbi.nlm.nih.gov/UniGene/seq.cgi?ORG=Hs&SID=28164111) | Clone BRAWH3048013 | brain | 5' read | **P** |
|  | [DA248542.1](http://www.ncbi.nlm.nih.gov/UniGene/seq.cgi?ORG=Hs&SID=28164136) | Clone BRAWH3048018 | brain | 5' read | **P** |
|  | [DA110623.1](http://www.ncbi.nlm.nih.gov/UniGene/seq.cgi?ORG=Hs&SID=28170998) | Clone BRACE3027708 | brain | 5' read | **P** |
|  | [DA098753.1](http://www.ncbi.nlm.nih.gov/UniGene/seq.cgi?ORG=Hs&SID=28171265) | Clone BRACE3009871 | brain | 5' read | **P** |
|  | [DA104707.1](http://www.ncbi.nlm.nih.gov/UniGene/seq.cgi?ORG=Hs&SID=28176086) | Clone BRACE3018219 | brain | 5' read | **P** |
|  | [DA099282.1](http://www.ncbi.nlm.nih.gov/UniGene/seq.cgi?ORG=Hs&SID=28196845) | Clone BRACE3010649 | brain | 5' read | **P** |
|  | [DA112953.1](http://www.ncbi.nlm.nih.gov/UniGene/seq.cgi?ORG=Hs&SID=28225890) | Clone BRACE3031022 | brain | 5' read | **P** |
|  | [DA041479.1](http://www.ncbi.nlm.nih.gov/UniGene/seq.cgi?ORG=Hs&SID=28247858) | Clone BLADE2002911 | bladder | 5' read | **P** |
|  | [DA113888.1](http://www.ncbi.nlm.nih.gov/UniGene/seq.cgi?ORG=Hs&SID=28249483) | Clone BRACE3032285 | brain | 5' read | **P** |
|  | [DA107638.1](http://www.ncbi.nlm.nih.gov/UniGene/seq.cgi?ORG=Hs&SID=28252574) | Clone BRACE3022465 | brain | 5' read | **P** |
|  | [DA076700.1](http://www.ncbi.nlm.nih.gov/UniGene/seq.cgi?ORG=Hs&SID=28296408) | Clone BRACE2028859 | brain | 5' read | **P** |
|  | [DA272214.1](http://www.ncbi.nlm.nih.gov/UniGene/seq.cgi?ORG=Hs&SID=28298302) | Clone BRCAN2026994 | brain | 5' read | **P** |
|  | [DA578599.1](http://www.ncbi.nlm.nih.gov/UniGene/seq.cgi?ORG=Hs&SID=28422833) | Clone HHDPC2006656 | skin | 5' read | **P** |
|  | [DA633311.1](http://www.ncbi.nlm.nih.gov/UniGene/seq.cgi?ORG=Hs&SID=28512399) | Clone KIDNE2015020 | kidney | 5' read | **P** |
|  | [AW611978.1](http://www.ncbi.nlm.nih.gov/UniGene/seq.cgi?ORG=Hs&SID=2857895) | Clone IMAGE:2952155 | kidney | 3' read | **A** |
|  | [BF062462.1](http://www.ncbi.nlm.nih.gov/UniGene/seq.cgi?ORG=Hs&SID=2874143) | Clone IMAGE:3320282 | intestine | 3' read | **P** |
|  | [DA598643.1](http://www.ncbi.nlm.nih.gov/UniGene/seq.cgi?ORG=Hs&SID=28870514) | Clone HSYRA2003290 | connective tissue | 5' read | **P** |
|  | [BF439127.1](http://www.ncbi.nlm.nih.gov/UniGene/seq.cgi?ORG=Hs&SID=2912521) | Clone IMAGE:3270313 | mixed | 3' read | **A** |
|  | [DA940267.1](http://www.ncbi.nlm.nih.gov/UniGene/seq.cgi?ORG=Hs&SID=29145714) | Clone SPLEN2014131 | spleen | 5' read | **P** |
|  | [DA751308.1](http://www.ncbi.nlm.nih.gov/UniGene/seq.cgi?ORG=Hs&SID=29162065) | Clone NT2RP7017154 | testis | 5' read | **P** |
|  | [DA903296.1](http://www.ncbi.nlm.nih.gov/UniGene/seq.cgi?ORG=Hs&SID=29201743) | Clone SKNMC2003403 | brain | 5' read | **P** |
|  | [BF507979.1](http://www.ncbi.nlm.nih.gov/UniGene/seq.cgi?ORG=Hs&SID=2924442) | Clone IMAGE:3088872 | uncharacterized tissue | 3' read | **A** |
|  | [BF594330.1](http://www.ncbi.nlm.nih.gov/UniGene/seq.cgi?ORG=Hs&SID=2948283) | Clone IMAGE:3322233 | intestine | 3' read | **P** |
|  | [BF790766.1](http://www.ncbi.nlm.nih.gov/UniGene/seq.cgi?ORG=Hs&SID=2949786) | Clone IMAGE:4337622 | muscle | 5' read | **P** |
|  | [DB296640.1](http://www.ncbi.nlm.nih.gov/UniGene/seq.cgi?ORG=Hs&SID=29531740) | Clone BRACE2002896 | brain | 3' read | **A** |
|  | [DB219522.1](http://www.ncbi.nlm.nih.gov/UniGene/seq.cgi?ORG=Hs&SID=29567635) | Clone TRACH3009596 | trachea | 5' read | **P** |
|  | [DB352101.1](http://www.ncbi.nlm.nih.gov/UniGene/seq.cgi?ORG=Hs&SID=29637458) | Clone UTERU2037734 | uterus | 3' read | **A** |
|  | [DB280514.1](http://www.ncbi.nlm.nih.gov/UniGene/seq.cgi?ORG=Hs&SID=29641846) | Clone UTERU3007196 | uterus | 5' read | **P** |
|  | [DB280686.1](http://www.ncbi.nlm.nih.gov/UniGene/seq.cgi?ORG=Hs&SID=29642898) | Clone UTERU3007419 | uterus | 5' read | **P** |
|  | [DB274685.1](http://www.ncbi.nlm.nih.gov/UniGene/seq.cgi?ORG=Hs&SID=29646189) | Clone UTERU2037838 | uterus | 5' read | **P** |
|  | [DB281768.1](http://www.ncbi.nlm.nih.gov/UniGene/seq.cgi?ORG=Hs&SID=29665254) | Clone UTERU3008820 | uterus | 5' read | **P** |
|  | [DB158478.1](http://www.ncbi.nlm.nih.gov/UniGene/seq.cgi?ORG=Hs&SID=29679262) | Clone THYMU3037900 | thymus | 5' read | **P** |
|  | [DB203932.1](http://www.ncbi.nlm.nih.gov/UniGene/seq.cgi?ORG=Hs&SID=29680148) | Clone TRACH2014997 | trachea | 5' read | **P** |
|  | [DB282834.1](http://www.ncbi.nlm.nih.gov/UniGene/seq.cgi?ORG=Hs&SID=29687832) | Clone UTERU3010219 | uterus | 5' read | **P** |
|  | [DB290797.1](http://www.ncbi.nlm.nih.gov/UniGene/seq.cgi?ORG=Hs&SID=29764423) | Clone UTERU3020926 | uterus | 5' read | **P** |
|  | [DB278665.1](http://www.ncbi.nlm.nih.gov/UniGene/seq.cgi?ORG=Hs&SID=29770242) | Clone UTERU3004762 | uterus | 5' read | **P** |
|  | [DB239243.1](http://www.ncbi.nlm.nih.gov/UniGene/seq.cgi?ORG=Hs&SID=29773421) | Clone TRACH3035413 | trachea | 5' read | **P** |
|  | [DB247988.1](http://www.ncbi.nlm.nih.gov/UniGene/seq.cgi?ORG=Hs&SID=29821025) | Clone UTERU2000727 | uterus | 5' read | **P** |
|  | [DB214666.1](http://www.ncbi.nlm.nih.gov/UniGene/seq.cgi?ORG=Hs&SID=29821849) | Clone TRACH3003110 | trachea | 5' read | **P** |
|  | [DB214970.1](http://www.ncbi.nlm.nih.gov/UniGene/seq.cgi?ORG=Hs&SID=29824257) | Clone TRACH3003509 | trachea | 5' read | **P** |
|  | [DB287300.1](http://www.ncbi.nlm.nih.gov/UniGene/seq.cgi?ORG=Hs&SID=29834587) | Clone UTERU3016200 | uterus | 5' read | **P** |
|  | [DB289517.1](http://www.ncbi.nlm.nih.gov/UniGene/seq.cgi?ORG=Hs&SID=29882460) | Clone UTERU3019190 | uterus | 5' read | **P** |
|  | [DB501160.1](http://www.ncbi.nlm.nih.gov/UniGene/seq.cgi?ORG=Hs&SID=32146554) | Clone H033079G22 | brain | 5' read | **P** |
|  | [DB505914.2](http://www.ncbi.nlm.nih.gov/UniGene/seq.cgi?ORG=Hs&SID=32186961) | Clone H033097A17 | brain | 5' read | **P** |
|  | [DB572346.2](http://www.ncbi.nlm.nih.gov/UniGene/seq.cgi?ORG=Hs&SID=32201465) | Clone H033079G22 | brain | 3' read |  |
|  | [DB496862.2](http://www.ncbi.nlm.nih.gov/UniGene/seq.cgi?ORG=Hs&SID=32476319) | Clone H033063I20 | brain | 5' read | **P** |
|  | [BG433909.1](http://www.ncbi.nlm.nih.gov/UniGene/seq.cgi?ORG=Hs&SID=3370722) | Clone IMAGE:4611684 | kidney | 5' read |  |
|  | [DT217483.1](http://www.ncbi.nlm.nih.gov/UniGene/seq.cgi?ORG=Hs&SID=34490616) |  | brain |  | **P** |
|  | [DT217866.1](http://www.ncbi.nlm.nih.gov/UniGene/seq.cgi?ORG=Hs&SID=34490999) |  | brain |  | **P** |
|  | [DT220064.1](http://www.ncbi.nlm.nih.gov/UniGene/seq.cgi?ORG=Hs&SID=34493197) |  | brain |  | **P** |
|  | [BG188548.1](http://www.ncbi.nlm.nih.gov/UniGene/seq.cgi?ORG=Hs&SID=3506282) |  | connective tissue |  |  |
|  | [BG203017.1](http://www.ncbi.nlm.nih.gov/UniGene/seq.cgi?ORG=Hs&SID=3520751) |  | connective tissue |  | **P** |
|  | [BG218875.1](http://www.ncbi.nlm.nih.gov/UniGene/seq.cgi?ORG=Hs&SID=3536585) |  | connective tissue |  | **A** |
|  | [W52673.1](http://www.ncbi.nlm.nih.gov/UniGene/seq.cgi?ORG=Hs&SID=414506) | Clone IMAGE:338443 | pancreas | 5' read | **P** |
|  | [W52674.1](http://www.ncbi.nlm.nih.gov/UniGene/seq.cgi?ORG=Hs&SID=414507) | Clone IMAGE:338443 | pancreas | 3' read | **P** |
|  | [ES316435.1](http://www.ncbi.nlm.nih.gov/UniGene/seq.cgi?ORG=Hs&SID=41544214) |  | skin |  | **P** |
|  | [BQ350661.1](http://www.ncbi.nlm.nih.gov/UniGene/seq.cgi?ORG=Hs&SID=4490508) |  | thyroid |  |  |
|  | [BQ720279.1](http://www.ncbi.nlm.nih.gov/UniGene/seq.cgi?ORG=Hs&SID=4609096) | Clone IMAGE:6196622 | nerve | 5' read | **P** |
|  | [BU171473.1](http://www.ncbi.nlm.nih.gov/UniGene/seq.cgi?ORG=Hs&SID=4751626) | Clone IMAGE:6150934 | skin | 5' read | **PA** |
|  | [FN137829.1](http://www.ncbi.nlm.nih.gov/UniGene/seq.cgi?ORG=Hs&SID=52917281) | Clone 215159_3048_2388 |  |  |  |
|  | [FN133863.1](http://www.ncbi.nlm.nih.gov/UniGene/seq.cgi?ORG=Hs&SID=52922289) | Clone 205630_1137_3024 |  |  |  |
|  | [FN049059.1](http://www.ncbi.nlm.nih.gov/UniGene/seq.cgi?ORG=Hs&SID=53032167) | Clone 005405_2689_2246 |  |  |  |
|  | [CA314622.1](http://www.ncbi.nlm.nih.gov/UniGene/seq.cgi?ORG=Hs&SID=6104182) | Clone UI-CF-FN0-afh-d-17-0-UI | lung | 3' read | **A** |
|  | [CA417562.1](http://www.ncbi.nlm.nih.gov/UniGene/seq.cgi?ORG=Hs&SID=6122880) | Clone UI-H-FE0-bbw-i-02-0-UI | bone | 3' read | **PA** |
|  | [AA776134.1](http://www.ncbi.nlm.nih.gov/UniGene/seq.cgi?ORG=Hs&SID=928573) | Clone IMAGE:970968 | brain | 3' read |  |

# EST Profile：

[Hs.740589](http://www.ncbi.nlm.nih.gov/UniGene/clust.cgi?ORG=Hs&CID=740589) - CADPS2: Ca++-dependent secretion activator 2

Breakdown by Body Sites

|  | [Hs.740589](http://www.ncbi.nlm.nih.gov/UniGene/clust.cgi?ORG=Hs&CID=740589) | | | | |
| --- | --- | --- | --- | --- | --- |
| adipose tissue | 77 | 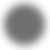 | 1 | / | 12865 |
| bladder | 33 | 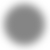 | 1 | / | 29856 |
| bone | 13 | 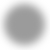 | 1 | / | 71609 |
| brain | 40 | 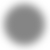 | 44 | / | 1092524 |
| connective tissue | 26 | 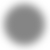 | 4 | / | 149048 |
| embryonic tissue | 14 | 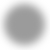 | 3 | / | 212847 |
| intestine | 25 | 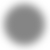 | 6 | / | 232030 |
| kidney | 47 | 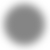 | 10 | / | 210738 |
| liver | 14 | 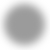 | 3 | / | 205232 |
| lung | 8 | 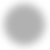 | 3 | / | 334751 |
| mammary gland | 13 | 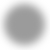 | 2 | / | 151228 |
| muscle | 9 | 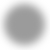 | 1 | / | 106323 |
| nerve | 64 | 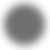 | 1 | / | 15526 |
| pancreas | 18 | 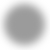 | 4 | / | 213410 |
| prostate | 26 | 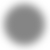 | 5 | / | 189585 |
| salivary gland | 49 | 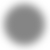 | 1 | / | 20264 |
| skin | 18 | 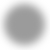 | 4 | / | 210718 |
| spleen | 18 | 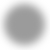 | 1 | / | 53365 |
| stomach | 10 | 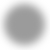 | 1 | / | 95775 |
| testis | 3 | 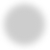 | 1 | / | 327305 |
| thymus | 12 | 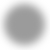 | 1 | / | 79668 |
| thyroid | 21 | 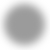 | 1 | / | 46584 |
| trachea | 96 | 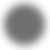 | 5 | / | 51769 |
| uterus | 86 | 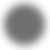 | 20 | / | 232051 |
| vascular | 77 | 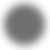 | 4 | / | 51637 |
|  |  |  |  |  |  |
|  |  |  |  |  |  |

Breakdown by Health State

|  | [Hs.740589](http://www.ncbi.nlm.nih.gov/UniGene/clust.cgi?ORG=Hs&CID=740589) | | | | |
| --- | --- | --- | --- | --- | --- |
| breast (mammary gland) tumor | 10 | 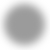 | 1 | / | 93099 |
| chondrosarcoma | 12 | 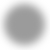 | 1 | / | 82833 |
| colorectal tumor | 8 | 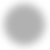 | 1 | / | 112575 |
| gastrointestinal tumor | 8 | 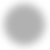 | 1 | / | 118588 |
| germ cell tumor | 3 | 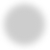 | 1 | / | 263166 |
| head and neck tumor | 7 | 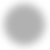 | 1 | / | 133890 |
| leukemia | 10 | 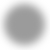 | 1 | / | 94475 |
| liver tumor | 31 | 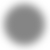 | 3 | / | 96004 |
| lung tumor | 9 | 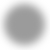 | 1 | / | 102699 |
| non-neoplasia | 20 | 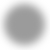 | 2 | / | 96589 |
| normal | 21 | 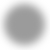 | 72 | / | 3328058 |
| pancreatic tumor | 19 | 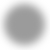 | 2 | / | 104988 |
| primitive neuroectodermal tumor... | 7 | 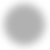 | 1 | / | 126997 |
| prostate cancer | 9 | 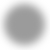 | 1 | / | 103816 |
| skin tumor | 23 | 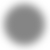 | 3 | / | 125354 |
| soft tissue/muscle tissue tumor | 23 | 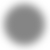 | 3 | / | 125221 |
| uterine tumor | 11 | 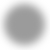 | 1 | / | 90083 |

Breakdown by Developmental Stage

|  | [Hs.740589](http://www.ncbi.nlm.nih.gov/UniGene/clust.cgi?ORG=Hs&CID=740589) | | | | |
| --- | --- | --- | --- | --- | --- |
| blastocyst | 48 | 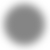 | 3 | / | 61439 |
| fetus | 1 | 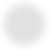 | 1 | / | 556801 |
| juvenile | 89 | 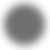 | 5 | / | 55565 |
| adult | 8 | 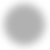 | 17 | / | 1921786 |

**BLAST result：**

| Accession | Description | [Max score](http://blast.ncbi.nlm.nih.gov/Blast.cgi?CMD=Get&ALIGNMENTS=100&ALIGNMENT_VIEW=Pairwise&BLAST_SPEC=OGP__9606__9558&DATABASE_SORT=0&DESCRIPTIONS=100&FIRST_QUERY_NUM=0&FORMAT_OBJECT=Alignment&FORMAT_PAGE_TARGET=&FORMAT_TYPE=HTML&GET_SEQUENCE=yes&I_THRESH=&MASK_CHAR=2&MASK_COLOR=1&NEW_VIEW=yes&NUM_OVERVIEW=100&OLD_BLAST=false&PAGE=Nucleotides&QUERY_INDEX=0&QUERY_NUMBER=0&RESULTS_PAGE_TARGET=&RID=UM97794C012&SHOW_LINKOUT=yes&SHOW_OVERVIEW=yes&STEP_NUMBER=&WORD_SIZE=11&DISPLAY_SORT=1&HSP_SORT=1#sort_mark) | [Total score](http://blast.ncbi.nlm.nih.gov/Blast.cgi?CMD=Get&ALIGNMENTS=100&ALIGNMENT_VIEW=Pairwise&BLAST_SPEC=OGP__9606__9558&DATABASE_SORT=0&DESCRIPTIONS=100&FIRST_QUERY_NUM=0&FORMAT_OBJECT=Alignment&FORMAT_PAGE_TARGET=&FORMAT_TYPE=HTML&GET_SEQUENCE=yes&I_THRESH=&MASK_CHAR=2&MASK_COLOR=1&NEW_VIEW=yes&NUM_OVERVIEW=100&OLD_BLAST=false&PAGE=Nucleotides&QUERY_INDEX=0&QUERY_NUMBER=0&RESULTS_PAGE_TARGET=&RID=UM97794C012&SHOW_LINKOUT=yes&SHOW_OVERVIEW=yes&STEP_NUMBER=&WORD_SIZE=11&DISPLAY_SORT=2&HSP_SORT=1#sort_mark) | [Query coverage](http://blast.ncbi.nlm.nih.gov/Blast.cgi?CMD=Get&ALIGNMENTS=100&ALIGNMENT_VIEW=Pairwise&BLAST_SPEC=OGP__9606__9558&DATABASE_SORT=0&DESCRIPTIONS=100&FIRST_QUERY_NUM=0&FORMAT_OBJECT=Alignment&FORMAT_PAGE_TARGET=&FORMAT_TYPE=HTML&GET_SEQUENCE=yes&I_THRESH=&MASK_CHAR=2&MASK_COLOR=1&NEW_VIEW=yes&NUM_OVERVIEW=100&OLD_BLAST=false&PAGE=Nucleotides&QUERY_INDEX=0&QUERY_NUMBER=0&RESULTS_PAGE_TARGET=&RID=UM97794C012&SHOW_LINKOUT=yes&SHOW_OVERVIEW=yes&STEP_NUMBER=&WORD_SIZE=11&DISPLAY_SORT=4&HSP_SORT=0#sort_mark) | [E value](http://blast.ncbi.nlm.nih.gov/Blast.cgi?CMD=Get&ALIGNMENTS=100&ALIGNMENT_VIEW=Pairwise&BLAST_SPEC=OGP__9606__9558&DATABASE_SORT=0&DESCRIPTIONS=100&FIRST_QUERY_NUM=0&FORMAT_OBJECT=Alignment&FORMAT_PAGE_TARGET=&FORMAT_TYPE=HTML&GET_SEQUENCE=yes&I_THRESH=&MASK_CHAR=2&MASK_COLOR=1&NEW_VIEW=yes&NUM_OVERVIEW=100&OLD_BLAST=false&PAGE=Nucleotides&QUERY_INDEX=0&QUERY_NUMBER=0&RESULTS_PAGE_TARGET=&RID=UM97794C012&SHOW_LINKOUT=yes&SHOW_OVERVIEW=yes&STEP_NUMBER=&WORD_SIZE=11&DISPLAY_SORT=0&HSP_SORT=0#sort_mark) | [Max ident](http://blast.ncbi.nlm.nih.gov/Blast.cgi?CMD=Get&ALIGNMENTS=100&ALIGNMENT_VIEW=Pairwise&BLAST_SPEC=OGP__9606__9558&DATABASE_SORT=0&DESCRIPTIONS=100&FIRST_QUERY_NUM=0&FORMAT_OBJECT=Alignment&FORMAT_PAGE_TARGET=&FORMAT_TYPE=HTML&GET_SEQUENCE=yes&I_THRESH=&MASK_CHAR=2&MASK_COLOR=1&NEW_VIEW=yes&NUM_OVERVIEW=100&OLD_BLAST=false&PAGE=Nucleotides&QUERY_INDEX=0&QUERY_NUMBER=0&RESULTS_PAGE_TARGET=&RID=UM97794C012&SHOW_LINKOUT=yes&SHOW_OVERVIEW=yes&STEP_NUMBER=&WORD_SIZE=11&DISPLAY_SORT=3&HSP_SORT=3#sort_mark) |
| --- | --- | --- | --- | --- | --- | --- |
| [NG_016215.1](http://www.ncbi.nlm.nih.gov/nucleotide/281306722?report=genbank&log$=nucltop&blast_rank=1&RID=UM97794C012) | Homo sapiens Ca++-dependent secretion activator 2 (CADPS2), RefSeqGene on chromosome 7 | [387](http://blast.ncbi.nlm.nih.gov/Blast.cgi#281306722) | 387 | 92% | 7e-105 | 99% |
| [NG_012252.1](http://www.ncbi.nlm.nih.gov/nucleotide/239735601?report=genbank&log$=nucltop&blast_rank=13&RID=UM97794C012) | Homo sapiens glioma tumor suppressor candidate region gene 2 pseudogene (LOC440311) on chromosome 15 | [46.4](http://blast.ncbi.nlm.nih.gov/Blast.cgi#239735601) | 46.4 | 18% | 0.028 | 85% |
| [NG_006020.3](http://www.ncbi.nlm.nih.gov/nucleotide/223718268?report=genbank&log$=nucltop&blast_rank=30&RID=UM97794C012) | Homo sapiens serpin peptidase inhibitor, clade H1, pseudogene 1 (SERPINH1P1) on chromosome 9 | [44.6](http://blast.ncbi.nlm.nih.gov/Blast.cgi#223718268) | 44.6 | 19% | 0.099 | 81% |
| [NG_021467.1](http://www.ncbi.nlm.nih.gov/nucleotide/298286535?report=genbank&log$=nucltop&blast_rank=35&RID=UM97794C012) | Homo sapiens WWC family member 3 (WWC3), RefSeqGene on chromosome X | [42.8](http://blast.ncbi.nlm.nih.gov/Blast.cgi#298286535) | 42.8 | 11% | 0.34 | 93% |
| [NG_031926.1](http://www.ncbi.nlm.nih.gov/nucleotide/363543327?report=genbank&log$=nucltop&blast_rank=57&RID=UM97794C012) | Homo sapiens inositol monophosphatase domain containing 1 (IMPAD1), RefSeqGene on chromosome 8 | [41.0](http://blast.ncbi.nlm.nih.gov/Blast.cgi#363543327) | 41.0 | 12% | 1.2 | 90% |
| [NG_011723.1](http://www.ncbi.nlm.nih.gov/nucleotide/225735632?report=genbank&log$=nucltop&blast_rank=78&RID=UM97794C012) | Homo sapiens zinc finger protein, multitype 2 (ZFPM2), RefSeqGene on chromosome 8 | [39.2](http://blast.ncbi.nlm.nih.gov/Blast.cgi#225735632) | 39.2 | 12% | 4.2 | 88% |
| [NG_007261.1](http://www.ncbi.nlm.nih.gov/nucleotide/163965385?report=genbank&log$=nucltop&blast_rank=79&RID=UM97794C012) | Homo sapiens MRE11 meiotic recombination 11 homolog A (S. cerevisiae) (MRE11A), RefSeqGene (LRG_85) on chromosome 11 | [39.2](http://blast.ncbi.nlm.nih.gov/Blast.cgi#163965385) | 39.2 | 15% | 4.2 | 83% |
