# Supplementary material for: Systematic Analysis of the Gene Expression in the Livers of Nonalcoholic Steatohepatitis: Implications on Potential Biomarkers and Molecular Pathological Mechanism
Source: PLoS One. 2012 Dec 26;7(12):e51131. doi: 10.1371/journal.pone.0051131 (PMC3530598; doi:10.1371/journal.pone.0051131)
Supplement: Table S35 — Information of top five ESTs after rank aggregation in microarray two. (DOC) [file pone.0051131.s037.doc]

1. **AI200985：**

[Transcriptional adaptor 1](http://www.ncbi.nlm.nih.gov/UniGene/clust.cgi?UGID=231004&TAXID=9606&SEARCH=AI200985)

TADA1, Homo sapiens

Hs.435967: 278 sequences.

[Order cDNA clone](http://www.ncbi.nlm.nih.gov/genome/clone/orderclone.cgi?db=unigene&uid=231004)

**Transcriptional adaptor 1 (TADA1)**

**SELECTED PROTEIN SIMILARITIES**

Comparison of cluster transcripts with RefSeq proteins. The alignments can suggest function of the cluster.

| **Best Hits and Hits from model organisms** | | **Species** | **Id(%)** | **Len(aa)** |
| --- | --- | --- | --- | --- |
| [XP_001087291.1](javascript:PopUpMenu2_Set(Menu_prot109019352);) | PREDICTED: transcriptional adapter 1 | *M. mulatta* | 100.0 | 334 |
| [XP_513967.1](javascript:PopUpMenu2_Set(Menu_prot55588678);) | PREDICTED: transcriptional adapter 1 | *P. troglodytes* | 100.0 | 334 |
| [NP_444281.1](javascript:PopUpMenu2_Set(Menu_prot16596696);) | transcriptional adapter 1 | *H. sapiens* | 100.0 | 334 |
| [XP_001925145.1](javascript:PopUpMenu2_Set(Menu_prot194036823);) | PREDICTED: transcriptional adapter 1 | *S. scrofa* | 99.7 | 334 |
| [NP_084521.1](javascript:PopUpMenu2_Set(Menu_prot21313610);) | transcriptional adapter 1 | *M. musculus* | 98.5 | 334 |
| [NP_001086038.1](javascript:PopUpMenu2_Set(Menu_prot147900764);) | transcriptional adapter 1 | *X. laevis* | 87.6 | 330 |
| [NP_991169.1](javascript:PopUpMenu2_Set(Menu_prot45387639);) | transcriptional adapter 1 | *D. rerio* | 74.6 | 331 |
| **Other hits (15) [**[**Show subset**](http://www.ncbi.nlm.nih.gov/UniGene/clust.cgi?ORG=Hs&CID=435967)**]** | | **Species** | **Id(%)** | **Len(aa)** |
| [XP_537213.2](javascript:PopUpMenu2_Set(Menu_prot73961417);) | PREDICTED: transcriptional adapter 1 | *C. lupus familiaris* | 99.4 | 334 |
| [XP_002715846.1](javascript:PopUpMenu2_Set(Menu_prot291397494);) | PREDICTED: transcriptional adaptor 1-like | *O. cuniculus* | 99.4 | 334 |
| [NP_001033069.2](javascript:PopUpMenu2_Set(Menu_prot189181738);) | transcriptional adapter 1 | *R. norvegicus* | 98.8 | 334 |
| [NP_001094576.1](javascript:PopUpMenu2_Set(Menu_prot155371943);) | transcriptional adapter 1 | *B. taurus* | 98.8 | 334 |
| [XP_001490179.2](javascript:PopUpMenu2_Set(Menu_prot194210260);) | PREDICTED: transcriptional adapter 1-like | *E. caballus* | 98.2 | 334 |
| [XP_001363942.1](javascript:PopUpMenu2_Set(Menu_prot126306190);) | PREDICTED: transcriptional adapter 1-like | *M. domestica* | 97.3 | 334 |
| [XP_001514979.2](javascript:PopUpMenu2_Set(Menu_prot345310158);) | PREDICTED: transcriptional adapter 1-like, partial | *O. anatinus* | 95.8 | 119 |
| [XP_003208643.1](javascript:PopUpMenu2_Set(Menu_prot326924862);) | PREDICTED: transcriptional adapter 1-like | *M. gallopavo* | 94.7 | 280 |
| [XP_002193964.1](javascript:PopUpMenu2_Set(Menu_prot224058827);) | PREDICTED: similar to SPT3-associated factor 42 | *T. guttata* | 94.6 | 334 |
| [NP_001183965.1](javascript:PopUpMenu2_Set(Menu_prot334358894);) | transcriptional adaptor 1 (HFI1 homolog, yeast)-like | *G. gallus* | 94.3 | 334 |
| [XP_003219903.1](javascript:PopUpMenu2_Set(Menu_prot327270251);) | PREDICTED: transcriptional adapter 1-like | *A. carolinensis* | 91.0 | 332 |
| [NP_001005070.1](javascript:PopUpMenu2_Set(Menu_prot52346046);) | transcriptional adapter 1 | *X. tropicalis* | 85.8 | 330 |
| [XP_003453374.1](javascript:PopUpMenu2_Set(Menu_prot348531756);) | PREDICTED: transcriptional adapter 1-like | *O. niloticus* | 75.6 | 332 |
| [XP_002735386.1](javascript:PopUpMenu2_Set(Menu_prot291230858);) | PREDICTED: transcriptional adaptor 1-like | *S. kowalevskii* | 57.2 | 325 |
| [XP_002590845.1](javascript:PopUpMenu2_Set(Menu_prot260791657);) | hypothetical protein BRAFLDRAFT_125713 | *B. floridae* | 54.6 | 337 |

**GENE EXPRESSION**

Tissues and development stages from this gene's sequences survey gene expression. Links to other NCBI expression resources.

|  | [EST Profile](http://www.ncbi.nlm.nih.gov/UniGene/ESTProfileViewer.cgi?uglist=Hs.435967): | Approximate expression patterns inferred from EST sources. [[Show more entries with profiles like this](http://www.ncbi.nlm.nih.gov/sites/entrez?DB=unigene&DbFrom=unigene&IdsFromResult=231004&cmd=Link&LinkName=unigene_unigene_expression&tool=UniGene.clust)] |
| --- | --- | --- |
|  | [GEO Profiles](http://www.ncbi.nlm.nih.gov/sites/entrez?DB=geoprofiles&DbFrom=unigene&IdsFromResult=231004&cmd=Link&LinkName=unigene_geoprofiles&tool=UniGene.clust): | Experimental gene expression data (Gene Expression Omnibus). |
|  | cDNA Sources: | brain; testis; mixed; liver; bone marrow; pharynx; uncharacterized tissue; placenta; mouth; embryonic tissue; uterus; mammary gland; thyroid; kidney; thymus; stomach; lung; intestine; skin; esophagus; blood; cervix; adrenal gland; spleen; vascular; muscle; trachea; salivary gland; prostate; eye; parathyroid; pancreas; heart; connective tissue; umbilical cord; bone |

**MAPPING POSITION**

Genomic location specified by transcript mapping, radiation hybrid mapping, genetic mapping or cytogenetic mapping.

|  | Chromosome: | 1 |  | |
| --- | --- | --- | --- | --- |
|  | Map position: | 1q24.1 |  | |
|  | UniSTS entry: | Chr 8 | [D3S2967E](http://www.ncbi.nlm.nih.gov/genome/sts/sts.cgi?uid=150951) |  |
|  | UniSTS entry: | Chr 1 | [SGC34611](http://www.ncbi.nlm.nih.gov/genome/sts/sts.cgi?uid=75966) | [[Map Viewer](http://www.ncbi.nlm.nih.gov/mapview/maps.cgi?taxid=9606&chr=1&MAPS=wirh-r,ncbirh-r,gb4-r&sts=75966)] |
|  | UniSTS entry: | Chr 1 | [RH16504](http://www.ncbi.nlm.nih.gov/genome/sts/sts.cgi?uid=22693) |  |
|  | UniSTS entry: |  | [SHGC-57163](http://www.ncbi.nlm.nih.gov/genome/sts/sts.cgi?uid=73173) |  |
|  | UniSTS entry: |  | [RH8147](http://www.ncbi.nlm.nih.gov/genome/sts/sts.cgi?uid=84504) |  |
|  |  |  |  |  |

**SEQUENCES**

*Sequences representing this gene; mRNAs, ESTs, and gene predictions supported by transcribed sequences.*

**mRNA sequences (8)**

|  | [BC015401.2](http://www.ncbi.nlm.nih.gov/UniGene/seq.cgi?ORG=Hs&SID=3940478) | Homo sapiens transcriptional adaptor 1 (HFI1 homolog, yeast)-like, mRNA (cDNA clone MGC:21805 IMAGE:4182025), complete cds | **PA^1^** |
| --- | --- | --- | --- |
|  | [NM_053053.3](http://www.ncbi.nlm.nih.gov/UniGene/seq.cgi?ORG=Hs&SID=3988011) | Homo sapiens transcriptional adaptor 1 (TADA1), mRNA | **PA** |
|  | [AK291922.1](http://www.ncbi.nlm.nih.gov/UniGene/seq.cgi?ORG=Hs&SID=41443632) | Homo sapiens cDNA FLJ75093 complete cds, highly similar to Homo sapiens transcriptional adaptor 1-like (TADA1L), mRNA | **P** |
|  | [AK290964.1](http://www.ncbi.nlm.nih.gov/UniGene/seq.cgi?ORG=Hs&SID=41444588) | Homo sapiens cDNA FLJ77638 complete cds, highly similar to Homo sapiens SPT3-associated factor 42 (STAF42), mRNA | **P** |
|  | [AK309250.1](http://www.ncbi.nlm.nih.gov/UniGene/seq.cgi?ORG=Hs&SID=42529580) | Homo sapiens cDNA, FLJ99291 | **P** |
|  | [AK310391.1](http://www.ncbi.nlm.nih.gov/UniGene/seq.cgi?ORG=Hs&SID=42535371) | Homo sapiens cDNA, FLJ17433 | **P** |
|  | [AK097234.1](http://www.ncbi.nlm.nih.gov/UniGene/seq.cgi?ORG=Hs&SID=4614229) | Homo sapiens cDNA FLJ39915 fis, clone SPLEN2018933 |  |
|  | [BC036497.1](http://www.ncbi.nlm.nih.gov/UniGene/seq.cgi?ORG=Hs&SID=5517921) | Homo sapiens transcriptional adaptor 1 (HFI1 homolog, yeast)-like, mRNA (cDNA clone IMAGE:5264296), with apparent retained intron | **PA** |

**EST sequences (10 of 270) [**[**Show all sequences**](http://www.ncbi.nlm.nih.gov/UniGene/clust.cgi?ORG=Hs&CID=435967&MAXEST=270)**]**

|  | [AI091145.1](http://www.ncbi.nlm.nih.gov/UniGene/seq.cgi?ORG=Hs&SID=1080192) | Clone IMAGE:1689831 | mixed | 3' read | **A** |
| --- | --- | --- | --- | --- | --- |
|  | [AI129857.1](http://www.ncbi.nlm.nih.gov/UniGene/seq.cgi?ORG=Hs&SID=1096675) | Clone IMAGE:1711573 | uterus | 3' read | **A** |
|  | [R23855.1](http://www.ncbi.nlm.nih.gov/UniGene/seq.cgi?ORG=Hs&SID=110160) | Clone IMAGE:132975 | placenta | 5' read | **A** |
|  | [BX090015.1](http://www.ncbi.nlm.nih.gov/UniGene/seq.cgi?ORG=Hs&SID=11150070) | Clone IMAGp998B171196_;_IMAGE:501424 | uterus |  |  |
|  | [AI269749.1](http://www.ncbi.nlm.nih.gov/UniGene/seq.cgi?ORG=Hs&SID=1154090) | Clone IMAGE:1848016 | mixed | 3' read | **A** |
|  | [CB156261.1](http://www.ncbi.nlm.nih.gov/UniGene/seq.cgi?ORG=Hs&SID=11567830) | Clone B2N807043-9-F11 | brain | 5' read | **P** |
|  | [CB151635.1](http://www.ncbi.nlm.nih.gov/UniGene/seq.cgi?ORG=Hs&SID=11570623) | Clone C1SNU17-38-A01 | cervix | 5' read | **P** |
|  | [AI200985.1](http://www.ncbi.nlm.nih.gov/UniGene/seq.cgi?ORG=Hs&SID=1172693) | Clone IMAGE:1754720 | testis | 3' read | **PA** |
|  | [AI241920.1](http://www.ncbi.nlm.nih.gov/UniGene/seq.cgi?ORG=Hs&SID=1179378) | Clone IMAGE:1977666 | brain | 3' read | **A** |
|  | [AI286118.1](http://www.ncbi.nlm.nih.gov/UniGene/seq.cgi?ORG=Hs&SID=1196308) | Clone IMAGE:1855196 | mixed | 3' read | **A** |

^1^: **A** Contains a poly-**A**denylation signal

**P** Has similarity to known **P**roteins (after translation)
**S** Sequence is a **S**uboptimal member of this cluster
**M** Clone is putatively CDS-complete by **M**GC criteria

**EST Profile^2^：**

[Hs.435967](http://www.ncbi.nlm.nih.gov/UniGene/clust.cgi?ORG=Hs&CID=435967) - TADA1: Transcriptional adaptor 1

Breakdown by Body Sites

| adrenal gland | 60 | 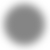 | 2 | / | 32921 |
| --- | --- | --- | --- | --- | --- |
| blood | 32 | 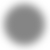 | 4 | / | 122262 |
| bone | 13 | 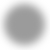 | 1 | / | 71609 |
| bone marrow | 184 | 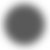 | 9 | / | 48711 |
| brain | 55 | 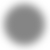 | 61 | / | 1092524 |
| cervix | 82 | 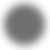 | 4 | / | 48469 |
| connective tissue | 13 | 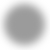 | 2 | / | 149048 |
| embryonic tissue | 51 | 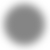 | 11 | / | 212847 |
| esophagus | 49 | 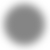 | 1 | / | 20152 |
| eye | 14 | 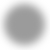 | 3 | / | 208810 |
| heart | 33 | 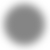 | 3 | / | 89512 |
| intestine | 12 | 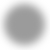 | 3 | / | 232030 |
| kidney | 42 | 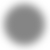 | 9 | / | 210738 |
| liver | 48 | 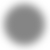 | 10 | / | 205232 |
| lung | 11 | 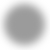 | 4 | / | 334751 |
| mammary gland | 33 | 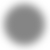 | 5 | / | 151228 |
| mouth | 45 | 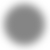 | 3 | / | 66139 |
| muscle | 28 | 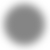 | 3 | / | 106323 |
| pancreas | 18 | 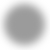 | 4 | / | 213410 |
| parathyroid | 97 | 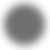 | 2 | / | 20579 |
| pharynx | 73 | 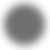 | 3 | / | 40762 |
| placenta | 24 | 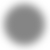 | 7 | / | 282968 |
| prostate | 10 | 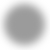 | 2 | / | 189585 |
| salivary gland | 49 | 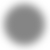 | 1 | / | 20264 |
| skin | 14 | 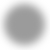 | 3 | / | 210718 |
| spleen | 74 | 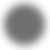 | 4 | / | 53365 |
| stomach | 41 | 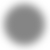 | 4 | / | 95775 |
| testis | 64 | 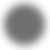 | 21 | / | 327305 |
| thymus | 75 | 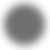 | 6 | / | 79668 |
| thyroid | 64 | 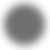 | 3 | / | 46584 |
| trachea | 19 | 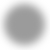 | 1 | / | 51769 |
| umbilical cord | 72 | 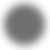 | 1 | / | 13764 |
| uterus | 60 | 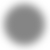 | 14 | / | 232051 |
| vascular | 58 | 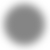 | 3 | / | 51637 |

Breakdown by Health State

| breast (mammary gland) tumor | 32 | 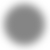 | 3 | / | 93099 |
| --- | --- | --- | --- | --- | --- |
| cervical tumor | 116 | 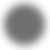 | 4 | / | 34473 |
| chondrosarcoma | 12 | 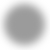 | 1 | / | 82833 |
| colorectal tumor | 8 | 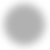 | 1 | / | 112575 |
| esophageal tumor | 57 | 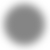 | 1 | / | 17242 |
| gastrointestinal tumor | 16 | 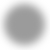 | 2 | / | 118588 |
| germ cell tumor | 60 | 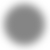 | 16 | / | 263166 |
| glioma | 18 | 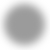 | 2 | / | 107167 |
| head and neck tumor | 37 | 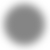 | 5 | / | 133890 |
| kidney tumor | 29 | 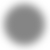 | 2 | / | 68849 |
| leukemia | 10 | 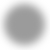 | 1 | / | 94475 |
| liver tumor | 83 | 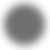 | 8 | / | 96004 |
| non-neoplasia | 10 | 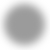 | 1 | / | 96589 |
| normal | 39 | 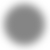 | 131 | / | 3328058 |
| pancreatic tumor | 19 | 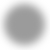 | 2 | / | 104988 |
| primitive neuroectodermal tumor... | 94 | 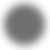 | 12 | / | 126997 |
| prostate cancer | 19 | 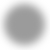 | 2 | / | 103816 |
| retinoblastoma | 64 | 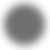 | 3 | / | 46437 |
| skin tumor | 15 | 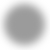 | 2 | / | 125354 |
| soft tissue/muscle tissue tumor | 15 | 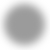 | 2 | / | 125221 |
| uterine tumor | 11 | 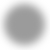 | 1 | / | 90083 |

Breakdown by Developmental Stage

| embryoid body | 42 | 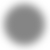 | 3 | / | 69951 |
| --- | --- | --- | --- | --- | --- |
| blastocyst | 97 | 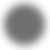 | 6 | / | 61439 |
| fetus | 43 | 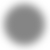 | 24 | / | 556801 |
| neonate | 64 | 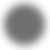 | 2 | / | 31069 |
| juvenile | 71 | 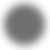 | 4 | / | 55565 |
| adult | 30 | 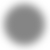 | 59 | / | 1921786 |

**^2^:The EST profile was derived from UniGene EST profile and was filtered so that items with zero transcripts per million(TPM) and zero Gene EST were discarded. The following EST profiles are done in the same way.**

**BLAST result^3^：**

| Accession | Description | [Max score](http://blast.ncbi.nlm.nih.gov/Blast.cgi?CMD=Get&ALIGNMENTS=100&ALIGNMENT_VIEW=Pairwise&BLAST_SPEC=OGP__9606__9558&DATABASE_SORT=0&DESCRIPTIONS=100&FIRST_QUERY_NUM=0&FORMAT_OBJECT=Alignment&FORMAT_PAGE_TARGET=&FORMAT_TYPE=HTML&GET_SEQUENCE=yes&I_THRESH=&MASK_CHAR=2&MASK_COLOR=1&NEW_VIEW=yes&NUM_OVERVIEW=100&OLD_BLAST=false&PAGE=Nucleotides&QUERY_INDEX=0&QUERY_NUMBER=0&RESULTS_PAGE_TARGET=&RID=T5JCPGBY01S&SHOW_LINKOUT=yes&SHOW_OVERVIEW=yes&STEP_NUMBER=&WORD_SIZE=11&DISPLAY_SORT=1&HSP_SORT=1#sort_mark) | [Total score](http://blast.ncbi.nlm.nih.gov/Blast.cgi?CMD=Get&ALIGNMENTS=100&ALIGNMENT_VIEW=Pairwise&BLAST_SPEC=OGP__9606__9558&DATABASE_SORT=0&DESCRIPTIONS=100&FIRST_QUERY_NUM=0&FORMAT_OBJECT=Alignment&FORMAT_PAGE_TARGET=&FORMAT_TYPE=HTML&GET_SEQUENCE=yes&I_THRESH=&MASK_CHAR=2&MASK_COLOR=1&NEW_VIEW=yes&NUM_OVERVIEW=100&OLD_BLAST=false&PAGE=Nucleotides&QUERY_INDEX=0&QUERY_NUMBER=0&RESULTS_PAGE_TARGET=&RID=T5JCPGBY01S&SHOW_LINKOUT=yes&SHOW_OVERVIEW=yes&STEP_NUMBER=&WORD_SIZE=11&DISPLAY_SORT=2&HSP_SORT=1#sort_mark) | [Query coverage](http://blast.ncbi.nlm.nih.gov/Blast.cgi?CMD=Get&ALIGNMENTS=100&ALIGNMENT_VIEW=Pairwise&BLAST_SPEC=OGP__9606__9558&DATABASE_SORT=0&DESCRIPTIONS=100&FIRST_QUERY_NUM=0&FORMAT_OBJECT=Alignment&FORMAT_PAGE_TARGET=&FORMAT_TYPE=HTML&GET_SEQUENCE=yes&I_THRESH=&MASK_CHAR=2&MASK_COLOR=1&NEW_VIEW=yes&NUM_OVERVIEW=100&OLD_BLAST=false&PAGE=Nucleotides&QUERY_INDEX=0&QUERY_NUMBER=0&RESULTS_PAGE_TARGET=&RID=T5JCPGBY01S&SHOW_LINKOUT=yes&SHOW_OVERVIEW=yes&STEP_NUMBER=&WORD_SIZE=11&DISPLAY_SORT=4&HSP_SORT=0#sort_mark) | [E value](http://blast.ncbi.nlm.nih.gov/Blast.cgi?CMD=Get&ALIGNMENTS=100&ALIGNMENT_VIEW=Pairwise&BLAST_SPEC=OGP__9606__9558&DATABASE_SORT=0&DESCRIPTIONS=100&FIRST_QUERY_NUM=0&FORMAT_OBJECT=Alignment&FORMAT_PAGE_TARGET=&FORMAT_TYPE=HTML&GET_SEQUENCE=yes&I_THRESH=&MASK_CHAR=2&MASK_COLOR=1&NEW_VIEW=yes&NUM_OVERVIEW=100&OLD_BLAST=false&PAGE=Nucleotides&QUERY_INDEX=0&QUERY_NUMBER=0&RESULTS_PAGE_TARGET=&RID=T5JCPGBY01S&SHOW_LINKOUT=yes&SHOW_OVERVIEW=yes&STEP_NUMBER=&WORD_SIZE=11&DISPLAY_SORT=0&HSP_SORT=0#sort_mark) | [Max ident](http://blast.ncbi.nlm.nih.gov/Blast.cgi?CMD=Get&ALIGNMENTS=100&ALIGNMENT_VIEW=Pairwise&BLAST_SPEC=OGP__9606__9558&DATABASE_SORT=0&DESCRIPTIONS=100&FIRST_QUERY_NUM=0&FORMAT_OBJECT=Alignment&FORMAT_PAGE_TARGET=&FORMAT_TYPE=HTML&GET_SEQUENCE=yes&I_THRESH=&MASK_CHAR=2&MASK_COLOR=1&NEW_VIEW=yes&NUM_OVERVIEW=100&OLD_BLAST=false&PAGE=Nucleotides&QUERY_INDEX=0&QUERY_NUMBER=0&RESULTS_PAGE_TARGET=&RID=T5JCPGBY01S&SHOW_LINKOUT=yes&SHOW_OVERVIEW=yes&STEP_NUMBER=&WORD_SIZE=11&DISPLAY_SORT=3&HSP_SORT=3#sort_mark) |
| --- | --- | --- | --- | --- | --- | --- |
| [NG_022407.1](http://www.ncbi.nlm.nih.gov/nucleotide/298358290?report=genbank&log$=nucltop&blast_rank=5&RID=T6YW55UA013) | Homo sapiens transcriptional adaptor 1 pseudogene (LOC392180) on chromosome 8 | [223](http://blast.ncbi.nlm.nih.gov/Blast.cgi#298358290) | 223 | 35% | 4e-55 | 89% |
| [NG_009363.1](http://www.ncbi.nlm.nih.gov/nucleotide/221139818?report=genbank&log$=nucltop&blast_rank=10&RID=T6YW55UA013) | Homo sapiens bone morphogenetic protein receptor, type II (serine/threonine kinase) (BMPR2), RefSeqGene on chromosome 2 | [42.8](http://blast.ncbi.nlm.nih.gov/Blast.cgi#221139818) | 42.8 | 5% | 0.75 | 93% |

**^3^：the blast was done in NCBI by blastn, and the species genome is Human. For ‘Search set’ we chose ‘RefSeq Genomic’ and for ‘Program Selection’ we chose ‘Somewhat similar sequences (blastn)’. Other parameters were set default. The following blast results are done in the same way.**

1. **AL117453：**

[MRNA; cDNA DKFZp586G1917 (from clone DKFZp586G1917)](http://www.ncbi.nlm.nih.gov/UniGene/clust.cgi?UGID=2742104&TAXID=9606&SEARCH=AL117453)

Homo sapiens

Hs.672786: 1 sequences.

**MRNA; cDNA DKFZp586G1917 (from clone DKFZp586G1917)**

**GENE EXPRESSION**

Tissues and development stages from this gene's sequences survey gene expression. Links to other NCBI expression resources.

|  | [GEO Profiles](http://www.ncbi.nlm.nih.gov/sites/entrez?DB=geoprofiles&DbFrom=unigene&IdsFromResult=2742104&cmd=Link&LinkName=unigene_geoprofiles&tool=UniGene.clust): | Experimental gene expression data (Gene Expression Omnibus). |
| --- | --- | --- |

**SEQUENCES**

*Sequences representing this gene; mRNAs, ESTs, and gene predictions supported by transcribed sequences.*

**mRNA sequences (1)**

|  | [AL117453.1](http://www.ncbi.nlm.nih.gov/UniGene/seq.cgi?ORG=Hs&SID=1637899) | Homo sapiens mRNA; cDNA DKFZp586G1917 (from clone DKFZp586G1917) |
| --- | --- | --- |

**BLAST result：**

| Accession | Description | [Max score](http://blast.ncbi.nlm.nih.gov/Blast.cgi?CMD=Get&ALIGNMENTS=100&ALIGNMENT_VIEW=Pairwise&BLAST_SPEC=OGP__9606__9558&DATABASE_SORT=0&DESCRIPTIONS=100&FIRST_QUERY_NUM=0&FORMAT_OBJECT=Alignment&FORMAT_PAGE_TARGET=&FORMAT_TYPE=HTML&GET_SEQUENCE=yes&I_THRESH=&MASK_CHAR=2&MASK_COLOR=1&NEW_VIEW=yes&NUM_OVERVIEW=100&OLD_BLAST=false&PAGE=Nucleotides&QUERY_INDEX=0&QUERY_NUMBER=0&RESULTS_PAGE_TARGET=&RID=T5JCPGBY01S&SHOW_LINKOUT=yes&SHOW_OVERVIEW=yes&STEP_NUMBER=&WORD_SIZE=11&DISPLAY_SORT=1&HSP_SORT=1#sort_mark) | [Total score](http://blast.ncbi.nlm.nih.gov/Blast.cgi?CMD=Get&ALIGNMENTS=100&ALIGNMENT_VIEW=Pairwise&BLAST_SPEC=OGP__9606__9558&DATABASE_SORT=0&DESCRIPTIONS=100&FIRST_QUERY_NUM=0&FORMAT_OBJECT=Alignment&FORMAT_PAGE_TARGET=&FORMAT_TYPE=HTML&GET_SEQUENCE=yes&I_THRESH=&MASK_CHAR=2&MASK_COLOR=1&NEW_VIEW=yes&NUM_OVERVIEW=100&OLD_BLAST=false&PAGE=Nucleotides&QUERY_INDEX=0&QUERY_NUMBER=0&RESULTS_PAGE_TARGET=&RID=T5JCPGBY01S&SHOW_LINKOUT=yes&SHOW_OVERVIEW=yes&STEP_NUMBER=&WORD_SIZE=11&DISPLAY_SORT=2&HSP_SORT=1#sort_mark) | [Query coverage](http://blast.ncbi.nlm.nih.gov/Blast.cgi?CMD=Get&ALIGNMENTS=100&ALIGNMENT_VIEW=Pairwise&BLAST_SPEC=OGP__9606__9558&DATABASE_SORT=0&DESCRIPTIONS=100&FIRST_QUERY_NUM=0&FORMAT_OBJECT=Alignment&FORMAT_PAGE_TARGET=&FORMAT_TYPE=HTML&GET_SEQUENCE=yes&I_THRESH=&MASK_CHAR=2&MASK_COLOR=1&NEW_VIEW=yes&NUM_OVERVIEW=100&OLD_BLAST=false&PAGE=Nucleotides&QUERY_INDEX=0&QUERY_NUMBER=0&RESULTS_PAGE_TARGET=&RID=T5JCPGBY01S&SHOW_LINKOUT=yes&SHOW_OVERVIEW=yes&STEP_NUMBER=&WORD_SIZE=11&DISPLAY_SORT=4&HSP_SORT=0#sort_mark) | [E value](http://blast.ncbi.nlm.nih.gov/Blast.cgi?CMD=Get&ALIGNMENTS=100&ALIGNMENT_VIEW=Pairwise&BLAST_SPEC=OGP__9606__9558&DATABASE_SORT=0&DESCRIPTIONS=100&FIRST_QUERY_NUM=0&FORMAT_OBJECT=Alignment&FORMAT_PAGE_TARGET=&FORMAT_TYPE=HTML&GET_SEQUENCE=yes&I_THRESH=&MASK_CHAR=2&MASK_COLOR=1&NEW_VIEW=yes&NUM_OVERVIEW=100&OLD_BLAST=false&PAGE=Nucleotides&QUERY_INDEX=0&QUERY_NUMBER=0&RESULTS_PAGE_TARGET=&RID=T5JCPGBY01S&SHOW_LINKOUT=yes&SHOW_OVERVIEW=yes&STEP_NUMBER=&WORD_SIZE=11&DISPLAY_SORT=0&HSP_SORT=0#sort_mark) | [Max ident](http://blast.ncbi.nlm.nih.gov/Blast.cgi?CMD=Get&ALIGNMENTS=100&ALIGNMENT_VIEW=Pairwise&BLAST_SPEC=OGP__9606__9558&DATABASE_SORT=0&DESCRIPTIONS=100&FIRST_QUERY_NUM=0&FORMAT_OBJECT=Alignment&FORMAT_PAGE_TARGET=&FORMAT_TYPE=HTML&GET_SEQUENCE=yes&I_THRESH=&MASK_CHAR=2&MASK_COLOR=1&NEW_VIEW=yes&NUM_OVERVIEW=100&OLD_BLAST=false&PAGE=Nucleotides&QUERY_INDEX=0&QUERY_NUMBER=0&RESULTS_PAGE_TARGET=&RID=T5JCPGBY01S&SHOW_LINKOUT=yes&SHOW_OVERVIEW=yes&STEP_NUMBER=&WORD_SIZE=11&DISPLAY_SORT=3&HSP_SORT=3#sort_mark) |
| --- | --- | --- | --- | --- | --- | --- |
| [NG_008799.1](http://www.ncbi.nlm.nih.gov/nucleotide/209969780?report=genbank&log$=nucltop&blast_rank=40&RID=T78E6GYH016) | Homo sapiens ryanodine receptor 2 (cardiac) (RYR2), RefSeqGene on chromosome 1 | [480](http://blast.ncbi.nlm.nih.gov/Blast.cgi#209969780) | 480 | 21% | 7e-132 | 75% |
| [NG_029492.1](http://www.ncbi.nlm.nih.gov/nucleotide/340805816?report=genbank&log$=nucltop&blast_rank=59&RID=T78E6GYH016) | Homo sapiens collagen, type IV, alpha 3 (Goodpasture antigen) binding protein (COL4A3BP), RefSeqGene on chromosome 5 | [421](http://blast.ncbi.nlm.nih.gov/Blast.cgi#340805816) | 421 | 19% | 6e-114 | 74% |
| [NG_009780.1](http://www.ncbi.nlm.nih.gov/nucleotide/223671928?report=genbank&log$=nucltop&blast_rank=92&RID=T78E6GYH016) | Homo sapiens dyskeratosis congenita 1, dyskerin (DKC1), RefSeqGene (LRG_55) on chromosome X | [221](http://blast.ncbi.nlm.nih.gov/Blast.cgi#223671928) | 221 | 13% | 1e-53 | 71% |
| [NG_015832.1](http://www.ncbi.nlm.nih.gov/nucleotide/263191878?report=genbank&log$=nucltop&blast_rank=96&RID=T78E6GYH016) | Homo sapiens thyroglobulin (TG), RefSeqGene on chromosome 8 | [217](http://blast.ncbi.nlm.nih.gov/Blast.cgi#263191878) | 217 | 8% | 1e-52 | 79% |

1. **BM832957：**

[Transcribed locus](http://www.ncbi.nlm.nih.gov/UniGene/clust.cgi?UGID=133751&TAXID=9606&SEARCH=BM832957)

Homo sapiens

Hs.17910: 18 sequences.

**Transcribed locus**

**GENE EXPRESSION**

Tissues and development stages from this gene's sequences survey gene expression. Links to other NCBI expression resources.

|  | Restricted Expression: | glioma [[show more like this](http://www.ncbi.nlm.nih.gov/sites/entrez?db=unigene&cmd=search&term=9606%5Btaxid%5D AND  glioma%5Brestricted%5D)] |
| --- | --- | --- |
|  | [EST Profile](http://www.ncbi.nlm.nih.gov/UniGene/ESTProfileViewer.cgi?uglist=Hs.17910): | Approximate expression patterns inferred from EST sources. [[Show more entries with profiles like this](http://www.ncbi.nlm.nih.gov/sites/entrez?DB=unigene&DbFrom=unigene&IdsFromResult=133751&cmd=Link&LinkName=unigene_unigene_expression&tool=UniGene.clust)] |
|  | [GEO Profiles](http://www.ncbi.nlm.nih.gov/sites/entrez?DB=geoprofiles&DbFrom=unigene&IdsFromResult=133751&cmd=Link&LinkName=unigene_geoprofiles&tool=UniGene.clust): | Experimental gene expression data (Gene Expression Omnibus). |
|  | cDNA Sources: | mixed; placenta; brain; skin; eye; uncharacterized tissue; stomach; ovary; prostate |

**MAPPING POSITION**

Genomic location specified by transcript mapping, radiation hybrid mapping, genetic mapping or cytogenetic mapping.

|  | Chromosome: | 2 |  | |
| --- | --- | --- | --- | --- |
|  | UniSTS entry: | Chr 2 | [SHGC-33819](http://www.ncbi.nlm.nih.gov/genome/sts/sts.cgi?uid=15979) | [[Map Viewer](http://www.ncbi.nlm.nih.gov/mapview/maps.cgi?taxid=9606&chr=2&MAPS=wirh-r,ncbirh-r,shgcg3-r,tng-r,g3-r,gb4-r&sts=15979)] |
|  | UniSTS entry: | Chr 2 | [RH70496](http://www.ncbi.nlm.nih.gov/genome/sts/sts.cgi?uid=72467) |  |

**SEQUENCES**

*Sequences representing this gene; mRNAs, ESTs, and gene predictions supported by transcribed sequences.*

**EST sequences (18)**

|  | [BX102746.1](http://www.ncbi.nlm.nih.gov/UniGene/seq.cgi?ORG=Hs&SID=11128660) | Clone IMAGp998E22113_;_IMAGE:121341 | mixed |  |  |
| --- | --- | --- | --- | --- | --- |
|  | [AI360054.1](http://www.ncbi.nlm.nih.gov/UniGene/seq.cgi?ORG=Hs&SID=1241499) | Clone IMAGE:2018565 | brain | 3' read |  |
|  | [AI936609.1](http://www.ncbi.nlm.nih.gov/UniGene/seq.cgi?ORG=Hs&SID=1536708) | Clone IMAGE:2329620 | mixed | 3' read |  |
|  | [AI648552.1](http://www.ncbi.nlm.nih.gov/UniGene/seq.cgi?ORG=Hs&SID=1544341) | Clone IMAGE:2292504 | ovary | 3' read |  |
|  | [AI871280.1](http://www.ncbi.nlm.nih.gov/UniGene/seq.cgi?ORG=Hs&SID=1553072) | Clone IMAGE:2431258 | brain | 3' read |  |
|  | [BX365990.2](http://www.ncbi.nlm.nih.gov/UniGene/seq.cgi?ORG=Hs&SID=15540006) | Clone CS0DI066YC14 | placenta | 5' read |  |
|  | [BX360143.2](http://www.ncbi.nlm.nih.gov/UniGene/seq.cgi?ORG=Hs&SID=15542681) | Clone CS0DI066YC14 | placenta | 3' read |  |
|  | [BX360144.2](http://www.ncbi.nlm.nih.gov/UniGene/seq.cgi?ORG=Hs&SID=15543628) | Clone CS0DI066YC14 | placenta | 5' read |  |
|  | [AW975290.1](http://www.ncbi.nlm.nih.gov/UniGene/seq.cgi?ORG=Hs&SID=2009985) |  | uncharacterized tissue |  |  |
|  | [H40146.1](http://www.ncbi.nlm.nih.gov/UniGene/seq.cgi?ORG=Hs&SID=236396) | Clone IMAGE:191742 | mixed | 3' read |  |
|  | [ES310521.1](http://www.ncbi.nlm.nih.gov/UniGene/seq.cgi?ORG=Hs&SID=37953149) |  | skin |  | **A** |
|  | [BM684333.1](http://www.ncbi.nlm.nih.gov/UniGene/seq.cgi?ORG=Hs&SID=4100030) | Clone UI-E-EJ1-aji-d-10-0-UI | eye | 3' read | **A** |
|  | [BM829694.1](http://www.ncbi.nlm.nih.gov/UniGene/seq.cgi?ORG=Hs&SID=4225046) | Clone S5SNU484s1-9-D12 | stomach | 5' read | **A** |
|  | [BM832957.1](http://www.ncbi.nlm.nih.gov/UniGene/seq.cgi?ORG=Hs&SID=4228309) | Clone S5SNU484s1-10-G04 | stomach | 5' read | **A** |
|  | [BM929902.1](http://www.ncbi.nlm.nih.gov/UniGene/seq.cgi?ORG=Hs&SID=4669714) | Clone UI-E-EJ1-aji-d-10-0-UI | eye | 5' read |  |
|  | [T96871.1](http://www.ncbi.nlm.nih.gov/UniGene/seq.cgi?ORG=Hs&SID=84923) | Clone IMAGE:121341 | mixed | 3' read |  |
|  | [T98675.1](http://www.ncbi.nlm.nih.gov/UniGene/seq.cgi?ORG=Hs&SID=86857) | Clone IMAGE:122261 | mixed | 3' read |  |
|  | [AA659718.1](http://www.ncbi.nlm.nih.gov/UniGene/seq.cgi?ORG=Hs&SID=870489) | Clone IMAGE:1218923 | prostate | 3' read |  |

# EST Profile：

[Hs.17910](http://www.ncbi.nlm.nih.gov/UniGene/clust.cgi?ORG=Hs&CID=17910) - Transcribed locus

Breakdown by Body Sites

| brain | 1 | 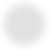 | 2 | / | 1092524 |
| --- | --- | --- | --- | --- | --- |
| eye | 9 | 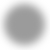 | 2 | / | 208810 |
| ovary | 9 | 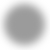 | 1 | / | 101482 |
| placenta | 10 | 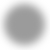 | 3 | / | 282968 |
| prostate | 5 | 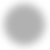 | 1 | / | 189585 |
| skin | 4 | 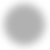 | 1 | / | 210718 |
| stomach | 20 | 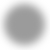 | 2 | / | 95775 |

Breakdown by Health State

| glioma | 18 | 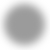 | 2 | / | 107167 |
| --- | --- | --- | --- | --- | --- |
| normal | 3 | 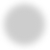 | 12 | / | 3328058 |

Breakdown by Developmental Stage

| fetus | 8 | 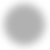 | 5 | / | 556801 |
| --- | --- | --- | --- | --- | --- |
| adult | 1 | 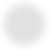 | 3 | / | 1921786 |

**BLAST result：**

| Accession | | Description | [Max score](http://blast.ncbi.nlm.nih.gov/Blast.cgi?CMD=Get&ALIGNMENTS=100&ALIGNMENT_VIEW=Pairwise&BLAST_SPEC=OGP__9606__9558&DATABASE_SORT=0&DESCRIPTIONS=100&FIRST_QUERY_NUM=0&FORMAT_OBJECT=Alignment&FORMAT_PAGE_TARGET=&FORMAT_TYPE=HTML&GET_SEQUENCE=yes&I_THRESH=&MASK_CHAR=2&MASK_COLOR=1&NEW_VIEW=yes&NUM_OVERVIEW=100&OLD_BLAST=false&PAGE=Nucleotides&QUERY_INDEX=0&QUERY_NUMBER=0&RESULTS_PAGE_TARGET=&RID=T5JCPGBY01S&SHOW_LINKOUT=yes&SHOW_OVERVIEW=yes&STEP_NUMBER=&WORD_SIZE=11&DISPLAY_SORT=1&HSP_SORT=1#sort_mark) | | [Total score](http://blast.ncbi.nlm.nih.gov/Blast.cgi?CMD=Get&ALIGNMENTS=100&ALIGNMENT_VIEW=Pairwise&BLAST_SPEC=OGP__9606__9558&DATABASE_SORT=0&DESCRIPTIONS=100&FIRST_QUERY_NUM=0&FORMAT_OBJECT=Alignment&FORMAT_PAGE_TARGET=&FORMAT_TYPE=HTML&GET_SEQUENCE=yes&I_THRESH=&MASK_CHAR=2&MASK_COLOR=1&NEW_VIEW=yes&NUM_OVERVIEW=100&OLD_BLAST=false&PAGE=Nucleotides&QUERY_INDEX=0&QUERY_NUMBER=0&RESULTS_PAGE_TARGET=&RID=T5JCPGBY01S&SHOW_LINKOUT=yes&SHOW_OVERVIEW=yes&STEP_NUMBER=&WORD_SIZE=11&DISPLAY_SORT=2&HSP_SORT=1#sort_mark) | [Query coverage](http://blast.ncbi.nlm.nih.gov/Blast.cgi?CMD=Get&ALIGNMENTS=100&ALIGNMENT_VIEW=Pairwise&BLAST_SPEC=OGP__9606__9558&DATABASE_SORT=0&DESCRIPTIONS=100&FIRST_QUERY_NUM=0&FORMAT_OBJECT=Alignment&FORMAT_PAGE_TARGET=&FORMAT_TYPE=HTML&GET_SEQUENCE=yes&I_THRESH=&MASK_CHAR=2&MASK_COLOR=1&NEW_VIEW=yes&NUM_OVERVIEW=100&OLD_BLAST=false&PAGE=Nucleotides&QUERY_INDEX=0&QUERY_NUMBER=0&RESULTS_PAGE_TARGET=&RID=T5JCPGBY01S&SHOW_LINKOUT=yes&SHOW_OVERVIEW=yes&STEP_NUMBER=&WORD_SIZE=11&DISPLAY_SORT=4&HSP_SORT=0#sort_mark) | | [E value](http://blast.ncbi.nlm.nih.gov/Blast.cgi?CMD=Get&ALIGNMENTS=100&ALIGNMENT_VIEW=Pairwise&BLAST_SPEC=OGP__9606__9558&DATABASE_SORT=0&DESCRIPTIONS=100&FIRST_QUERY_NUM=0&FORMAT_OBJECT=Alignment&FORMAT_PAGE_TARGET=&FORMAT_TYPE=HTML&GET_SEQUENCE=yes&I_THRESH=&MASK_CHAR=2&MASK_COLOR=1&NEW_VIEW=yes&NUM_OVERVIEW=100&OLD_BLAST=false&PAGE=Nucleotides&QUERY_INDEX=0&QUERY_NUMBER=0&RESULTS_PAGE_TARGET=&RID=T5JCPGBY01S&SHOW_LINKOUT=yes&SHOW_OVERVIEW=yes&STEP_NUMBER=&WORD_SIZE=11&DISPLAY_SORT=0&HSP_SORT=0#sort_mark) | | [Max ident](http://blast.ncbi.nlm.nih.gov/Blast.cgi?CMD=Get&ALIGNMENTS=100&ALIGNMENT_VIEW=Pairwise&BLAST_SPEC=OGP__9606__9558&DATABASE_SORT=0&DESCRIPTIONS=100&FIRST_QUERY_NUM=0&FORMAT_OBJECT=Alignment&FORMAT_PAGE_TARGET=&FORMAT_TYPE=HTML&GET_SEQUENCE=yes&I_THRESH=&MASK_CHAR=2&MASK_COLOR=1&NEW_VIEW=yes&NUM_OVERVIEW=100&OLD_BLAST=false&PAGE=Nucleotides&QUERY_INDEX=0&QUERY_NUMBER=0&RESULTS_PAGE_TARGET=&RID=T5JCPGBY01S&SHOW_LINKOUT=yes&SHOW_OVERVIEW=yes&STEP_NUMBER=&WORD_SIZE=11&DISPLAY_SORT=3&HSP_SORT=3#sort_mark) | | |  |
| --- | --- | --- | --- | --- | --- | --- | --- | --- | --- | --- | --- | --- | --- |
| [NG_032003.1](http://www.ncbi.nlm.nih.gov/nucleotide/372266088?report=genbank&log$=nucltop&blast_rank=36&RID=T79BF9X9012) | Homo sapiens ankyrin repeat domain 11 (ANKRD11), RefSeqGene on chromosome 16 | | | [39.2](http://blast.ncbi.nlm.nih.gov/Blast.cgi#372266088) | 39.2 | | 11% | | 5.3 | | 86% |  | |
| [NG_030466.1](http://www.ncbi.nlm.nih.gov/nucleotide/355390230?report=genbank&log$=nucltop&blast_rank=37&RID=T79BF9X9012) | Homo sapiens cadherin, EGF LAG seven-pass G-type receptor 1 (flamingo homolog, Drosophila) (CELSR1), RefSeqGene on chromosome 22 | | | [39.2](http://blast.ncbi.nlm.nih.gov/Blast.cgi#355390230) | 39.2 | | 14% | | 5.3 | | 80% |  | |
| [NG_029856.1](http://www.ncbi.nlm.nih.gov/nucleotide/345197223?report=genbank&log$=nucltop&blast_rank=38&RID=T79BF9X9012) | Homo sapiens carbohydrate (N-acetylgalactosamine 4-0) sulfotransferase 9 (CHST9), RefSeqGene on chromosome 18 | | | [39.2](http://blast.ncbi.nlm.nih.gov/Blast.cgi#345197223) | 39.2 | | 14% | | 5.3 | | 80% |  | |
| [NG_029184.1](http://www.ncbi.nlm.nih.gov/nucleotide/338221685?report=genbank&log$=nucltop&blast_rank=39&RID=T79BF9X9012) | Homo sapiens mitochondrial methionyl-tRNA formyltransferase (MTFMT), RefSeqGene on chromosome 15 | | | [39.2](http://blast.ncbi.nlm.nih.gov/Blast.cgi#338221685) | 39.2 | | 8% | | 5.3 | | 96% |  | |
| [NG_017093.1](http://www.ncbi.nlm.nih.gov/nucleotide/294610648?report=genbank&log$=nucltop&blast_rank=40&RID=T79BF9X9012) | Homo sapiens mastermind-like domain containing 1 (MAMLD1), RefSeqGene on chromosome X | | | [39.2](http://blast.ncbi.nlm.nih.gov/Blast.cgi#294610648) | 39.2 | | 9% | | 5.3 | | 90% |  | |
| [NG_016243.1](http://www.ncbi.nlm.nih.gov/nucleotide/283837925?report=genbank&log$=nucltop&blast_rank=41&RID=T79BF9X9012) | Homo sapiens gap junction protein, beta 4, 30.3kDa (GJB4), RefSeqGene on chromosome 1 | | | [39.2](http://blast.ncbi.nlm.nih.gov/Blast.cgi#283837925) | 39.2 | | 9% | | 5.3 | | 90% |  | |

1. **AK130682：**

[Lectin, galactoside-binding, soluble, 2](http://www.ncbi.nlm.nih.gov/UniGene/clust.cgi?UGID=1271674&TAXID=9606&SEARCH=AK130682)

LGALS2, Homo sapiens

Hs.531776: 46 sequences.

[Order cDNA clone](http://www.ncbi.nlm.nih.gov/genome/clone/orderclone.cgi?db=unigene&uid=1271674)

**Lectin, galactoside-binding, soluble, 2 (LGALS2)**

**SELECTED PROTEIN SIMILARITIES**

Comparison of cluster transcripts with RefSeq proteins. The alignments can suggest function of the cluster.

| **Best Hits and Hits from model organisms** | | **Species** | **Id(%)** | **Len(aa)** |
| --- | --- | --- | --- | --- |
| [NP_006489.1](javascript:PopUpMenu2_Set(Menu_prot5729903);) | galectin-2 | *H. sapiens* | 100.0 | 131 |
| [NP_079898.2](javascript:PopUpMenu2_Set(Menu_prot269914146);) | galectin-2 | *M. musculus* | 70.0 | 129 |
| **Other hits (15) [**[**Show subset**](http://www.ncbi.nlm.nih.gov/UniGene/clust.cgi?ORG=Hs&CID=531776)**]** | | **Species** | **Id(%)** | **Len(aa)** |
| [XP_525588.2](javascript:PopUpMenu2_Set(Menu_prot114686303);) | PREDICTED: galectin-2 | *P. troglodytes* | 99.2 | 131 |
| [XP_001087063.1](javascript:PopUpMenu2_Set(Menu_prot109094101);) | PREDICTED: galectin-2 | *M. mulatta* | 98.5 | 131 |
| [XP_001499566.1](javascript:PopUpMenu2_Set(Menu_prot149743269);) | PREDICTED: galectin-2-like | *E. caballus* | 83.1 | 129 |
| [XP_002723587.1](javascript:PopUpMenu2_Set(Menu_prot291414681);) | PREDICTED: lectin, galactoside-binding, soluble, 2 | *O. cuniculus* | 82.4 | 130 |
| [XP_531742.1](javascript:PopUpMenu2_Set(Menu_prot57092773);) | PREDICTED: galectin-2 isoform 1 | *C. lupus familiaris* | 80.0 | 129 |
| [NP_001136314.1](javascript:PopUpMenu2_Set(Menu_prot218664491);) | galectin-3 | *S. scrofa* | 79.4 | 130 |
| [NP_001244020.1](javascript:PopUpMenu2_Set(Menu_prot379991164);) | galectin-2 isoform 1 | *B. taurus* | 76.9 | 129 |
| [NP_598283.1](javascript:PopUpMenu2_Set(Menu_prot19424308);) | galectin-2 | *R. norvegicus* | 72.3 | 129 |
| [XP_001376472.2](javascript:PopUpMenu2_Set(Menu_prot334348012);) | PREDICTED: galectin-2-like | *M. domestica* | 62.5 | 127 |
| [XP_001510438.1](javascript:PopUpMenu2_Set(Menu_prot149632418);) | PREDICTED: galectin-2-like | *O. anatinus* | 60.9 | 127 |
| [XP_001234400.2](javascript:PopUpMenu2_Set(Menu_prot363727877);) | PREDICTED: galectin-2-like isoform 1 | *G. gallus* | 60.3 | 130 |
| [XP_003202284.1](javascript:PopUpMenu2_Set(Menu_prot326911881);) | PREDICTED: galectin-2-like isoform 1 | *M. gallopavo* | 59.5 | 130 |
| [NP_001116913.1](javascript:PopUpMenu2_Set(Menu_prot183986647);) | lectin, galactoside-binding, soluble, 2 | *X. tropicalis* | 55.7 | 130 |
| [XP_003443163.1](javascript:PopUpMenu2_Set(Menu_prot348511261);) | PREDICTED: galectin-2-like | *O. niloticus* | 45.5 | 134 |
| [NP_001135409.1](javascript:PopUpMenu2_Set(Menu_prot213688351);) | galectin-1 | *P. anubis* | 45.2 | 126 |

**GENE EXPRESSION**

Tissues and development stages from this gene's sequences survey gene expression. Links to other NCBI expression resources.

|  | [EST Profile](http://www.ncbi.nlm.nih.gov/UniGene/ESTProfileViewer.cgi?uglist=Hs.531776): | Approximate expression patterns inferred from EST sources. [[Show more entries with profiles like this](http://www.ncbi.nlm.nih.gov/sites/entrez?DB=unigene&DbFrom=unigene&IdsFromResult=1271674&cmd=Link&LinkName=unigene_unigene_expression&tool=UniGene.clust)] |
| --- | --- | --- |
|  | [GEO Profiles](http://www.ncbi.nlm.nih.gov/sites/entrez?DB=geoprofiles&DbFrom=unigene&IdsFromResult=1271674&cmd=Link&LinkName=unigene_geoprofiles&tool=UniGene.clust): | Experimental gene expression data (Gene Expression Omnibus). |
|  | cDNA Sources: | mixed; spleen; connective tissue; kidney; liver; uncharacterized tissue; bone marrow; mammary gland; heart; pharynx |

**MAPPING POSITION**

Genomic location specified by transcript mapping, radiation hybrid mapping, genetic mapping or cytogenetic mapping.

|  | Map position: | 22q12-q13|22q13.1 |  | |
| --- | --- | --- | --- | --- |
|  | UniSTS entry: | Chr 22 | [RH93754](http://www.ncbi.nlm.nih.gov/genome/sts/sts.cgi?uid=91837) |  |
|  | UniSTS entry: | Chr 1 | [GDB:631802](http://www.ncbi.nlm.nih.gov/genome/sts/sts.cgi?uid=158429) |  |
|  | UniSTS entry: | Chr 1 | [D12S1332](http://www.ncbi.nlm.nih.gov/genome/sts/sts.cgi?uid=48481) | [[Map Viewer](http://www.ncbi.nlm.nih.gov/mapview/maps.cgi?taxid=9606&chr=1&MAPS=thon-r,marsh-r&sts=48481)] |
|  | UniSTS entry: | Chr 1 | [D8S2278](http://www.ncbi.nlm.nih.gov/genome/sts/sts.cgi?uid=473906) |  |

**SEQUENCES**

*Sequences representing this gene; mRNAs, ESTs, and gene predictions supported by transcribed sequences.*

**mRNA sequences (9)**

|  | [M14079.1](http://www.ncbi.nlm.nih.gov/UniGene/seq.cgi?ORG=Hs&SID=1230) | Human HL14 gene encoding beta-galactoside-binding lectin, 3' end, clone 1 | **P** |
| --- | --- | --- | --- |
|  | [AK130682.1](http://www.ncbi.nlm.nih.gov/UniGene/seq.cgi?ORG=Hs&SID=16882611) | Homo sapiens cDNA FLJ27172 fis, clone SYN01847 |  |
|  | [BC059782.1](http://www.ncbi.nlm.nih.gov/UniGene/seq.cgi?ORG=Hs&SID=17083399) | Homo sapiens lectin, galactoside-binding, soluble, 2, mRNA (cDNA clone MGC:75071 IMAGE:5185491), complete cds | **PA** |
|  | [NM_006498.2](http://www.ncbi.nlm.nih.gov/UniGene/seq.cgi?ORG=Hs&SID=1731011) | Homo sapiens lectin, galactoside-binding, soluble, 2 (LGALS2), mRNA | **PA** |
|  | [CR456512.1](http://www.ncbi.nlm.nih.gov/UniGene/seq.cgi?ORG=Hs&SID=20247491) | Homo sapiens LGALS2 full length open reading frame (ORF) cDNA clone (cDNA clone C22ORF:pGEM.LGALS2.V3) | **P** |
|  | [CR542000.1](http://www.ncbi.nlm.nih.gov/UniGene/seq.cgi?ORG=Hs&SID=21075353) | Homo sapiens full open reading frame cDNA clone RZPDo834A1235D for gene LGALS2, lectin, galactoside-binding, soluble, 2 (galectin 2); complete cds, without stopcodon | **P** |
|  | [CR541972.1](http://www.ncbi.nlm.nih.gov/UniGene/seq.cgi?ORG=Hs&SID=21075380) | Homo sapiens full open reading frame cDNA clone RZPDo834E1234D for gene LGALS2, lectin, galactoside-binding, soluble, 2 (galectin 2); complete cds, incl. stopcodon | **P** |
|  | [M87842.1](http://www.ncbi.nlm.nih.gov/UniGene/seq.cgi?ORG=Hs&SID=2790) | Human S-lac lectin L-14-II (LGALS2) mRNA, complete cds | **P** |
|  | [BC029063.2](http://www.ncbi.nlm.nih.gov/UniGene/seq.cgi?ORG=Hs&SID=4436424) | Homo sapiens lectin, galactoside-binding, soluble, 2, mRNA (cDNA clone IMAGE:5189510), partial cds | **PA** |

**EST sequences (37)**

|  | [AA887588.1](http://www.ncbi.nlm.nih.gov/UniGene/seq.cgi?ORG=Hs&SID=1029086) | Clone IMAGE:1502167 | kidney | 3' read | **PA** |
| --- | --- | --- | --- | --- | --- |
|  | [AA927697.1](http://www.ncbi.nlm.nih.gov/UniGene/seq.cgi?ORG=Hs&SID=1043402) | Clone IMAGE:1552711 | uncharacterized tissue | 3' read | **PA** |
|  | [BX108632.1](http://www.ncbi.nlm.nih.gov/UniGene/seq.cgi?ORG=Hs&SID=11132202) | Clone IMAGp998J083725_;_IMAGE:1472743 | kidney |  | **PA** |
|  | [AI275539.1](http://www.ncbi.nlm.nih.gov/UniGene/seq.cgi?ORG=Hs&SID=1157391) | Clone IMAGE:1877056 | mixed | 3' read | **PA** |
|  | [AI244692.1](http://www.ncbi.nlm.nih.gov/UniGene/seq.cgi?ORG=Hs&SID=1182011) | Clone IMAGE:1866896 | kidney | 3' read | **PA** |
|  | [AI245421.1](http://www.ncbi.nlm.nih.gov/UniGene/seq.cgi?ORG=Hs&SID=1182740) | Clone IMAGE:1870388 | kidney | 3' read | **PA** |
|  | [AI288931.1](http://www.ncbi.nlm.nih.gov/UniGene/seq.cgi?ORG=Hs&SID=1197878) | Clone IMAGE:1878700 | mixed | 3' read | **PA** |
|  | [AI299922.1](http://www.ncbi.nlm.nih.gov/UniGene/seq.cgi?ORG=Hs&SID=1203696) | Clone IMAGE:1902506 | kidney | 3' read | **PA** |
|  | [AI378809.1](http://www.ncbi.nlm.nih.gov/UniGene/seq.cgi?ORG=Hs&SID=1257718) | Clone IMAGE:2069673 | mixed | 3' read | **PA** |
|  | [CD104013.1](http://www.ncbi.nlm.nih.gov/UniGene/seq.cgi?ORG=Hs&SID=15502817) | Clone IMAGE:30372148 | mixed | 5' read |  |
|  | [CD701729.1](http://www.ncbi.nlm.nih.gov/UniGene/seq.cgi?ORG=Hs&SID=16105145) |  | pharynx |  | **P** |
|  | [AW949542.1](http://www.ncbi.nlm.nih.gov/UniGene/seq.cgi?ORG=Hs&SID=1984237) |  | uncharacterized tissue |  | **PA** |
|  | [BP303532.1](http://www.ncbi.nlm.nih.gov/UniGene/seq.cgi?ORG=Hs&SID=21818078) | Clone MPE08817 | uncharacterized tissue | 5' read | **P** |
|  | [BP303533.1](http://www.ncbi.nlm.nih.gov/UniGene/seq.cgi?ORG=Hs&SID=21818079) | Clone MPE08818 | uncharacterized tissue | 5' read | **P** |
|  | [BP319592.1](http://www.ncbi.nlm.nih.gov/UniGene/seq.cgi?ORG=Hs&SID=21828897) | Clone PCD11229 | heart | 5' read | **PA** |
|  | [BE818590.1](http://www.ncbi.nlm.nih.gov/UniGene/seq.cgi?ORG=Hs&SID=2401055) |  | mammary gland |  |  |
|  | [DN998970.1](http://www.ncbi.nlm.nih.gov/UniGene/seq.cgi?ORG=Hs&SID=24654256) | Clone TC108567 | bone marrow | 5' read | **P** |
|  | [T28672.1](http://www.ncbi.nlm.nih.gov/UniGene/seq.cgi?ORG=Hs&SID=26068) |  | liver | 5' read | **P** |
|  | [DA957381.1](http://www.ncbi.nlm.nih.gov/UniGene/seq.cgi?ORG=Hs&SID=29055071) | Clone SPLEN2036681 | spleen | 5' read |  |
|  | [BG190992.1](http://www.ncbi.nlm.nih.gov/UniGene/seq.cgi?ORG=Hs&SID=3508854) |  | connective tissue |  | **P** |
|  | [BG198115.1](http://www.ncbi.nlm.nih.gov/UniGene/seq.cgi?ORG=Hs&SID=3515977) |  | connective tissue |  | **P** |
|  | [BG204453.1](http://www.ncbi.nlm.nih.gov/UniGene/seq.cgi?ORG=Hs&SID=3522187) |  | connective tissue |  | **P** |
|  | [BG216617.1](http://www.ncbi.nlm.nih.gov/UniGene/seq.cgi?ORG=Hs&SID=3534327) |  | connective tissue |  | **P** |
|  | [EL734953.1](http://www.ncbi.nlm.nih.gov/UniGene/seq.cgi?ORG=Hs&SID=37440806) |  | mixed | 5' read | **P** |
|  | [AU185862.1](http://www.ncbi.nlm.nih.gov/UniGene/seq.cgi?ORG=Hs&SID=3798061) | Clone B02379-007 | uncharacterized tissue |  | **P** |
|  | [BI760265.1](http://www.ncbi.nlm.nih.gov/UniGene/seq.cgi?ORG=Hs&SID=3892893) | Clone IMAGE:5185491 | mixed | 5' read | **P** |
|  | [BI762247.1](http://www.ncbi.nlm.nih.gov/UniGene/seq.cgi?ORG=Hs&SID=3894875) | Clone IMAGE:5189510 | mixed | 5' read | **P** |
|  | [BI833192.1](http://www.ncbi.nlm.nih.gov/UniGene/seq.cgi?ORG=Hs&SID=3920798) | Clone IMAGE:5226273 | mixed | 5' read |  |
|  | [BQ276959.1](http://www.ncbi.nlm.nih.gov/UniGene/seq.cgi?ORG=Hs&SID=4427195) | Clone IMAGE:5810046 | mixed | 5' read | **P** |
|  | [BU534138.1](http://www.ncbi.nlm.nih.gov/UniGene/seq.cgi?ORG=Hs&SID=4798905) | Clone IMAGE:6560942 | mixed | 5' read | **P** |
|  | [FN116606.1](http://www.ncbi.nlm.nih.gov/UniGene/seq.cgi?ORG=Hs&SID=52923213) | Clone 162504_0107_0857 |  |  |  |
|  | [BU585265.1](http://www.ncbi.nlm.nih.gov/UniGene/seq.cgi?ORG=Hs&SID=5467853) | Clone 71686450 | uncharacterized tissue |  |  |
|  | [BU584354.1](http://www.ncbi.nlm.nih.gov/UniGene/seq.cgi?ORG=Hs&SID=5468018) | Clone 358409 | connective tissue | 5' read |  |
|  | [CA449528.1](http://www.ncbi.nlm.nih.gov/UniGene/seq.cgi?ORG=Hs&SID=6154862) | Clone UI-H-EI1-ayt-h-18-0-UI | connective tissue | 3' read | **PA** |
|  | [AA872397.1](http://www.ncbi.nlm.nih.gov/UniGene/seq.cgi?ORG=Hs&SID=973063) | Clone IMAGE:1472743 | kidney | 3' read | **PA** |
|  | [AA894628.1](http://www.ncbi.nlm.nih.gov/UniGene/seq.cgi?ORG=Hs&SID=983404) | Clone IMAGE:1502463 | kidney | 3' read | **PA** |
|  | [AA917836.1](http://www.ncbi.nlm.nih.gov/UniGene/seq.cgi?ORG=Hs&SID=987837) | Clone IMAGE:1533763 | kidney | 3' read | **P** |

**BLAST result：**

| Accession | Description | [Max score](http://blast.ncbi.nlm.nih.gov/Blast.cgi?CMD=Get&ALIGNMENTS=100&ALIGNMENT_VIEW=Pairwise&BLAST_SPEC=OGP__9606__9558&DATABASE_SORT=0&DESCRIPTIONS=100&FIRST_QUERY_NUM=0&FORMAT_OBJECT=Alignment&FORMAT_PAGE_TARGET=&FORMAT_TYPE=HTML&GET_SEQUENCE=yes&I_THRESH=&MASK_CHAR=2&MASK_COLOR=1&NEW_VIEW=yes&NUM_OVERVIEW=100&OLD_BLAST=false&PAGE=Nucleotides&QUERY_INDEX=0&QUERY_NUMBER=0&RESULTS_PAGE_TARGET=&RID=T5JCPGBY01S&SHOW_LINKOUT=yes&SHOW_OVERVIEW=yes&STEP_NUMBER=&WORD_SIZE=11&DISPLAY_SORT=1&HSP_SORT=1#sort_mark) | [Total score](http://blast.ncbi.nlm.nih.gov/Blast.cgi?CMD=Get&ALIGNMENTS=100&ALIGNMENT_VIEW=Pairwise&BLAST_SPEC=OGP__9606__9558&DATABASE_SORT=0&DESCRIPTIONS=100&FIRST_QUERY_NUM=0&FORMAT_OBJECT=Alignment&FORMAT_PAGE_TARGET=&FORMAT_TYPE=HTML&GET_SEQUENCE=yes&I_THRESH=&MASK_CHAR=2&MASK_COLOR=1&NEW_VIEW=yes&NUM_OVERVIEW=100&OLD_BLAST=false&PAGE=Nucleotides&QUERY_INDEX=0&QUERY_NUMBER=0&RESULTS_PAGE_TARGET=&RID=T5JCPGBY01S&SHOW_LINKOUT=yes&SHOW_OVERVIEW=yes&STEP_NUMBER=&WORD_SIZE=11&DISPLAY_SORT=2&HSP_SORT=1#sort_mark) | [Query coverage](http://blast.ncbi.nlm.nih.gov/Blast.cgi?CMD=Get&ALIGNMENTS=100&ALIGNMENT_VIEW=Pairwise&BLAST_SPEC=OGP__9606__9558&DATABASE_SORT=0&DESCRIPTIONS=100&FIRST_QUERY_NUM=0&FORMAT_OBJECT=Alignment&FORMAT_PAGE_TARGET=&FORMAT_TYPE=HTML&GET_SEQUENCE=yes&I_THRESH=&MASK_CHAR=2&MASK_COLOR=1&NEW_VIEW=yes&NUM_OVERVIEW=100&OLD_BLAST=false&PAGE=Nucleotides&QUERY_INDEX=0&QUERY_NUMBER=0&RESULTS_PAGE_TARGET=&RID=T5JCPGBY01S&SHOW_LINKOUT=yes&SHOW_OVERVIEW=yes&STEP_NUMBER=&WORD_SIZE=11&DISPLAY_SORT=4&HSP_SORT=0#sort_mark) | [E value](http://blast.ncbi.nlm.nih.gov/Blast.cgi?CMD=Get&ALIGNMENTS=100&ALIGNMENT_VIEW=Pairwise&BLAST_SPEC=OGP__9606__9558&DATABASE_SORT=0&DESCRIPTIONS=100&FIRST_QUERY_NUM=0&FORMAT_OBJECT=Alignment&FORMAT_PAGE_TARGET=&FORMAT_TYPE=HTML&GET_SEQUENCE=yes&I_THRESH=&MASK_CHAR=2&MASK_COLOR=1&NEW_VIEW=yes&NUM_OVERVIEW=100&OLD_BLAST=false&PAGE=Nucleotides&QUERY_INDEX=0&QUERY_NUMBER=0&RESULTS_PAGE_TARGET=&RID=T5JCPGBY01S&SHOW_LINKOUT=yes&SHOW_OVERVIEW=yes&STEP_NUMBER=&WORD_SIZE=11&DISPLAY_SORT=0&HSP_SORT=0#sort_mark) | [Max ident](http://blast.ncbi.nlm.nih.gov/Blast.cgi?CMD=Get&ALIGNMENTS=100&ALIGNMENT_VIEW=Pairwise&BLAST_SPEC=OGP__9606__9558&DATABASE_SORT=0&DESCRIPTIONS=100&FIRST_QUERY_NUM=0&FORMAT_OBJECT=Alignment&FORMAT_PAGE_TARGET=&FORMAT_TYPE=HTML&GET_SEQUENCE=yes&I_THRESH=&MASK_CHAR=2&MASK_COLOR=1&NEW_VIEW=yes&NUM_OVERVIEW=100&OLD_BLAST=false&PAGE=Nucleotides&QUERY_INDEX=0&QUERY_NUMBER=0&RESULTS_PAGE_TARGET=&RID=T5JCPGBY01S&SHOW_LINKOUT=yes&SHOW_OVERVIEW=yes&STEP_NUMBER=&WORD_SIZE=11&DISPLAY_SORT=3&HSP_SORT=3#sort_mark) |
| --- | --- | --- | --- | --- | --- | --- |
| [NG_012096.1](http://www.ncbi.nlm.nih.gov/nucleotide/237858703?report=genbank&log$=nucltop&blast_rank=1&RID=T7AG9ZTB012) | Homo sapiens lectin, galactoside-binding, soluble, 2 (LGALS2), RefSeqGene on chromosome 22 | [2026](http://blast.ncbi.nlm.nih.gov/Blast.cgi#237858703) | 5190 | 100% | 0.0 | 99% |
| [NG_011881.1](http://www.ncbi.nlm.nih.gov/nucleotide/226874819?report=genbank&log$=nucltop&blast_rank=81&RID=T7AG9ZTB012) | Homo sapiens RNA binding protein, fox-1 homolog (C. elegans) 1 (RBFOX1), RefSeqGene on chromosome 16 | [334](http://blast.ncbi.nlm.nih.gov/Blast.cgi#226874819) | 428 | 18% | 5e-88 | 83% |
| [NG_007455.1](http://www.ncbi.nlm.nih.gov/nucleotide/170014701?report=genbank&log$=nucltop&blast_rank=92&RID=T7AG9ZTB012) | Homo sapiens exostosin 1 (EXT1), RefSeqGene on chromosome 8 | [322](http://blast.ncbi.nlm.nih.gov/Blast.cgi#170014701) | 506 | 20% | 3e-84 | 81% |

1. **BQ188778：**

[Transcribed locus](http://www.ncbi.nlm.nih.gov/UniGene/clust.cgi?UGID=1775681&TAXID=9606&SEARCH=BQ188778)

Homo sapiens

Hs.560028: 3 sequences.

**Transcribed locus**

**GENE EXPRESSION**

Tissues and development stages from this gene's sequences survey gene expression. Links to other NCBI expression resources.

|  | [EST Profile](http://www.ncbi.nlm.nih.gov/UniGene/ESTProfileViewer.cgi?uglist=Hs.560028): | Approximate expression patterns inferred from EST sources. |
| --- | --- | --- |
|  | [GEO Profiles](http://www.ncbi.nlm.nih.gov/sites/entrez?DB=geoprofiles&DbFrom=unigene&IdsFromResult=1775681&cmd=Link&LinkName=unigene_geoprofiles&tool=UniGene.clust): | Experimental gene expression data (Gene Expression Omnibus). |
|  | cDNA Sources: | eye; parathyroid |

**MAPPING POSITION**

Genomic location specified by transcript mapping, radiation hybrid mapping, genetic mapping or cytogenetic mapping.

|  | UniSTS entry: | Chr 12 | [RH47811](http://www.ncbi.nlm.nih.gov/genome/sts/sts.cgi?uid=35916) | [[Map Viewer](http://www.ncbi.nlm.nih.gov/mapview/maps.cgi?taxid=9606&chr=12&MAPS=ncbirh-r,gb4-r&sts=35916)] |
| --- | --- | --- | --- | --- |

**SEQUENCES**

*Sequences representing this gene; mRNAs, ESTs, and gene predictions supported by transcribed sequences.*

**EST sequences (3)**

|  | [CK301151.1](http://www.ncbi.nlm.nih.gov/UniGene/seq.cgi?ORG=Hs&SID=18092210) | Clone UI-E-EJ1-ajx-e-14-0-UI | eye | 3' read | **A** |
| --- | --- | --- | --- | --- | --- |
|  | [W04569.1](http://www.ncbi.nlm.nih.gov/UniGene/seq.cgi?ORG=Hs&SID=395675) | Clone IMAGE:320243 | parathyroid | 3' read | **A** |
|  | [BQ188778.1](http://www.ncbi.nlm.nih.gov/UniGene/seq.cgi?ORG=Hs&SID=4404120) | Clone UI-E-EJ1-ajx-e-14-0-UI | eye | 5' read | **A** |

**EST Profile：**

[Hs.560028](http://www.ncbi.nlm.nih.gov/UniGene/clust.cgi?ORG=Hs&CID=560028) - Transcribed locus

Breakdown by Body Sites

|  | [Hs.560028](http://www.ncbi.nlm.nih.gov/UniGene/clust.cgi?ORG=Hs&CID=560028) | | | | |
| --- | --- | --- | --- | --- | --- |
| eye | 9 | 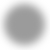 | 2 | / | 208810 |
| parathyroid | 48 | 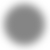 | 1 | / | 20579 |

**BLAST result：**

| Accession | Description | [Max score](http://blast.ncbi.nlm.nih.gov/Blast.cgi?CMD=Get&ALIGNMENTS=100&ALIGNMENT_VIEW=Pairwise&BLAST_SPEC=OGP__9606__9558&DATABASE_SORT=0&DESCRIPTIONS=100&FIRST_QUERY_NUM=0&FORMAT_OBJECT=Alignment&FORMAT_PAGE_TARGET=&FORMAT_TYPE=HTML&GET_SEQUENCE=yes&I_THRESH=&MASK_CHAR=2&MASK_COLOR=1&NEW_VIEW=yes&NUM_OVERVIEW=100&OLD_BLAST=false&PAGE=Nucleotides&QUERY_INDEX=0&QUERY_NUMBER=0&RESULTS_PAGE_TARGET=&RID=UMBX4T6W01N&SHOW_LINKOUT=yes&SHOW_OVERVIEW=yes&STEP_NUMBER=&WORD_SIZE=11&DISPLAY_SORT=1&HSP_SORT=1#sort_mark) | [Total score](http://blast.ncbi.nlm.nih.gov/Blast.cgi?CMD=Get&ALIGNMENTS=100&ALIGNMENT_VIEW=Pairwise&BLAST_SPEC=OGP__9606__9558&DATABASE_SORT=0&DESCRIPTIONS=100&FIRST_QUERY_NUM=0&FORMAT_OBJECT=Alignment&FORMAT_PAGE_TARGET=&FORMAT_TYPE=HTML&GET_SEQUENCE=yes&I_THRESH=&MASK_CHAR=2&MASK_COLOR=1&NEW_VIEW=yes&NUM_OVERVIEW=100&OLD_BLAST=false&PAGE=Nucleotides&QUERY_INDEX=0&QUERY_NUMBER=0&RESULTS_PAGE_TARGET=&RID=UMBX4T6W01N&SHOW_LINKOUT=yes&SHOW_OVERVIEW=yes&STEP_NUMBER=&WORD_SIZE=11&DISPLAY_SORT=2&HSP_SORT=1#sort_mark) | [Query coverage](http://blast.ncbi.nlm.nih.gov/Blast.cgi?CMD=Get&ALIGNMENTS=100&ALIGNMENT_VIEW=Pairwise&BLAST_SPEC=OGP__9606__9558&DATABASE_SORT=0&DESCRIPTIONS=100&FIRST_QUERY_NUM=0&FORMAT_OBJECT=Alignment&FORMAT_PAGE_TARGET=&FORMAT_TYPE=HTML&GET_SEQUENCE=yes&I_THRESH=&MASK_CHAR=2&MASK_COLOR=1&NEW_VIEW=yes&NUM_OVERVIEW=100&OLD_BLAST=false&PAGE=Nucleotides&QUERY_INDEX=0&QUERY_NUMBER=0&RESULTS_PAGE_TARGET=&RID=UMBX4T6W01N&SHOW_LINKOUT=yes&SHOW_OVERVIEW=yes&STEP_NUMBER=&WORD_SIZE=11&DISPLAY_SORT=4&HSP_SORT=0#sort_mark) | [E value](http://blast.ncbi.nlm.nih.gov/Blast.cgi?CMD=Get&ALIGNMENTS=100&ALIGNMENT_VIEW=Pairwise&BLAST_SPEC=OGP__9606__9558&DATABASE_SORT=0&DESCRIPTIONS=100&FIRST_QUERY_NUM=0&FORMAT_OBJECT=Alignment&FORMAT_PAGE_TARGET=&FORMAT_TYPE=HTML&GET_SEQUENCE=yes&I_THRESH=&MASK_CHAR=2&MASK_COLOR=1&NEW_VIEW=yes&NUM_OVERVIEW=100&OLD_BLAST=false&PAGE=Nucleotides&QUERY_INDEX=0&QUERY_NUMBER=0&RESULTS_PAGE_TARGET=&RID=UMBX4T6W01N&SHOW_LINKOUT=yes&SHOW_OVERVIEW=yes&STEP_NUMBER=&WORD_SIZE=11&DISPLAY_SORT=0&HSP_SORT=0#sort_mark) | [Max ident](http://blast.ncbi.nlm.nih.gov/Blast.cgi?CMD=Get&ALIGNMENTS=100&ALIGNMENT_VIEW=Pairwise&BLAST_SPEC=OGP__9606__9558&DATABASE_SORT=0&DESCRIPTIONS=100&FIRST_QUERY_NUM=0&FORMAT_OBJECT=Alignment&FORMAT_PAGE_TARGET=&FORMAT_TYPE=HTML&GET_SEQUENCE=yes&I_THRESH=&MASK_CHAR=2&MASK_COLOR=1&NEW_VIEW=yes&NUM_OVERVIEW=100&OLD_BLAST=false&PAGE=Nucleotides&QUERY_INDEX=0&QUERY_NUMBER=0&RESULTS_PAGE_TARGET=&RID=UMBX4T6W01N&SHOW_LINKOUT=yes&SHOW_OVERVIEW=yes&STEP_NUMBER=&WORD_SIZE=11&DISPLAY_SORT=3&HSP_SORT=3#sort_mark) |
| --- | --- | --- | --- | --- | --- | --- |
| [NG_028060.1](http://www.ncbi.nlm.nih.gov/nucleotide/317165590?report=genbank&log$=nucltop&blast_rank=19&RID=UMBX4T6W01N) | Homo sapiens dedicator of cytokinesis 4 (DOCK4), RefSeqGene on chromosome 7 | [48.2](http://blast.ncbi.nlm.nih.gov/Blast.cgi#317165590) | 48.2 | 5% | 0.028 | 86% |
| [NG_013249.1](http://www.ncbi.nlm.nih.gov/nucleotide/261823972?report=genbank&log$=nucltop&blast_rank=45&RID=UMBX4T6W01N) | Homo sapiens odz, odd Oz/ten-m homolog 1 (Drosophila) (ODZ1), RefSeqGene on chromosome X | [44.6](http://blast.ncbi.nlm.nih.gov/Blast.cgi#261823972) | 44.6 | 4% | 0.34 | 94% |
| [NG_009200.1](http://www.ncbi.nlm.nih.gov/nucleotide/219362532?report=genbank&log$=nucltop&blast_rank=75&RID=UMBX4T6W01N) | Homo sapiens API5 pseudogene 1 (API5P1) on chromosome X | [41.0](http://blast.ncbi.nlm.nih.gov/Blast.cgi#219362532) | 41.0 | 7% | 4.2 | 80% |
| [NG_001538.3](http://www.ncbi.nlm.nih.gov/nucleotide/169646386?report=genbank&log$=nucltop&blast_rank=76&RID=UMBX4T6W01N) | Homo sapiens proteasome (prosome, macropain) subunit, beta type, 3 pseudogene 2 (PSMB3P2) on chromosome 2 | [41.0](http://blast.ncbi.nlm.nih.gov/Blast.cgi#169646386) | 41.0 | 6% | 4.2 | 78% |
